# Revealing Hidden Conformational Space of LOV Protein VIVID Through Rigid Residue Scan Simulations

**DOI:** 10.1038/srep46626

**Published:** 2017-04-20

**Authors:** Hongyu Zhou, Brian D. Zoltowski, Peng Tao

**Affiliations:** 1 Department of Chemistry, Center for Drug Discovery, Design, and Delivery(CD4), Center for Scientific Computation, Southern Methodist University, Dallas, Texas 75275, United States of America

## Abstract

VIVID(VVD) protein is a Light-Oxygen-Voltage(LOV) domain in circadian clock system. Upon blue light activation, a covalent bond is formed between VVD residue Cys108 and its cofactor flavin adenine dinucleotide(FAD), and prompts VVD switching from Dark state to Light state with significant conformational deviation. However, the mechanism of this local environment initiated global protein conformational change remains elusive. We employed a recently developed computational approach, rigid residue scan(RRS), to systematically probe the impact of the internal degrees of freedom in each amino acid residue of VVD on its overall dynamics by applying rigid body constraint on each residue in molecular dynamics simulations. Key residues were identified with distinctive impacts on Dark and Light states, respectively. All the simulations display wide range of distribution on a two-dimensional(2D) plot upon structural root-mean-square deviations(RMSD) from either Dark or Light state. Clustering analysis of the 2D RMSD distribution leads to 15 representative structures with drastically different conformation of N-terminus, which is also a key difference between Dark and Light states of VVD. Further principle component analyses(PCA) of RRS simulations agree with the observation of distinctive impact from individual residues on Dark and Light states.

Light is a ubiquitous environmental signal of metabolism regulation for the majority of lives on earth. Light, Oxygen, Voltage(LOV) photoreceptor domains, first designated in 1997 are small^1^, commutable proteins, and couple blue-light triggered control mechanism in response to the light stimulation^2^. The LOV domains are involved with control of phototropism^1^,^3^, chloroplast relocation^4^, stomatal opening^5^, rapid inhibition of stem growth^6^, and gametogenesis^7^, for higher plants, and circadian temporal regulation in bacteria and fungi. LOV domains are present in many multi-domain proteins, including DNA binding domains(i.e., leucine zipper, bHLH, and zinc finger)^8^, STAS domains^9^, and kinases^10^,^11^,^12^, and form a subset of Per, ARNT, Sim(PAS) superfamily, a sensor module found in all three kingdoms of life^13^. All LOV domains contain common flavin chromophore, which could covalently bond to an adjacent cysteine residue upon blue-light activation. Due to their ubiquitous and modular nature in sensory proteins, LOV domains have been exploited widely for the design of optogenetic tools^14^.

VIVID(VVD) protein is a LOV domain that regulates filamentous fungus *Neurospora crassa* circadian system. In *Neurospora*, VVD and another LOV domain, White Collar-1(WC-1) are involved with light responses regulation. WC-1 forms a complex(referred as WCC) with nonphotosensitive WC-2 to activate transcription of the clock oscillator protein Frequency(FRQ). The main function of VVD is forming an autoregulatory negative feedback loop that connects the molecular output of circadian clock back to the input of the clock, which is also referred as “gating”^15^. Gating regulation is necessary for the appropriate response to daily light intensity fluctuation^16^,^17^,^18^. Photo-activated VVD reduces activation of the WCC to tune the *Neurospora* blue-light response. Flavin adenine dinucleotide(FAD) is required by both VVD and WC-1 for their light sensing functions. Some other LOV proteins, for example blue-light receptors in plants, phototropins, employ flavin mononucleotide(FMN) as chromophore for their light sensing function^17^,^19^,^20^,^21^.

The mechanisms that LOV domains respond to light activation and send signals to other parts of organisms are of great general interest. The common feature of LOV domains photo activation is that upon blue-light activation, the chromophore, either FAD or FMN, forms covalent bond with a cysteine residue of LOV domains, and induces a large conformational change within the LOV domain, thereby regulating activity of accessory signaling domains or protein:protein interactions. The states before and after photo activations could be referred as Dark and Light states, respectively. Using nuclear magnetic resonance(NMR) spectroscopy, Harper *et al*. showed that a 20 residues long helix, designated as Jα, undergoes conformational change upon photoactivation of *Avena sativa* Phototropin1 LOV2(AsPhot1 LOV2) domain^22^,^23^. Similar conformational change upon photoactivation was directly observed through crystallographic characterization of VVD Dark and Light states. Zoltowski *et al*. showed that photoinduced formation of a covalent bond between Cys108 sulfur and C4a position of FAD drives an N-terminal cap conformational change critical for VVD function (Fig. 1)^24^.

Interestingly, the VVD N-terminal cap locates at a position similar to the Jα helix in LOV2 domain, indicating possible conservation of an allosteric pathway. Through time-resolved small-angle X-ray scattering analysis, Lamb *et al*. revealed another VVD Dark state structure with extended N-terminal cap in addition to the previously identified compact Dark state structure^25^. In the crystallographic structure of VVD Light state, the N-terminal cap is driven away from the protein core into a more extended structure, which leads to VVD dimerization^26^. A conserved residue Cys71, critical for VVD conformational change upon photo activation, is further away from highly conserved Cys108(S-S distance as 14 Å), while a much closer Cys76 towards Cys108(S-S distance as 6 Å) has no effect on VVD function with mutation Cys76Ser displaying same activity as the wild type *in vivo*^24^. It is also reported that mutation Cys71Ser does not display detectable conformational change upon photo activation^24^. Further study revealed that mutation Cys71Val induces conformational change of VVD similar to the photo activated state of wild type VVD. However Cys71Val VVD does not function as the light-excited VVD.

LOV domains have been subjected to molecular dynamics(MD) simulation studies to explore the mechanisms of switching between Dark and Light states upon photoactivation. Peter *et al*. carried out MD simulations of AsPhot1 LOV2 domain and showed that the Jα helix could be driven away from the protein core through disruption of location intramolecular interaction upon formation of covalent bond between cysteine and FMN^27^. They also simulated LOV1 domain of *Chlamydomonas reinhardtii* and found out that the covalent bond between Cys57 and FMN induced by photoactivation could perturb salt bridge interaction between Glu51 and Lys91 through backbone chain^28^. They pointed out that the local structural perturbation impacts the dynamical behavior of hydrophobic and hydrophilic sides of LOV1 differently. Based on extensive MD simulations of AsPhot1 LOV2, Freddolino *et al*. showed that the dynamics of a conserved residue Glu513 are coupled with Jα helix through hydrogen bond interaction and alter the overall dynamical correlation patterns of whole protein^29^. Peter *et al*. carried out MD simulations of VVD Dark and Light states as monomer and dimer, and suggested that residue Gln182 close to FAD plays an important role in the transition of VVD from Dark state to Light state through hydrogen bonding interactions after the formation of photo-induced covalent bond between Cys108 and FAD^30^.

The significant conformational changes of these proteins upon photoactivation are features shared by many LOV domains leading to their wide usage in optogenetic tools. Currently the utility of these devices is limited due to residual activity in the typically inhibitory dark-state. Ideally, LOV allostery could be tuned to create an inactive off-state that digitally switches to a highly functional on-state in response to the lighting conditions. To do so it is essential to identify allosteric sites to target for mutations with substantial impact on overall protein dynamical behavior or structure. For example, the Cys71Val mutant of VVD resides in an intermediate structure that may sample space along the allosteric trajectory between VVD’s Dark and Light states^31^. Although numerous residues and related mutations were under investigations for LOV domains, a systematic investigation of all the residues of LOV domain is yet to be taken place for their potential contribution to overall protein dynamics and conformational distributions. To identify key residues that link and manipulate Dark-Light state trajectories a systematic analysis of the contribution of all residues to conformational landscape is needed.

Recently, we have developed a simulation method, referred to as rigid residue scan(RRS) to systematically estimate the impact from residues on overall protein dynamics by exerting rigid body constraint^32^ on each individual residue in separate MD simulations^33^,^34^. The examples presented in the RRS development articles demonstrated the effectiveness of this method with good agreement with regard to experimental observations. One of the advantages of RRS method is that no arbitrary mutations are needed to perturb each residue to reveal its contribution to overall protein dynamics. Instead, the rigid body constraint completely removes the internal dynamics of individual residues while the chemical content of the wild type residue remains unchanged throughout the simulations.

In the study presented here, we applied RRS method on the crystallographic structures of VVD Dark and Light states to systematically compare the impact of each individual residue on overall protein dynamics, and thoroughly explore potential conformational space that is reachable for VVD protein and could serve as guidance for further mutagenesis studies of this protein.

## Results

### Root-Mean-Square Deviation (RMSD)

The mass weighted RMSD of all atoms for Dark and Light states of VVD are plotted in Fig. 2A. Initially, the Light state has higher RMSD values than the Dark state agreeing with the expectation that Light state is more flexible than the Dark state. However, the RMSD values of the Dark state exceed the Light state around 120 ns of simulation, suggesting a larger conformational space accessible for the Dark state than the Light state. To illustrate and compare the conformational distributions of both Dark and Light states, we projected two simulations onto a two dimensional(2D) contour plots using RMSD values with reference to the optimized Dark and Light states, respectively (Fig. 2B). The unperturbed Dark and Light state simulations display different distributions on the 2D RMSD plots. For 210 ns of simulations, the Light state displays only one basin of attraction, but the Dark state displays three major basins of attraction, with the far right one adjacent to the Light state on the same plot.

The 1D and 2D RMSD plots of RRS simulations(all 30 ns) are provided in Supplementary Data(Tables S1 and S2). The RRS simulations of the Dark and Light states with the same residue being held rigid are projected on the same plot. As comparison, the projections of the first 30 ns unperturbed MD simulations are listed as well. For trajectories carried out in most RRS simulations, the Light state is more flexible than the Dark state with higher RMSD values in 1D plots. The 2D RMSD plots of these RRS simulations reveal more interesting details about the impact of rigid body on overall protein dynamics of two states. For the unperturbed states, the Dark and Light states are well separated (Fig. 3A), suggesting that the Dark and Light state structures are well reserved during the course of 30 ns simulations, respectively. Although the 2D RMSD plots are similar to the unperturbed states for many RRS simulations, some rigid residue perturbations dramatically change the overall dynamics of proteins in very different ways(Table S2). For example, when residue Glu112 is treated as rigid body during simulations, the distributions of both Dark and Light states on 2D RMSD plot shift towards each other and have significant overlap (Fig. 3B). When residue Asn119 is treated as rigid body during simulations, the distribution of the Light state on 2D RMSD plot shifts towards the Dark state, while the distribution of the Dark state trajectory remains close to the optimized Dark state as reference structure (Fig. 3C). In a third case, when residue Val124 is treated as rigid body during simulations, the distribution of the Dark state on 2D RMSD plot shifts towards the Light state, while the distribution of the Light state trajectory remains close to the optimized Light state as reference structure (Fig. 3D). These cases represent significantly different protein responses under rigid body perturbations, which could shed light on individual key allosteric residues.

RRS 112, 119 and 124 simulations are not the only cases displaying distinctive perturbation results. Other RRS simulations displaying similar effect in the 2D RMSD distributions are listed in Table 1. For more than thirty residues, the rigid residue perturbations mainly affect Light state only. For only handful residues, the rigid residue perturbations exert significant impact on Dark state. Comparison with the literature reveals that ten residues among those selected to affect mainly Light state in Table 1 have been reported in previous VVD experimental studies. Four residues were reported in the first crystallographic study of VVD^24^, and further confirmed by a crystallographic study of fully light-adapted VVD dimer^26^. Pro30 was reported to display largest shift(2.0 Å) in the Light state comparing to the Dark state.(In the literature of experimental study of VVD, the residue index is 36 larger than the index used in this study. Therefore Pro30 is referred as Pro66 in the cited articles. This also applies for the following residues in this paragraph.) It was suggested that a mutation at Ile16 prevents conformational switching associated with Light state. Another selected residue, Met19 is reported to stabilize packing of the N-terminal cap against the PAS β sheet. Asp46 participates in a salt bridge, which was suggested to mediate conformational changes of related LOV domains. Five residues were reported in a mechanistic study of VVD dimerization^31^. Change of Pro6 orientation interrupts key backbone interactions and facilitates the projection of the N-terminus position in the VVD dimer. A mutation at Met19 prevents the forming of VVD cross-linked dimer. On the contrary to Met19, a mutation of Glu135 leads to enhanced dimerization of VVD, suggesting that this position is also critical to VVD function. Another two selected residues Val132 and Tyr141 are located at a hydrophobic pocket that close to N-terminus, which contributes to the propagation of conformational changes triggered by light activation of VVD. In another experimental study of VVD Light state dimer using hydrogen exchange mass spectrometry^35^, two residues, Tyr51 and Ile16, are identified to contribute to asymmetry of two VVD units in its Light state dimer, which may be related to allosteric interaction of two VVD units. In a recent experimental study^36^, mutation of Met129, also listed in Table 1, was reported to stabilize VVD Light state. The residues listed above are not directly located at the flavin binding site, and could allosterically contribute to the VVD function. This certainly enhances the confidence level of the remaining residues, which could be used as guidance for potential target of further mechanistic studies. Due to the relatively short history of VVD study, it is unsurprising that not all of the residues listed in Table 1 have been tested and reported in the literature. Intriguingly, all of the residues covered by experiments belong to the group mainly affects Light state in Table 1. There are much fewer residues in the other two groups, which mainly affect either Dark state only or both states. The reason that residues from these two groups have yet to be tested and reported is likely because it is difficult to intuitively identify and select these residues. The seven residues listed under these two groups in Table 1 actually provide such targets for further studies, which may lead to new direction of VVD studies.

### Clustering analysis

To explore the overall distribution of the simulations, all the RRS and unperturbed simulations of VVD Dark and Light states are plotted on a 2D RMSD plot against Dark and Light crystallographic structures. The overall distribution on the 2D RMSD plot is large and diverse (Fig. 4A). The unperturbed Dark and Light states distribute only on the small fraction of the overall distribution. To facilitate the analysis of distribution on 2D RMSD plot, clustering analysis was carried out for the 2D RMSD distribution, and resulted in a total of 15 clusters. The distributions of Dark and Light state simulations among 15 clusters are plotted in Fig. 4B. All the RRS and unperturbed Dark state simulations mainly dwell in the cluster 1, and with significant contribution to clusters 2 and 3 (Fig. 4B). The RRS and unperturbed Light state simulations contribute significantly to clusters 2 through 10. The diversity of Light state contributions to the different clusters is consistent with the Light state being more dynamic, thereby enabling sampling of diverse states. Further, the overlap of contributions between clusters 2 and 3 in both the Light and the Dark states indicates fluidity in the conformational landscape of the two proteins. These states may contribute to residual activity of LOV proteins in the Dark state that inhibits the fidelity of optogenetic tools^14^.

The contributions from individual RRS simulations in each cluster are also calculated and plotted (Fig. 5). Most RRS Dark state simulations distribute in Cluster 1 (Fig. 5). But for some of Dark state RRS simulations, the contribution from Cluster 1 is below 50%, showing the significant impact on overall dynamics from perturbations on individual residues. It should be noted that the distributions of most RRS Dark state simulations in Cluster 1 are close to 100%. Therefore, the plot for RRS Dark state simulations(blue line in Fig. 5, Cluster 1) seems to be upside-down, which is not the case. All the RRS Light state, except for RRS119 Light state simulations, have almost no contribution to Cluster 1 (Fig. 5).

Cluster 2, adjacent to Cluster 1, has dominant RRS Dark state simulations distribution and significant contributions from numerous RRS Light state simulations. Cluster 3, adjacent to Cluster 2, has dominant RRS Light state simulations distribution and significant contributions from numerous RRS Dark state simulations. These results are consistent with the data from unperturbed states, where clusters 2 and 3 had contributions in both the Light and the Dark states, indicating these clusters may lie on the conformational trajectory between the Light and Dark states and thereby, contribute to the lack of a digital switch in optogenetic tools. Cluster 6 is also adjacent to Cluster 1, and has dominant RRS Light state simulations distribution and significant contributions from numerous RRS Dark state simulations, similar to Cluster 3. However, Cluster 6 has far less overall distribution from RRS simulations than Clusters 2 and 3.

On the contrary, Clusters 4, 5, 7, 8, and 9 only have significant contribution from RRS Light state simulations, without noticeable contribution from any Dark state simulations. These five clusters are aggregated together at the bottom of the distribution map (Fig. 4A). Clusters 10 through 15 only have significant contribution from a few RRS Light state simulations, and there is no noticeable contribution from any RRS Dark state simulations. These last six clusters cover the top right region of the cluster maps (Fig. 4A). Therefore, the 15 clusters could be divided into the following three groups. Group 1 comprising Clusters 1, 2, 3, and 6 can be considered as the main distribution area for the RRS Dark state. Group 2 comprising Clusters 4, 5, 7, 8, and 9 can be considered as the main distribution area for the RRS Light state. Group 3 comprising Clusters 10, 11, 12, 13, 14, and 15 are the rare distributions, which are accessed mainly by some RRS Light simulations. Representative structures were selected for each cluster and are illustrated for clusters in the above three groups (Fig. 6).

The distributions of unperturbed and RRS 112, 119, and 124 simulations among the above 15 clusters are listed in Table 2. The 210 ns unperturbed Dark state simulations have more than or close to 10% distribution in Clusters 1, 2, 3, 4, 5, and 9, and spans over Group 1 and 2 of clusters. It also has more than 1% distribution in Clusters 7, 8, and 10. On the other hand, the 210 ns unperturbed Light state simulations have more than 10% distribution in Clusters 9, 10, and 13. It also has more than 1% distribution in Clusters 7, 8, 14, and 15. Therefore the distribution of the unperturbed Light state simulations spans over Groups 2 and 3 of clusters and cover the corridor of the cluster map located at the right hand side (Fig. 4A). It should be noted that both unperturbed simulations have nontrivial distributions in Clusters 7, 8, 9, and 10, indicating the overlap between unperturbed Dark and Light states. Distributions of all RRS simulations among 15 clusters are listed in Table S3.

The impacts from rigid residue constraints on the distribution of Dark and Light states are very different(Table 2). With a rigid residue 112, the Dark state mainly distributes in Clusters 1, 2, and 3(Group 1) with more than 10% contribution from each cluster. However, with a rigid residue 112, the Light state mainly distributes in Clusters 3 and 4(Groups 1 and 2). As comparison, the unperturbed Light state simulation has 0.0% contribution from Clusters 3 and 4. Therefore, the rigid residue 112 caused a complete switching of Light state from the unperturbed Light state distributions. With a rigid residue 119, 99.8% of Dark state mainly distributes in Cluster 1(Group 1); and the Light state mainly distribute in Clusters 1 and 2(Group 1), also a complete switching from the unperturbed Light state distributions. With a rigid residue 124, the Dark state mainly distributes in Clusters 1, 2, and 3(Group 1) with more than 10% contribution from each cluster; and the Light state mainly distributes in Cluster 7(93.5%) and marginally in Cluster 8(6.5%)(Group 2). Apparently, perturbations on different residues have drastically different and subtle impact on overall dynamics of both Dark and Light states.

### Correlation motion

Cross-correlation matrix has been widely used to probe protein allosteric mechanisms. The heat maps of cross-correlation matrices for Dark and Light states from unperturbed and RRS simulations with residues 112, 119, 124 being held rigid are illustrated in Fig. 7. The heat maps and histograms of all RRS as well as 30 ns unperturbed simulations are listed in Table S4.

For the unperturbed simulations, although the basic features of heat maps are somewhat similar between Dark and Light states, the Light state has more prominent positive and negative correlations than the Dark state (Fig. 7B). These correlation features are even more prominent for RRS 112 simulations, especially the Light state (Fig. 7D). The correlation matrix heat maps for RRS119 simulations (Fig. 7E and F) are similar to the ones of their corresponding unperturbed simulations (Fig. 7A and B), respectively. However, the correlation matrix heat map for RRS124 Dark state simulation (Fig. 7G) is similar to the one of unperturbed Light state (Fig. 7B), while the one of RRS124 Light state simulation (Fig. 7H) is similar to the one of unperturbed Dark state (Fig. 7A), suggesting tendency of switching between Dark and Light states. It should be noted that many other RRS simulations also display similar correlations of VVD structure either more or less prominent in terms of correlations among different parts(Table S4).

The patterns of different correlations among different parts of VVD protein indicate that the protein structure could be divided into different regions with distinguished dynamical behavior. Using the correlation matrix heat map of RRS 112 Light state as guidance, VVD structure could be divided into seven regions (Fig. 8): Region 1(residues 1 through 5) is the N-terminus, region 2(residues 6 though 30, including α-helix and loop structure), region 4(residues 35 through 83, including two β-sheets and two short α-helices and loops connecting them), region 5(residues 84 through 108 including α-helix and loop structures), region 6(residues 109 through 131 including two β-sheets and a loop in between), region 7(residues 132 through 138 as a loop structure), and region 8(residues 139 through 148) as the C-terminus. Region 1 as N-terminus displays clear negative correlation with regions 2, 4, 6, and 8, and positive correlation with regions 3 and 5. On the contrary, C-terminus region 8 with a β-sheet structure displays clear positive correlation with regions 2, 4, and 6, and clear negative correlation with regions 1, 3, and 5. The most striking feature is arguably region 4 as the largest self positively correlated region. Overall regions 2, 4, 6, and 8 are positively correlated with each other, but are somewhat negatively correlated with regions 1, 3, 5, and 7.

### Protein configurational entropy

Configurational entropies(referred as entropy) of VVD are calculated through quasi-harmonic analysis for each simulation. The 30 ns trajectories of unperturbed simulations were used for the entropy calculations for the consistency with regard to RRS simulations. For the convenience of comparison, the entropy of the unperturbed Dark state simulation was used as reference for all other simulations, including both Dark and Light states. The relative VVD entropies are plotted in Fig. 9 and listed in Table S5 and with ascending order in Table S6.

For the unperturbed simulations, the Light state has entropy higher than the Dark state by 0.215 kcal/mol · K. For the RRS simulations of Dark state, the relative entropies range from −0.122 kcal/mol · K(RRS 141) to 0.238 kcal/mol · K(RRS 109). For 123 Dark state RRS simulations, the entropy of VVD protein is higher than the one from the unperturbed simulation, while for the other 25 Dark state RRS simulations, the entropy of VVD is lower. The relative VVD entropies from the most RRS simulations of Light state(with reference to the unperturbed Dark state simulation) are positive. However, when comparing to the unperturbed Light state simulation, total of 118 RRS simulations of Light state have lower VVD entropies(horizontal line in Fig. 9). This shows that the entropic response from the Light state to the rigid residue perturbation is intrinsically different from the Dark state.

The VVD entropy differences between Dark and Light states when the same residue is held rigid are plotted in Fig. 10 and also listed in Tables S5 and S6. For both RRS 112 and 119 simulations, the differences between Dark and Light state entropies are rather small as 0.007 and 0.004 kcal/mol · K, respectively, and are among the smallest of all RRS simulations(Table S6). RRS124, on the other hand, is the simulation with the most entropy decrease of Light state simulation comparing to its Dark state simulation(−0.225 kcal/mol · K). Clearly, rigid body perturbation on residue 124 has opposite effect on Dark and Light state entropies.

### Entropy contribution from individual residue

The entropy contribution from each individual residue in each simulation was calculated through the analysis of the cross-correlation matrices. For each RRS Dark state simulation, the relative entropy contribution from each individual residue was calculated with reference to the entropy contribution from the same residue in the unperturbed simulation of the VVD Dark state. The same calculation was also carried out for the RRS Light state simulations. The relative individual residue entropies are plotted as heat maps for RRS simulations of Dark and Light states in Fig. 11. The blue diagonal lines in both plots are due to the fact that the entropies from residues as rigid body is significantly smaller than the reference state. In addition, the averaged relative entropies of individual residues from all the RRS simulations are plotted in Fig. 12 for Dark and Light states, respectively. Most residues do not show significant entropy change upon rigid residue perturbation. However, certain residues display rather consistent but different trends in RRS simulations of Dark and Light states. The most striking case is residue Lys85, which has significantly positive entropic response in RRS simulations of Dark state and significantly negative entropic response in RRS simulations of Light state. In both Dark and Light states, Lys85 forms strong hydrogen bond with the phosphate group of FAD (Fig. 13). It is notable that N-terminus residues 1 through 10 have generally positive entropic response towards rigid residue simulations in Dark state, and these positive entropic responses are further enhanced in the Light state. On the contrary, the entropic responses from residues 28 through 37 change from positive in Dark state simulations to significant negative in the Light state simulations. The individual residue entropies were also normalized with regard to the atoms number in each residue and illustrated in Figure S1^37^. The similarity between normalized and original residue entropies plots shows that the differences of residue responses are not scaled with residue size, and therefore inherent to protein structure.

### Principal component analysis (PCA)

PCA was carried out for all the simulations to project protein motions onto normal mode vectors. The vectors were used to explore and compare the global motions of VVD in each simulation. Because the main purpose of PCA is characterizing the global motions of the protein, only α carbons were included for this analysis. To uncover the correlation between VVD Dark and Light states dynamics, the optimized Dark and Light structures were used as reference structures for the PCA of unperturbed Light and Dark state simulations, respectively. The accumulative contributions of the first 20 principal components(PCs) of two VVD states are plotted(Figure S2). For both simulations, the PC1s are the dominant vectors with 82% and 92% contribution to the overall protein dynamics, respectively. The remaining contribution to protein dynamics is evenly distributed among other PCs. The dot product between two dominant PC1 vectors is 0.928, showing that both vectors reflect the change between the Dark and Light states. Therefore, PC1 from the Light state trajectory was used for the projection of RRS simulations.

### Projection of RRS simulations onto principal component vectors

To compare the impact of rigid residue perturbations on VVD Dark and Light states distribution in the space of principal components, each pair of RRS simulations were projected onto the PC1 vector from unperturbed Light state simulations(Table S7). The density distribution of each trajectory projected onto each vector was calculated employing kernel density estimator(KDE)^38^. The density distributions for the unperturbed and RRS 112, 119, 124 simulations are shown in Fig. 14.

The unperturbed Dark and Light state simulations are well separated (Fig. 14A). The unperturbed Dark state distribution centering close to the origin(blue line in Fig. 14A) is more diffused than the unperturbed Light state centering close to far left(red line in Fig. 14A). For the RRS112 simulations, the distributions of both Dark and Light state simulations become more diffused and move to each other and with significant overlap. Both distributions of RRS112 simulations display multiple peaks, which correspond to two basins of attractions for both simulations on 2D RMSD plot (Fig. 2B). For the RRS119 simulations, the distribution of Dark state simulation remains close to origin(blue line in Fig. 14C) similar to unperturbed Dark state simulation. This similarity supports the observation that rigid residue 119 exerts very little impact on the VVD Dark state in the simulation (Fig. 3C). However the distribution of RRS119 Light state simulation(red line in Fig. 14C) displays significant shift and overlap with the Dark state. On the contrary, the distribution of RRS124 Light state simulation remains close to the region of far left(red line in Fig. 14D) similar to unperturbed Light state simulation. This similarity supports the observation that rigid residue 124 exerts very little impact on the VVD Light state in the simulation (Fig. 3D). But the distribution of RRS124 Dark state simulation(blue line in Fig. 14D) displays significant shift toward the Light state.

## Discussion

In the design of optogenetic tools, a fundamental limitation of LOV-based systems is the lack of a digital switch from a non-functional off-state to a highly-function on-state. Rather, optogenetic tools have residual activity in both the Light and Dark states thereby limiting their fidelity. To examine the origins of the lack of a digital LOV-based photoswitch, we wished to computationally examine the allosteric activity of LOV based proteins to extract information relevant to the conformational landscape and configurational entropy of Dark and Light states of VVD. In so doing, we discovered an inherent difference in correlating sampling of conformational space with calculations of configurational entropy. Specifically, where the VVD Dark state samples more conformational space, but the Light state has higher configurational entropy. These analyses shed light on the nature of allostery in LOV based photoswitches.

**Figure 1:**
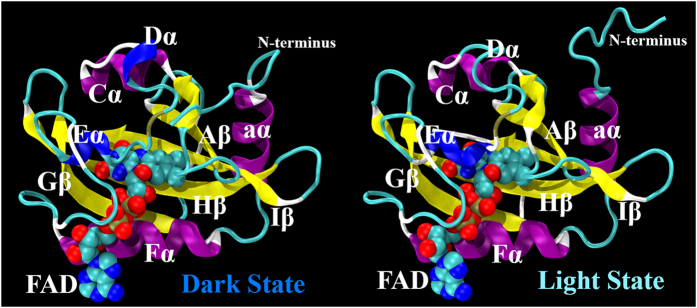
Dark(PDB code: 2PD7) and Light(PDB code: 3RH8) Structures of VVD.

Examination of the unperturbed 210 ns simulations revealed profound differences between the overall dynamics of VVD Dark and Light states. Specifically, the Dark state samples more conformational space during the course of 210 ns simulations than the Light state (Fig. 1B). Further, the Dark state samples conformational space, which lie close to the Light state, thereby likely contributing to residual activity in both states and loss of a digital switch as observed in optogenetic tools. The counterintuitive nature of the conformational sampling of the Dark state, could lie in the need to facilitate a fast switch from the dormant Dark state to active Light state and to reduce barriers in the transition. In such a system, the Dark state samples conformational space poised for activity. Blue-light then populates the Light state that is “locked” into specific conformational space to better serve its signaling function.

MD simulations provide direct time series protein dynamical data. Conformational landscape is a comprehensive representation of intrinsic protein dynamics. The configurational entropy calculated based on MD simulations is a quantitative metric to measure the distribution of given protein on its conformational landscape. Because it is preferred to calculate configurational entropy around single attraction basin when using quasi-harmonic approximation^39^, the first 30 ns trajectories of the unperturbed simulations, during which two states of VVD protein remain separate and close to their native crystallographic structures, were used to compute the configurational entropies for the sampling space. In addition, 30 ns trajectory was carried out for each RRS simulation to be consistent with unperturbed simulations. Our previous study also support that the 30 ns trajectories are sufficient to probe protein allosteric mechanisms^34^.

**Figure 2:**
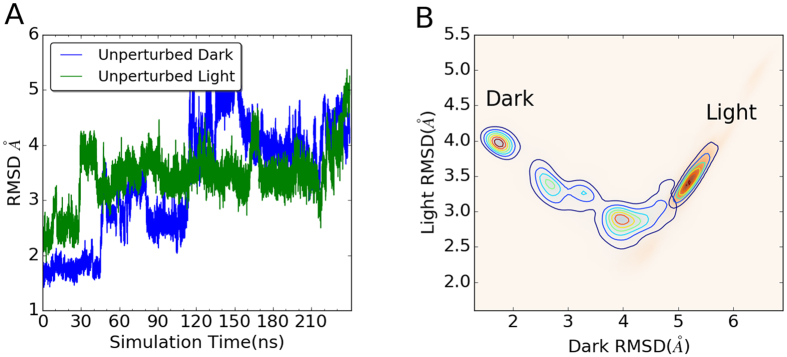
(**A**) RMSD plots for the unperturbed Dark and Light MD simulations.(**B**) Two dimensional(2D) RMSD contour plot of the unperturbed Dark and Light MD simulations using RMSD values with references to both optimized Dark and Light states.

**Figure 3:**
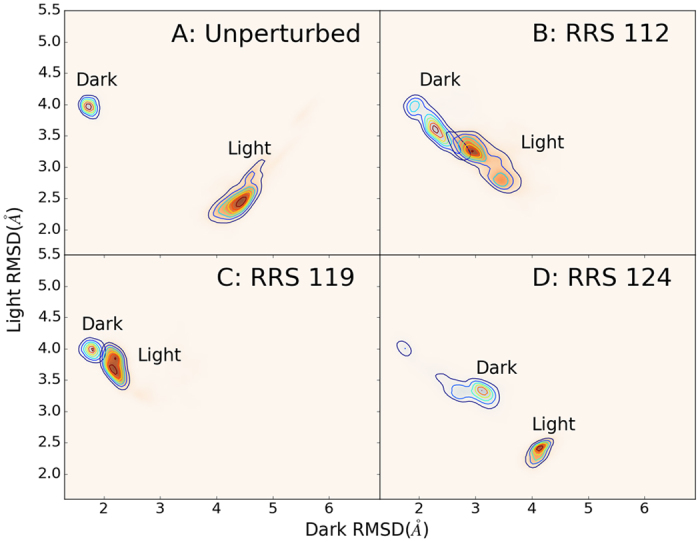
2D contour plots of Dark and Light states distributions. (**A**) Unperturbed simulations(30 ns to be consistent with RRS simulations);(**B**) RRS 112;(**C**) RRS 119;(**D**) RRS 124. RMSD values for X and Y axes are calculated with reference to the optimized Dark and Light structures, respectively.

**Figure 4:**
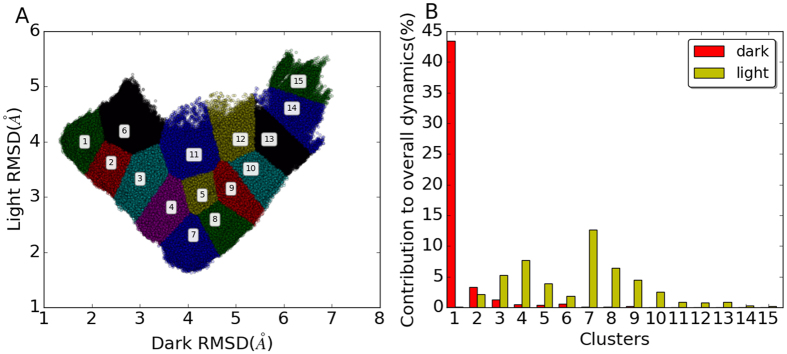
(**A**) Cluster analysis of 2D RMSD distribution of all simulations combined;(**B**) Distribution of all simulations among 15 clusters.

**Figure 5:**
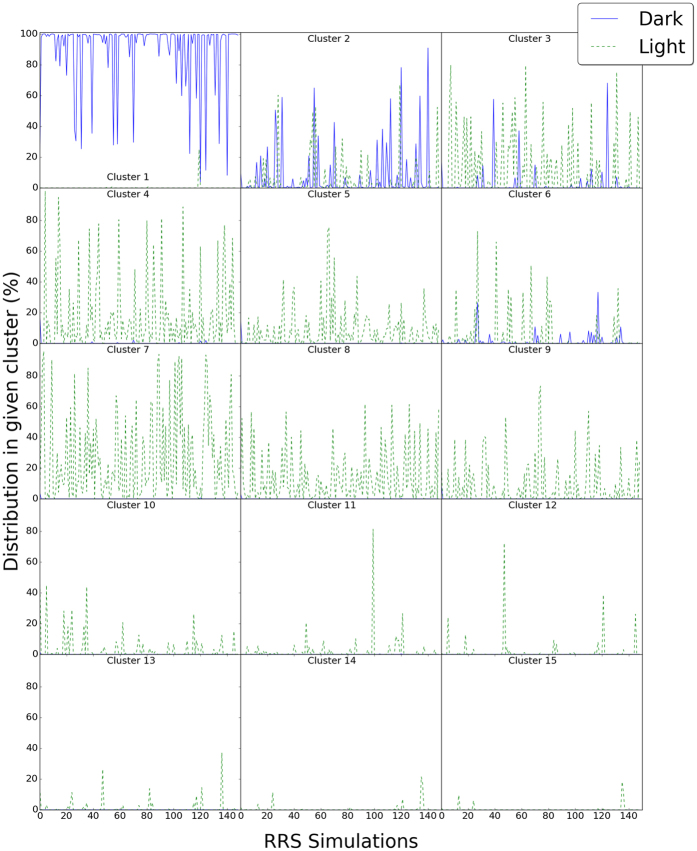
Distribution of individual RRS simulations in each of 15 clusters.

The 2D RMSD plots of RRS simulations provide detailed information with regard to the comparison of impacts from rigid residue perturbation on Dark and Light state dynamics. The different impact on the VVD dynamics from the perturbation on residues 112, 119, and 124 could provide mutagenesis targets for different purposes, i.e. when different changes are desired on the Dark and Light states, or to improve the digital-nature of optogenetic tools. Interestingly, there are many more residues(more than 30) upon which the rigid body perturbation significantly affects the Light state(Table 1), than those affect either Dark state only(four residues) or both states(three residues). These disproportional numbers of residues in different groups demonstrate the potential value of this analysis as the guidance for further experimental study. These also suggest that the Light state is much more prone to perturbations than the Dark state, and may correlate with the narrower distribution of the Light state simulation on the 2D RMSD plot (Fig. 2B).

One interesting observation is that the side chains of selected key residues 112, 119, and 124 are located at the protein surface and point away from the binding site of flavin (Fig. 15). This certainly should not be considered as purely coincidental. These three residues are identified through their dramatic impact on overall protein dynamics. Therefore it is logical to hypothesize that these residues could carry allosteric function to propagate the signal triggered by light induced activation of VVD.

The clustering analysis of the distribution of all RRS and unperturbed simulations on 2D RMSD plots provides a comprehensive representation of the conformational space accessible to the VVD Dark and Light states. The functional conformations and structures of both Dark and Light states as well as potential intermediates all belong to this conformational space. In a previous crystallographic study of VVD mutant Cys71Val, two distinctive structures were identified for its Light state (Fig. 16. 3D72_A, blue, and 3D72_B, ice blue)^31^. The extended N-terminus in the Cys71Val mutation was also observed through small-angle X-ray scattering(SAXS) studies^25^,^40^. Correspondingly, representative conformations of Clusters 1(red) and 13(magenta)(Fig. 16) are similar to these two structures. The native Light state displays yet another conformation of N-terminus(3RH8, green structure in Fig. 16). It was also stated that electron density of residues 37–44 was weak or absent in the crystallographic data of VVD Cys71Val mutant in Light state^31^. The wide distribution of this region represented by all 15 clusters (Fig. 2B) also showed that this region is very flexible with access to large conformational space.

**Figure 6:**
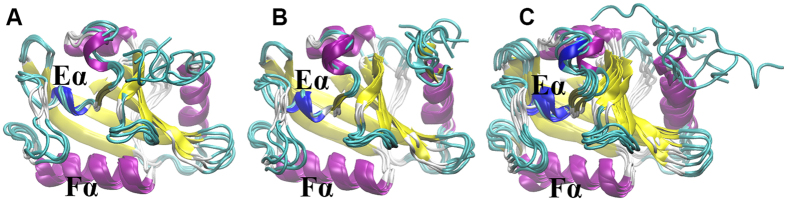
Representative structures for 15 clusters. (**A**) Clusters 1, 2, 3, 6;(**B**) Clusters 4, 5, 7, 8, 9;(**C**) Clusters 10, 11, 12, 13, 14, 15.

**Figure 7:**
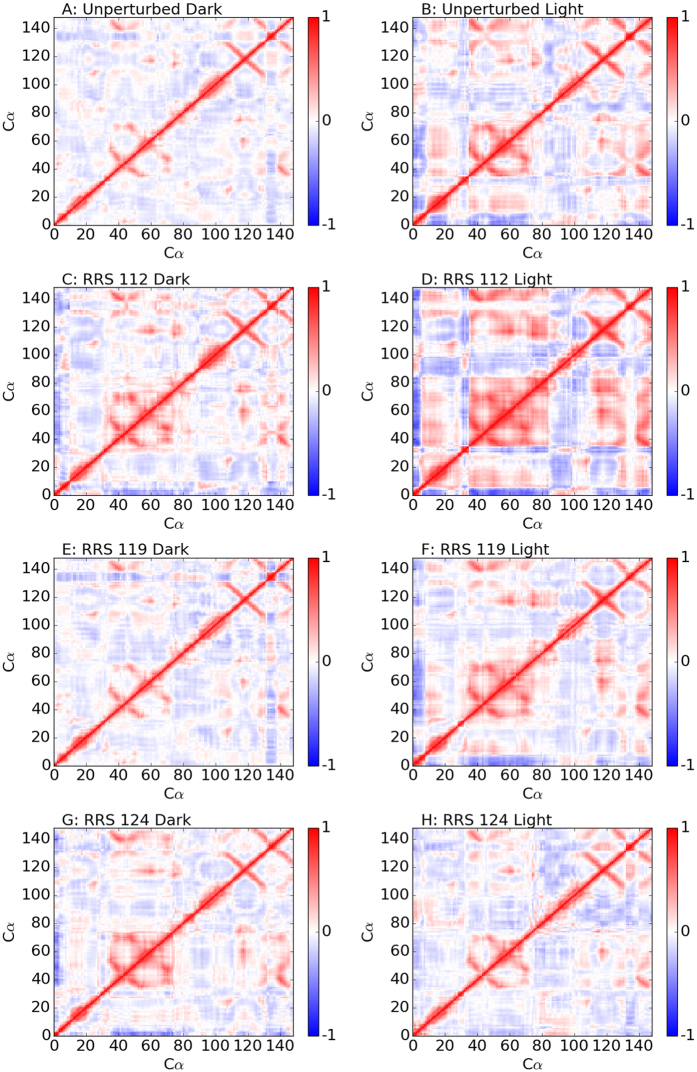
Cross-correlation matrix heat maps for unperturbed, RRS 112(Glu), RRS 119(Asn), RRS 124(Val) MD simulations of Dark and Light states.

**Figure 8:**
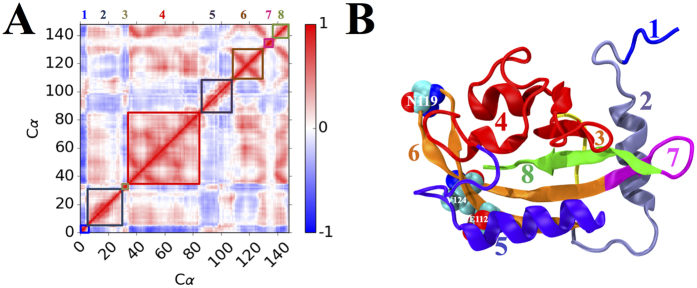
Structural regions revealed by cross-correlation analysis. (**A**) Regions highlighted in matrix of RRS 112 of Light state.(**B**) Regions illustrated in crystallographic structure of Light state. Listed regions: Region number, residues(color): 1, 1−5(Blue); 2, 6−30(ice blue); 3: 31−34(yellow); 4: 35−83(red); 5: 84−108(violet); 6: 109−131(brown); 7: 132–138(magenta); 8: 139−148(green). Residues E112, N119, and V124 are also labeled.

**Figure 9:**
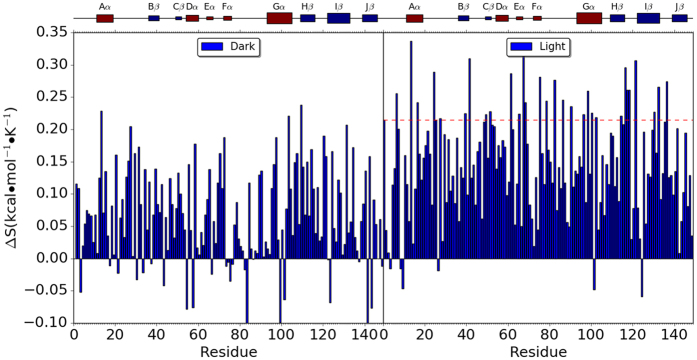
Relative entropy of VVD in dark and light states from RRS simulations. The entropy from unperturbed dark simulation is used as reference. Red horizontal line in Light state plot marks the entropy level from the unperturbed Light state simulation.

**Figure 10:**
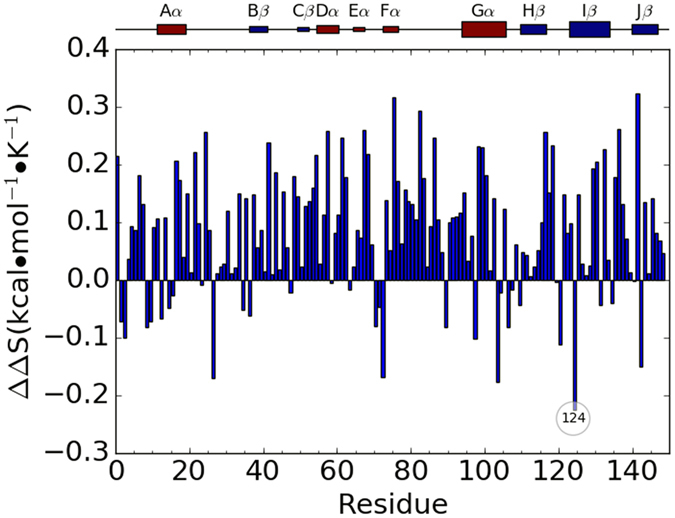
Entropy difference between dark and light VVD states from unperturbed simulation and RRS simulations.

**Figure 11:**
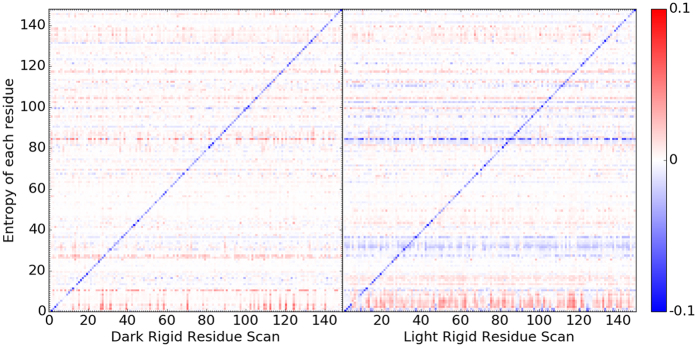
Heat map of individual residue entropy contribution under rigid residue perturbation for Dark(left) and Light(right) states. The horizontal axis corresponds to the RRS simulations and indices of residues being held as rigid body. The vertical axis corresponds to the residue index for entropy contributions. The entropy contribution from each residue in unperturbed simulation is set as reference.

**Figure 12:**
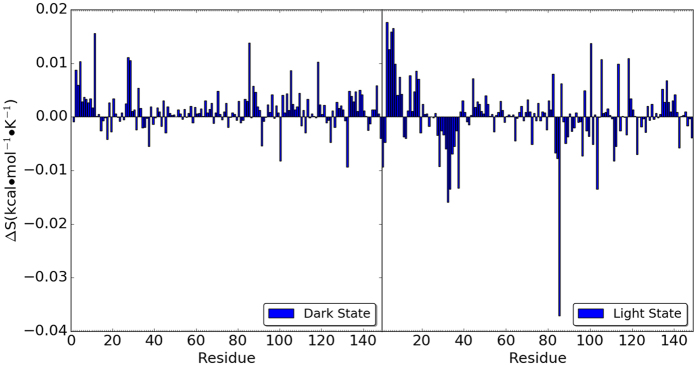
Average entropic response from each simulation in all RRS simulation.

**Figure 13:**
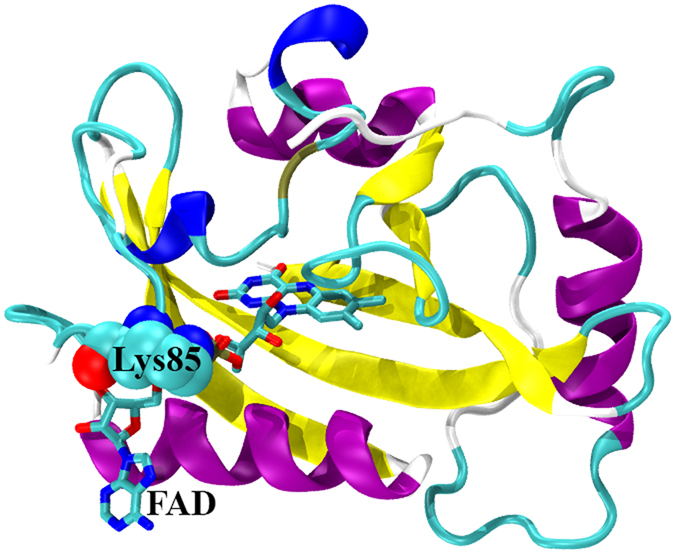
VVD Dark state with Lys85(shown as sphere mode) and FAD(shown as stick mode).

**Figure 14:**
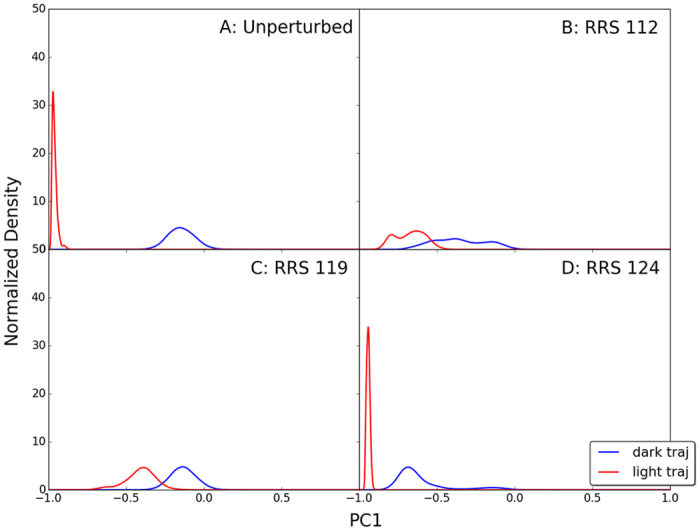
The distributions of unperturbed and RRS 112, 119, 124 simulations onto Principal Component 1 vector from unperturbed Light state simulations using optimized Dark state structure as reference.

**Figure 15:**
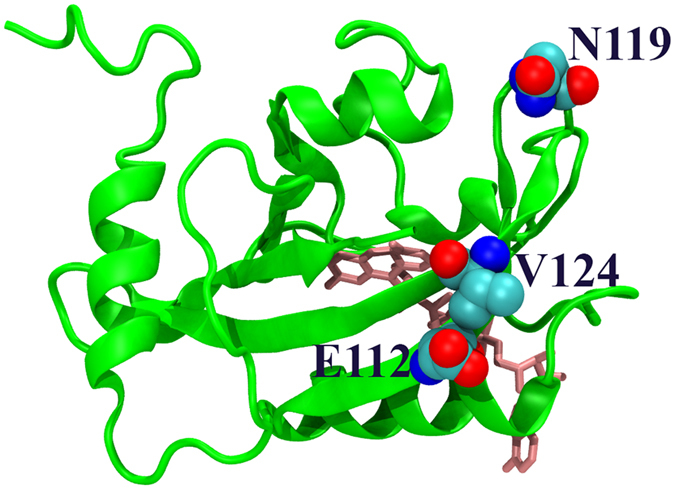
Side chains of key residues E112, N119, and V124. VVD protein is shown in green. Ligand flavin monocleotide is shown in brown.

**Figure 16:**
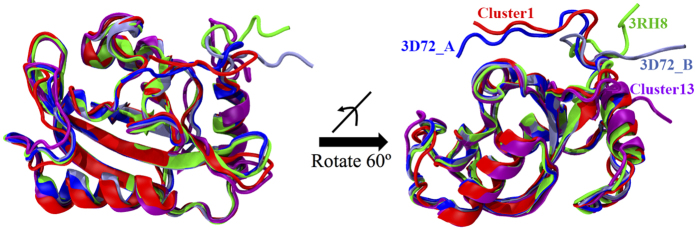
Various conformations identified for VVD: Cluster 1(red) and 3D72_A(blue) Cluster 13(magenta), 3D72_B(ice blue) and 3RH8(green).

Another intriguing observation of clustering analysis is that in clusters 1 through 10, the Fα and Eα helices, which cradle the FAD cofactor, remain in an extremely similar orientation, leaving a narrow space in between (Fig. 6A and B). However, in clusters 11 through 15, both Fα and Eα helices display obvious deviations among these structures, creating more space probably responding to the photo-activation of FAD.

**Figure 17:**
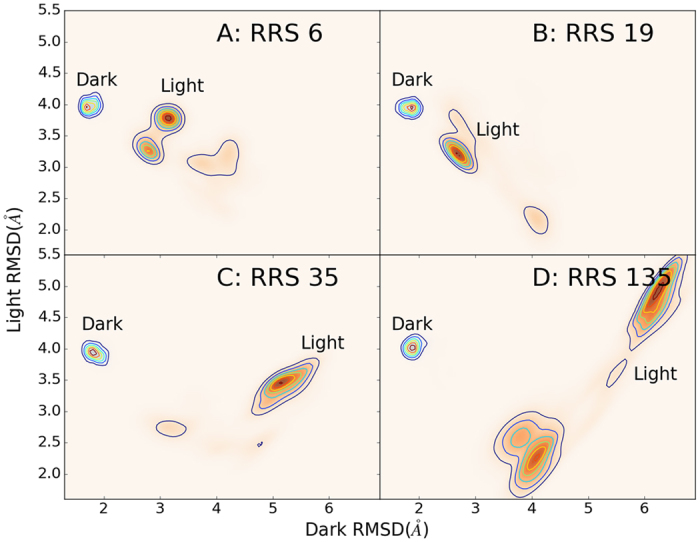
2D contour plots of Dark and Light states distributions for RRS 6(Pro), RRS 19(Met), RRS 35(Cys), and RRS 135(Glu) MD simulations of Dark and Light states. RMSD values for X and Y axes are calculated with reference to the optimized Dark and Light structures, respectively.

**Figure 18:**
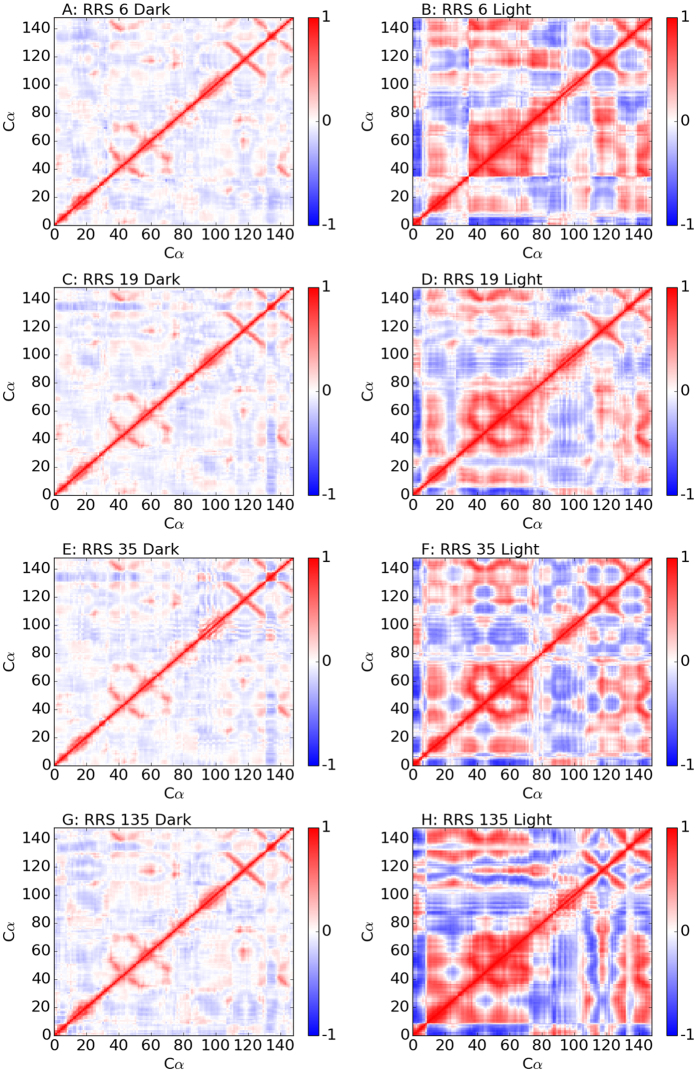
Cross-correlation matrix heat maps for RRS 6(Pro), RRS 19(Met), RRS 35(Cys), and RRS 135(Glu) MD simulations of Dark and Light states.

Both Pro42 and Cys71(residue number 6 and 35 in the current study, respectively) are key residues projecting the N-terminus into different conformations^24^,^31^. Correspondingly, both RRS6 and RRS35 simulations showed significant impact on VVD Light state dynamics (Fig. 17). In RRS6, the Light state distribution splits into two attraction basins. In RRS35, the Light state distribution shifts upward along Y-axis, farther away from crystallographic Light state conformation. The other two key residues 55 and 171(residue number 19 and 135 in the current study, respectively) for VVD dimerization process discussed in ref. 31 also have significant impact on Light state dynamics under constraint perturbation (Fig. 17). In RRS19, the Light state distribution shifts toward left hand side along X-axis, closer to crystallographic Dark state conformation. But in RRS135, the Light state distribution splits into two attraction basins, which are significantly separate from each other. For all four residues above, the cross-correlations are significantly enhanced in Light states, while the cross-correlations remains moderate similar to unperturbed Dark state (Fig. 18).

It is intriguing that rigid residue perturbations on different residues have drastically different impact on the overall distributions of VVD Dark and Light state simulations. The systematic probe of all residues through RRS simulations provides a comprehensive map of impact for every single residue on overall protein dynamics, which could serve as guidance to identify potential target for improving the fidelity of optogenetic tools. The fact that very few residues could serve as targets to affect Dark state dynamics signifies the potential usefulness of RRS as systematic probing tool to identify allosteric sites with specific effects.

As demonstrated in this research, the RRS method could be employed as an effective computational tool to systematically perturb the protein structure through molecular dynamics simulations to screen key residues potentially important for protein allostery. This method has several advantages. First, no *a priori* knowledge about the target protein is necessary, which makes it easy to implement. Second, no mutations are generated to screen residues, because rigid body constraints are used as perturbation instead of mutations. Therefore, the chemical nature of molecular interaction between each target residue and its local environment is reserved, and the main effect being observed from the simulation would be impact on overall protein dynamics from removing the internal degrees of freedom of target residue. Because no mutations are necessary, the difficulty of choosing somewhat arbitrary amino acids for mutation could be avoided. Third, extensive molecular simulations could probe the change of real protein dynamics, and are more reliable than approximation methods such as elastic network models. Fourth, the scan provides complete information of all residues. Therefore any previously unnoticed but potentially important residues could be identified for further investigation. This method does have certain limitations. For example, the rigid body constraint is an unphysical perturbation. Therefore, the observation from using this method could not be rigorously verified by experiments. However, this could be overcome by additional simulations of mutants on the key residues selected using RRS method. In addition, the computational cost of proteins containing large number of residues could be prohibitively expensive for long molecular dynamics simulations. This could be overcome by running relatively short simulations as prescreening to select residues for longer simulations. Considering all the factors above, the RRS method is an effective and affordable computational tool, could be valuable and compatible with other methods for many systematic and comprehensive studies of protein structures and functions.

## Materials and Methods

### Molecular dynamics simulations

The initial structures for Dark and Light states of VVD were obtained from the Protein Data Bank(PDB)^41^ with IDs as 2PD7 and 3RH8, respectively. The Dark state in 2PD7 contains residues 36 through 184, and the Light state in 3RH8 contains residues 37 through 184. For consistency and convenience, residue 36 in 2PD7 was removed and residues 37 through 184 in both states were numbered as residues 1 through 148. Both structures include protein and ligand flavin adenine dinucleotide(FAD). Because flavin monocleotide(FMN) and FAD carry similar biological role, adenosine monophosphate(AMP) moiety was removed from FAD to form FMN, which was parameterized with force field from a previous study^29^. Hydrogen atoms were added to the VVD Dark and Light states, which were subsequently solvated using explicit water model(TIP3P)^42^ and neutralized and balanced by sodium cations and chloride anions. The ionic strength of simulation box is 0.11 M for both Dark and Light states. The simulation systems were subjected to energy minimization with 200 steepest descent steps(for both), 1709 adopted basis Newton-Raphson(ABNR) minimization steps for Dark state and 1540ABNR steps for Light state, yielding a total gradient of less than 0.03 kcal/mol/Å. The optimized simulation boxes were subjected to 12 picoseconds(ps) equilibrium simulations raising the temperature from 100 K to 300 K. 10 nanoseconds(ns) of isothermal-isobaric ensemble(NPT) MD simulations were carried out for both states. Appropriate simulation boxes with size closest to the average NPT simulation boxes were selected for two VVD states for production canonical ensemble(NVT) Langevin MD simulations at 300 K.

We applied rigid residue scan(RRS) method developed in our group^33^,^34^ to systematically detect key allosteric residues related to Dark and Light states. In RRS method, rigid body constraint using an efficient integrator^32^ was applied on each individual residue in separate simulations(referred as RRS simulations). Therefore, there are 148 rigid residue simulations for each state. The simulations without rigid residue constraints were referred as unperturbed simulations. For both unperturbed and RRS simulations, initial 4 ns NVT simulations were considered as equilibrium stage and not counted as production run. Besides these 4 ns equilibration, total of 210 ns unperturbed MD simulations were carried out for Dark and Light state, respectively, and total of 30 ns RRS MD simulations were carried out for each residue in each state. Overall we performed 9,300 ns production NVT simulations.

For all simulations, bonds associated with hydrogen atoms were constrained, and step size of 2 femtosecond(fs) was used. Simulation trajectories were saved every 500 MD steps. Cubic simulation box and periodic boundary condition were applied for all MD simulations. Electrostatic interactions were calculated using particle mesh Ewald(PME) method^43^. All simulations were carried out using CHARMM simulation package^44^ version 39b1 and the CHARMM22 force field with CMAP correction^45^,^46^.

### Analysis of MD trajectory

#### Least-square-fitting alignment root-mean-square deviation(RMSD)

The minimized structures for the Dark and Light states were used as reference structures for RMSD analyses, which is defined as:


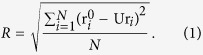


*N* is the number of atoms, 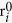 is the Cartesian coordinate vector for atom *I*, and U is the best-fit alignment transformation matrix between a given structure and its reference structure. For every trajectory, RMSD values were calculated using both Dark and Light optimized structures as references. Two RMSD values were used to plot 2D RMSD distribution.

#### Cross-correlation(normalized covariance) matrix

The cross-correlation matrix was calculated to detect the correlation among atomic motions of protein. The matrix element C_ij_ measuring correlation between atoms i and j is defined as


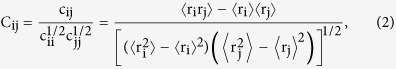


where c_ij_, c_ii_, and c_jj_ are the atomic coordinate covariance matrix elements, and r_i_ and r_j_ are Cartesian coordinate vectors from the least-square fitted structures. The translational and rotational motions were projected out through the least-square fitting procedures. The normalized matrix elements range from -1 to 1, with positive value indicating positive correlation and negative values indicating negative correlations.

#### Quasi-harmonic analysis and configurational entropy calculation

Quasi-harmonic analysis was carried out through the inversion of cross-correlation matrix C


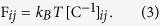


**F** is the force constant matrix for the effective quasi-harmonic potential^47^, *k*_*B*_ is the Boltzmann constant, and *T* is the temperature. The normal modes and corresponding frequency ω of the molecule on the effective quasi-harmonic potential can be calculated through the solution of secular equation


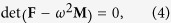


where **M** is the mass matrix of protein. Subsequently, protein configurational entropy *S*_*config*_ within harmonic limit was calculated using the frequency from above quasi-harmonic analysis,


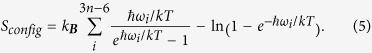


The configurational entropy for each individual residue could be calculated in the similar way using sub-correlation-matrix for atoms within the same residue(including side chain and backbone). The configurational entropy for each individual residue calculated in this way does not include the correlation between the target residue and the rest of the system. Therefore, the sum of individual residue configurational entropies is not expected to match with the one of whole protein.

### Principal component analysis (PCA)

PCA was carried out for each simulation to examine overall protein motions. The only difference between PCA and quasi-harmonic analysis is that PCA does not use mass weighting. Because the focus of PCA is overall protein motions, only protein α carbons from each trajectory were used for this purpose. It was occurred to us that the different choices of reference structures could reveal different prospects of protein dynamics. Therefore, both optimized Dark and Light structures were used as reference structures for PCA of each simulation. For each RRS simulation, the residue being held rigid was included in the PCA just as other residues. All the analyses described above were carried out using CHARMM version 39b1.

## Additional Information

**How to cite this article:** Zhou, H. *et al*. Revealing Hidden Conformational Space of LOV Protein VIVID Through Rigid Residue Scan Simulations. *Sci. Rep.* **7**, 46626; doi: 10.1038/srep46626(2017).

**Publisher's note:** Springer Nature remains neutral with regard to jurisdictional claims in published maps and institutional affiliations.

## Supplementary Material

Supplementary Information

## Figures and Tables

**Table 1:** Selected RRS simulations with significant effect on the 2D RMSD distributions for either Dark or Light states or both.

Affecting:	mainly Dark state	mainly Light state	both states
RRS simulations	39, 58, 70, 124	6, 11, 16, 17, 19, 25, 28, 30, 41, 46, 50–54, 56, 61, 62, 67, 75, 76, 79, 81, 82, 86, 90, 95, 98, 102, 116, 118, 119, 129, 131, 132, 135, 141	27, 55, 112

**Table 2:** Distribution of selected individual Dark and Light state simulations among 15 clusters.

Clusters	Unperturbed		RRS112		RRS119		RRS124	
	Dark%	Light%	Dark%	Light%	Dark%	Light%	Dark%	Light%
1	**18**.**9**	0.0	**22**.**3**	0.0	**99**.**8**	**25**.**1**	**11**.**4**	0.0
2	**10**.**3**	0.0	**58**.**0**	5.4	0.2	**67**.**0**	**18**.**6**	0.0
3	**18**.**3**	0.0	**12**.**4**	**55**.**4**	0.0	5.7	**68**.**1**	0.0
4	**18**.**3**	0.0	0.1	**35**.**1**	0.0	0.2	1.9	0.0
5	**15**.**7**	0.2	0.0	1.5	0.0	0.0	0.0	0.0
6	0.2	0.0	7.3	0.0	0.0	1.0	0.0	0.0
7	3.9	5.0	0.0	0.9	0.0	0.0	0.0	**93**.**5**
8	3.2	6.1	0.0	0.2	0.0	0.0	0.0	**6**.**5**
9	**9**.**1**	**20**.**8**	0.0	0.0	0.0	0.0	0.0	0.0
10	1.4	**49**.**6**	0.0	0.0	0.0	0.0	0.0	0.0
11	0.1	0.0	0.0	1.5	0.0	1.1	0.0	0.0
12	0.6	0.7	0.0	0.0	0.0	0.0	0.0	0.0
13	0.0	**14**.**5**	0.0	0.0	0.0	0.0	0.0	0.0
14	0.0	1.8	0.0	0.0	0.0	0.0	0.0	0.0
15	0.0	1.2	0.0	0.0	0.0	0.0	0.0	0.0

(Significant distributions are listed in bold).
