# Supplementary material for: Revealing Hidden Conformational Space of LOV Protein VIVID Through Rigid Residue Scan Simulations
Source: Sci Rep. 2017 Apr 20;7:46626. doi: 10.1038/srep46626 (PMC5397860; doi:10.1038/srep46626)
Supplement: Supplementary Information [file srep46626-s1.pdf]

Revealing Hidden Conformational Space of LOV Protein VIVID Through Rigid Residue Scan Simulations

Supplementary Data

*Hongyu Zhou, Brian D. Zoltowski, and Peng Tao**

Department of Chemistry, Center for Drug Discovery, Design, and Delivery (CD4), Center for Scientific Computation, Southern Methodist University, Dallas, Texas 75275, United States of America

* Corresponding authors

Peng Tao: Telephone: 1-214-768-8802, Fax: 1-214-768-4089, Email: ptao@smu.edu

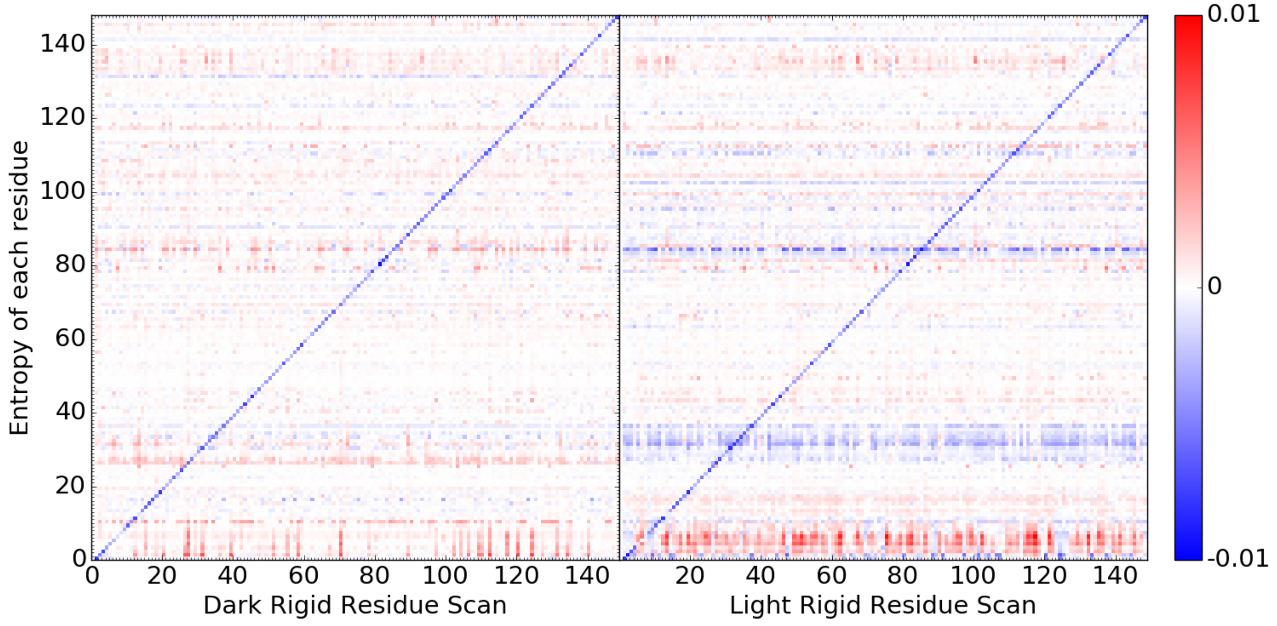

Fig. S1. Heat map of individual residue entropy contribution normalized based on the number of atoms in each residue under rigid residue perturbation for dark (left) and light (right) states. The horizontal axis corresponds to the RRS simulations and indices of residues being held as rigid body. The vertical axis corresponds to the residue index for entropy contributions. The entropy contribution from each residue in unperturbed simulation is set as reference.

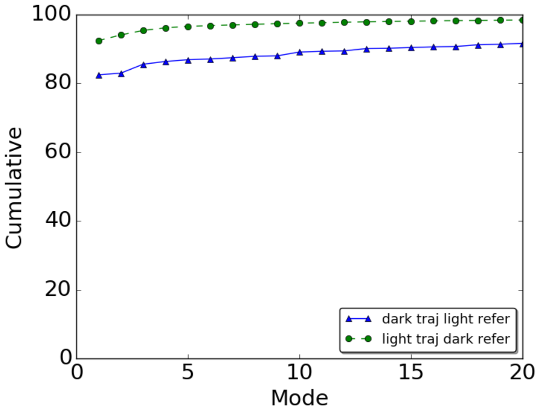

Fig. S2: Distribution from unperturbed Dark and Light state simulations to two 20 principal components (PCs) generated from the unperturbed Dark state simulation with optimized Light structure as reference.

Table S1: RMSD plots of VVD from rigid residue scan for both Dark and Light states. Each residue number represents a simulation in which that particular residue is subjected to rigid body constraints.

Residue	Dark	Light	Residue	Dark	Light
None	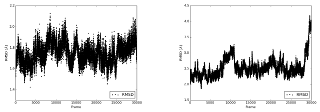				
1	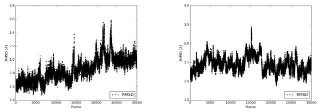		2	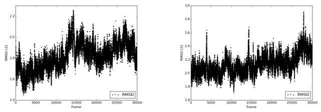	
3	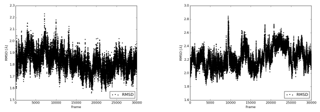		4	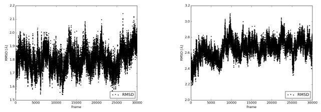	
5	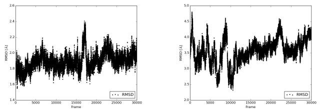		6	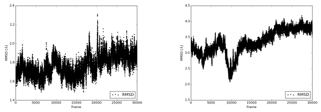	
7	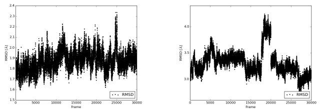		8	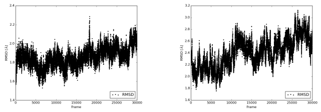	
9	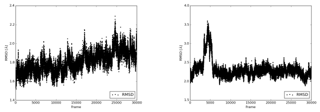		10	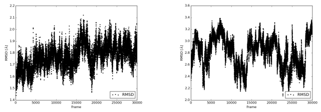	
11	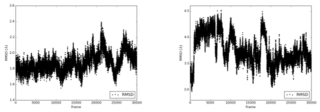		12	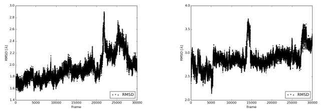	
13	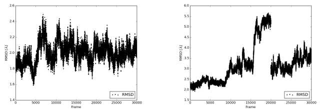		14	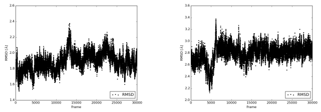	
15	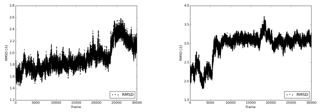		16	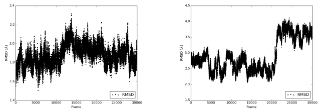	
17	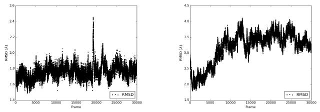		18	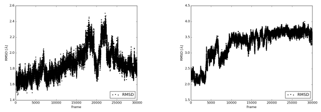	
19	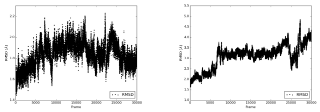		20	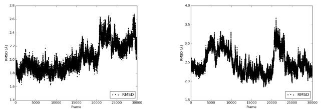	
21	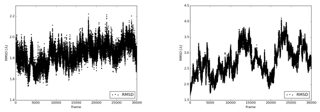		22	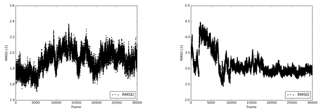	

Table S1: RMSD plots of VVD from rigid residue scan for both Dark and Light states. Each residue number represents a simulation in which that particular residue is subjected to rigid body constraints.

Residue	Dark	Light	Residue	Dark	Light
23	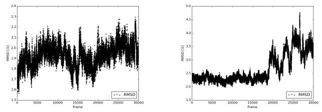		24	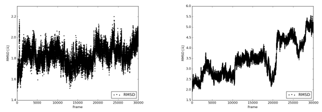	
25	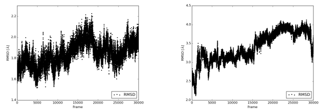		26	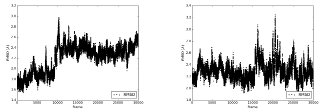	
27	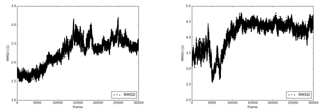		28	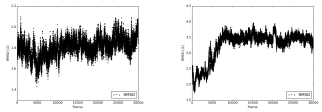	
29	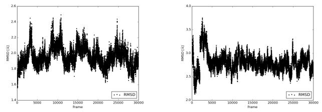		30	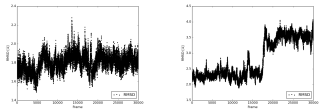	
31	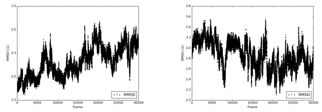		32	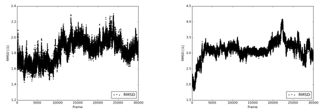	
33	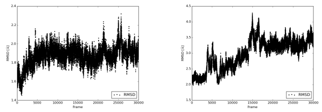		34	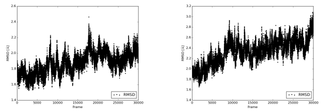	
35	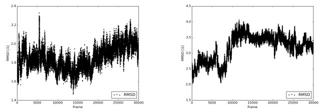		36	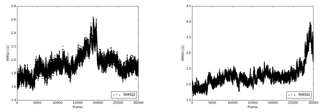	
37	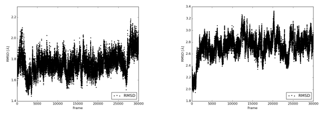		38	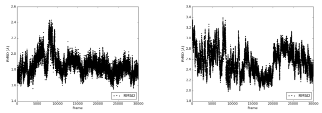	
39	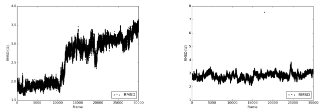		40	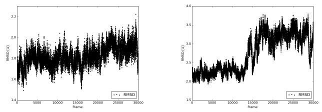	
41	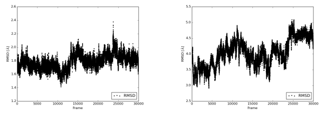		42	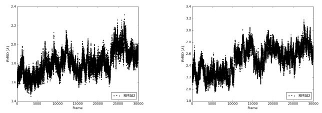	
43	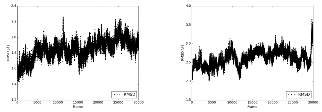		44	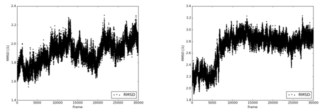	
45	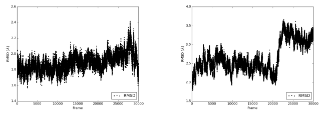		46	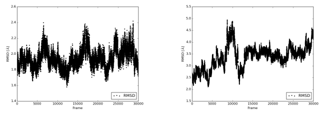	

Table S1: RMSD plots of VVD from rigid residue scan for both Dark and Light states. Each residue number represents a simulation in which that particular residue is subjected to rigid body constraints.

Residue	Dark	Light	Residue	Dark	Light
47	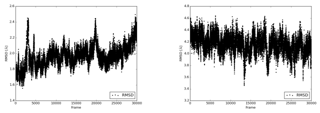		48	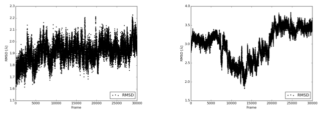	
49	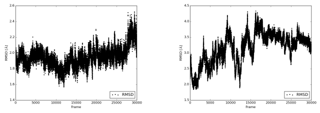		50	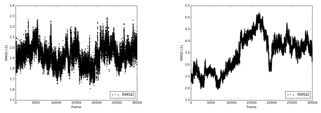	
51	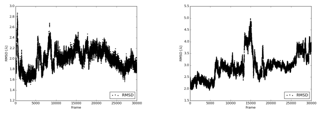		52	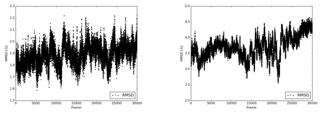	
53	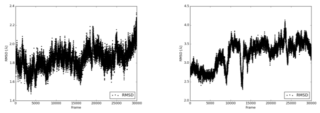		54	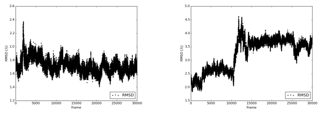	
55	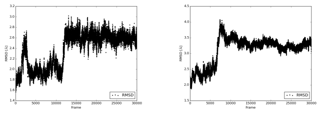		56	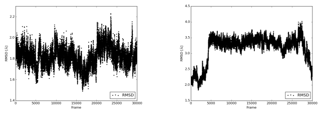	
57	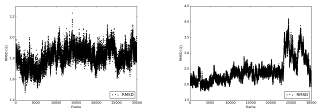		58	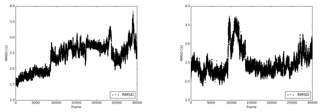	
59	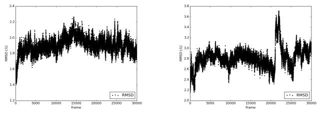		60	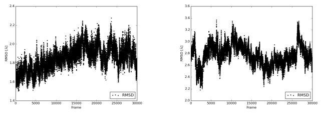	
61	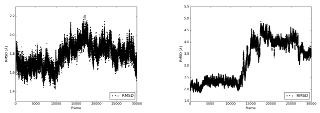		62	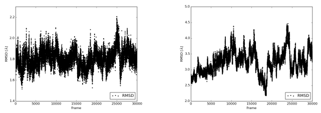	
63	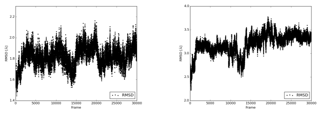		64	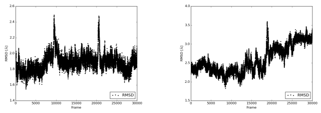	
65	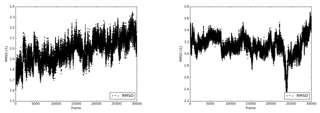		66	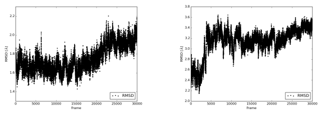	
67	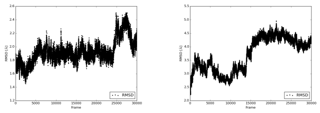		68	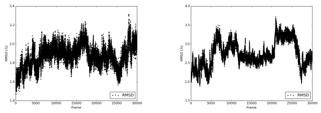	
69	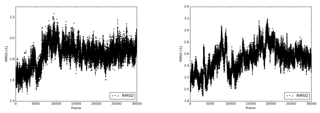		70	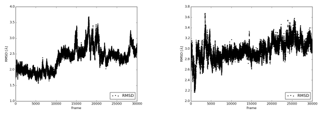	

Table S1: RMSD plots of VVD from rigid residue scan for both Dark and Light states. Each residue number represents a simulation in which that particular residue is subjected to rigid body constraints.

Residue	Dark	Light	Residue	Dark	Light
71	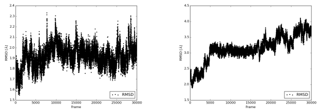		72	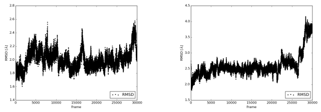	
73	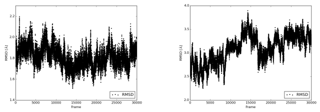		74	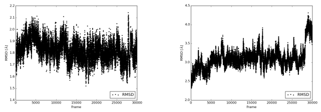	
75	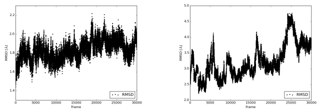		76	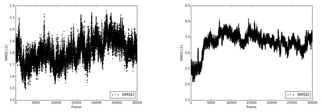	
77	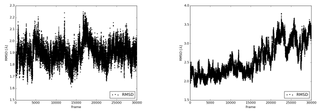		78	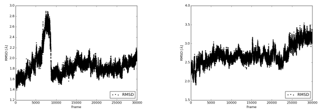	
79	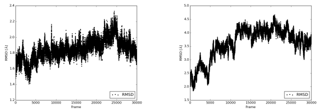		80	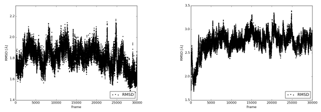	
81	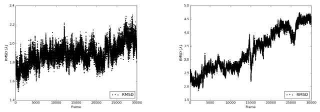		82	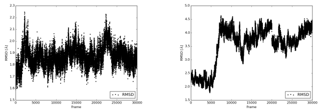	
83	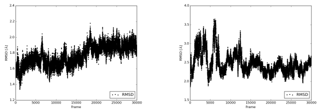		84	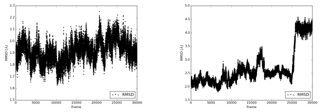	
85	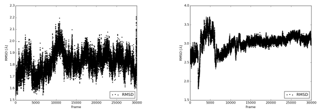		86	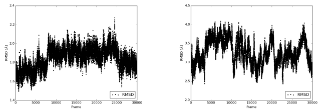	
87	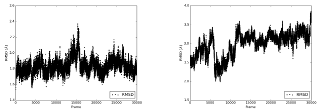		88	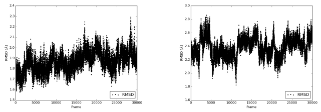	
89	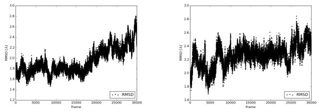		90	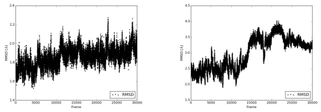	
91	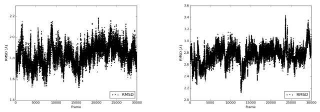		92	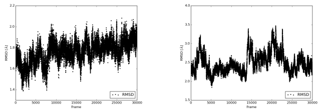	
93	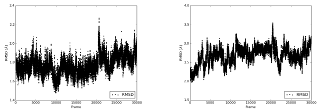		94	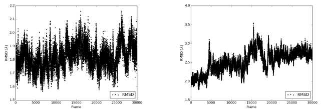	

Table S1: RMSD plots of VVD from rigid residue scan for both Dark and Light states. Each residue number represents a simulation in which that particular residue is subjected to rigid body constraints.

Residue	Dark	Light	Residue	Dark	Light
95	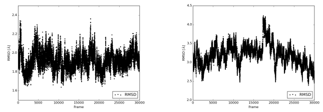		96	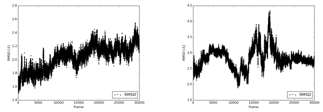	
97	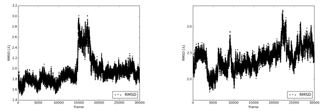		98	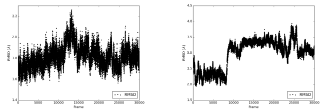	
99	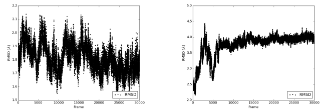		100	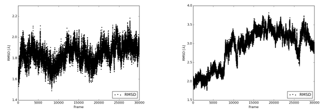	
101	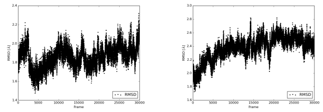		102	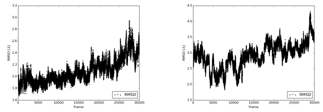	
103	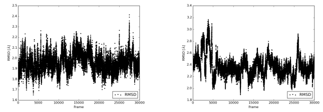		104	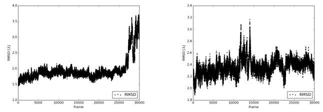	
105	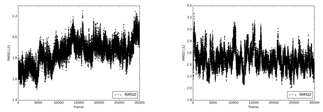		106	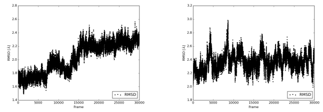	
107	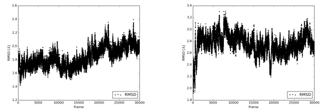		108	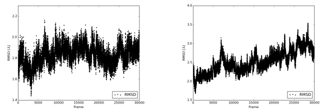	
109	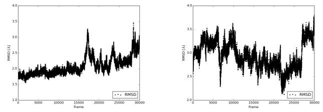		110	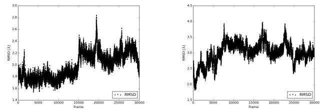	
111	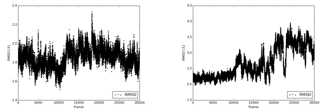		112	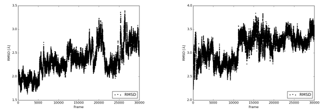	
113	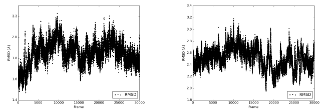		114	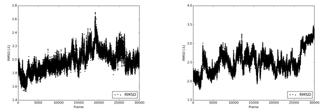	
115	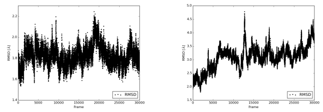		116	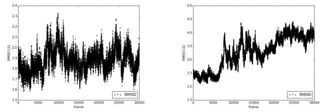	
117	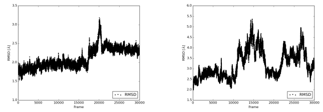		118	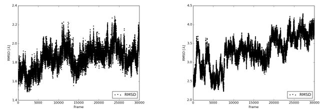	

Table S1: RMSD plots of VVD from rigid residue scan for both Dark and Light states. Each residue number represents a simulation in which that particular residue is subjected to rigid body constraints.

Residue	Dark	Light	Residue	Dark	Light
119	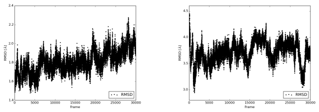		120	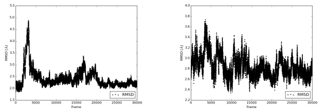	
121	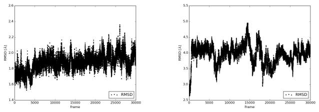		122	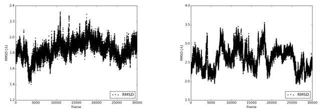	
123	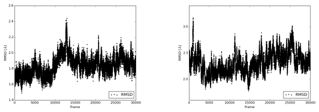		124	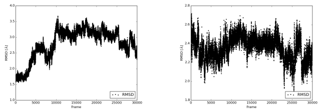	
125	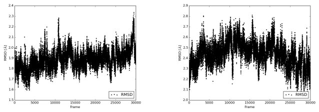		126	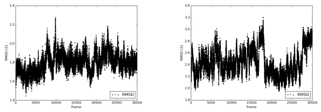	
127	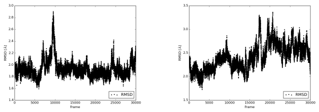		128	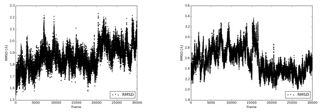	
129	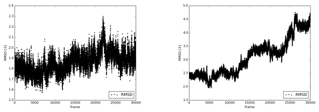		130	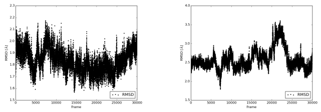	
131	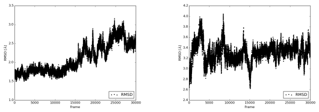		132	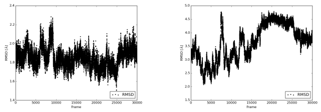	
133	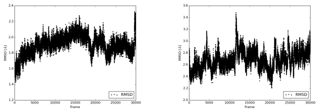		134	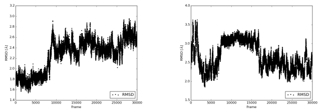	
135	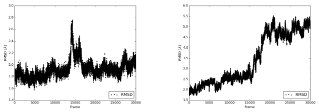		136	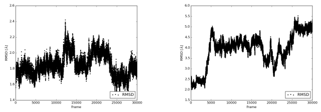	
137	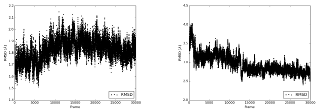		138	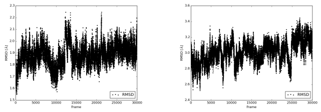	
139	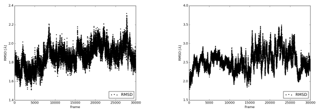		140	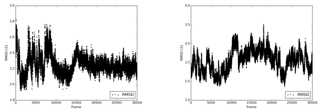	
141	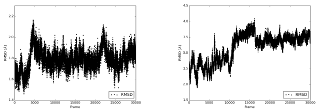		142	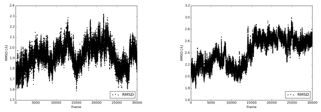	

Table S1: RMSD plots of VVD from rigid residue scan for both Dark and Light states. Each residue number represents a simulation in which that particular residue is subjected to rigid body constraints.

Residue	Dark	Light	Residue	Dark	Light
143	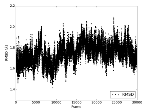	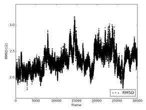	144	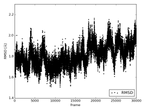	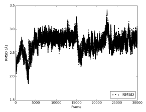
145	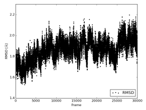	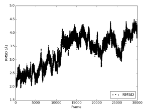	146	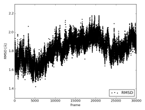	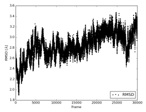
147	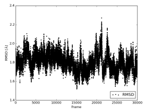	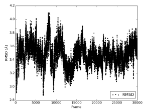	148	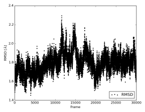	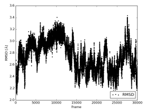

Table S2: Two dimensional (2D) RMSD contour plots of rigid residue scan (RRS) VVD Dark and Light MD simulations using RMSD values with references to both optimized Dark and Light states. Each residue number represents a simulation in which that particular residue is subjected to rigid body constraints. In each plot, Dark state attraction basin is illustrated mainly in green, and Light state attraction basin is illustrated mainly in brown.

Residue	Total Density	Residue	Total Density
None	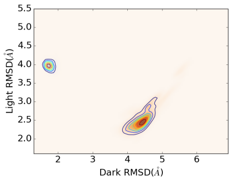		
1	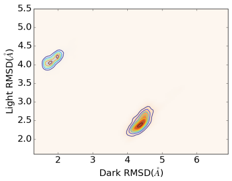	2	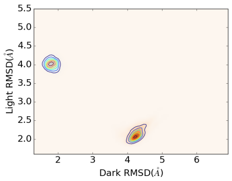
3	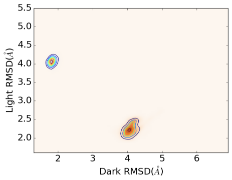	4	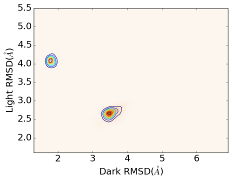
5	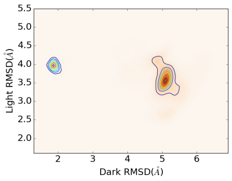	6	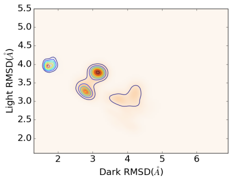
7	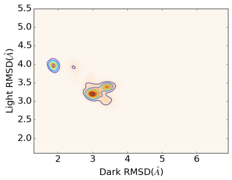	8	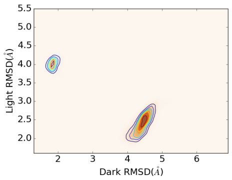
9	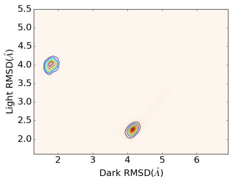	10	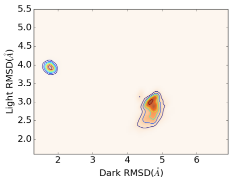
11	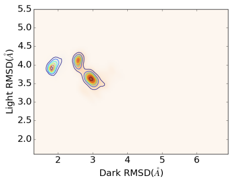	12	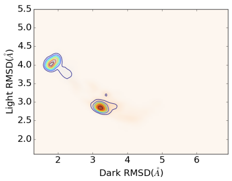

Table S2: Two dimensional (2D) RMSD contour plots of rigid residue scan (RRS) VVD Dark and Light MD simulations using RMSD values with references to both optimized Dark and Light states. Each residue number represents a simulation in which that particular residue is subjected to rigid body constraints. In each plot, Dark state attraction basin is illustrated mainly in green, and Light state attraction basin is illustrated mainly in brown.

Residue	Total Density	Residue	Total Density
13	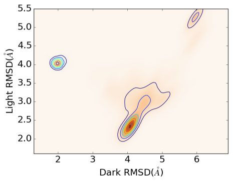	14	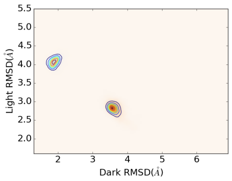
15	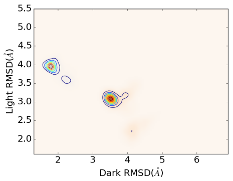	16	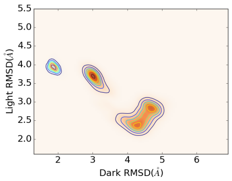
17	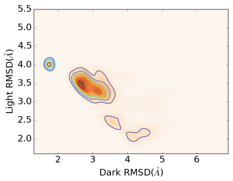	18	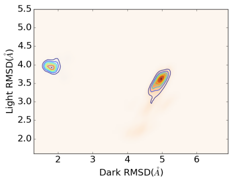
19	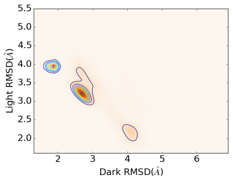	20	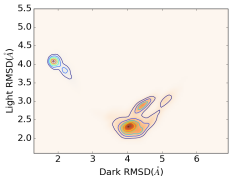
21	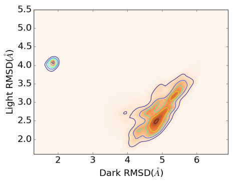	22	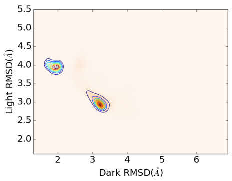
23	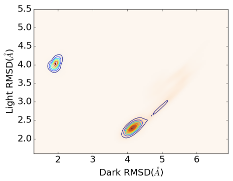	24	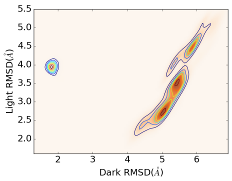
25	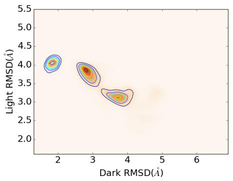	26	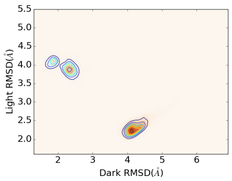

Table S2: Two dimensional (2D) RMSD contour plots of rigid residue scan (RRS) VVD Dark and Light MD simulations using RMSD values with references to both optimized Dark and Light states. Each residue number represents a simulation in which that particular residue is subjected to rigid body constraints. In each plot, Dark state attraction basin is illustrated mainly in green, and Light state attraction basin is illustrated mainly in brown.

Residue	Total Density	Residue	Total Density
27	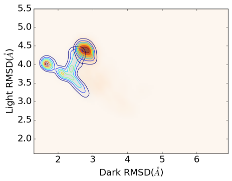	28	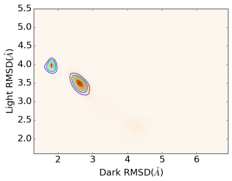
29	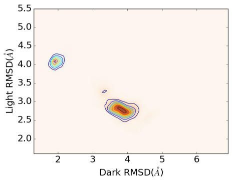	30	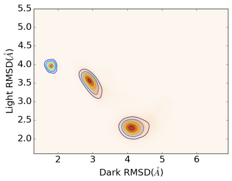
31	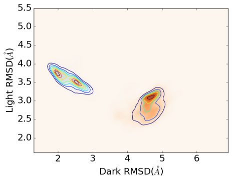	32	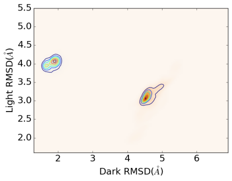
33	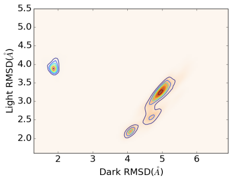	34	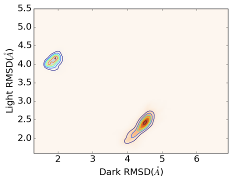
35	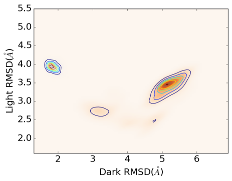	36	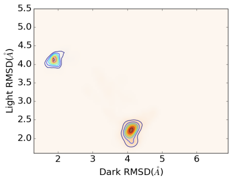
37	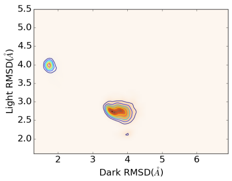	38	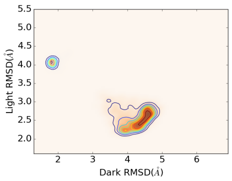
39	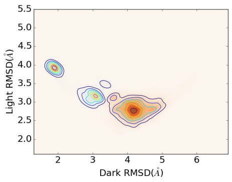	40	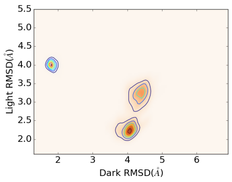

Table S2: Two dimensional (2D) RMSD contour plots of rigid residue scan (RRS) VVD Dark and Light MD simulations using RMSD values with references to both optimized Dark and Light states. Each residue number represents a simulation in which that particular residue is subjected to rigid body constraints. In each plot, Dark state attraction basin is illustrated mainly in green, and Light state attraction basin is illustrated mainly in brown.

Residue	Total Density	Residue	Total Density
41	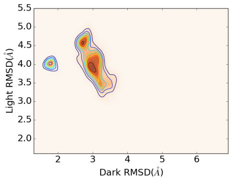	42	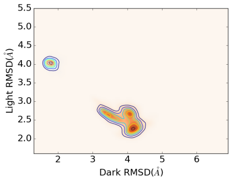
43	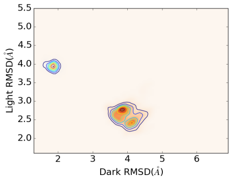	44	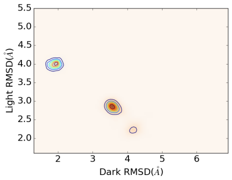
45	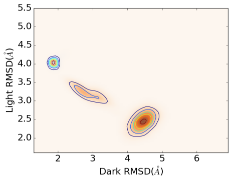	46	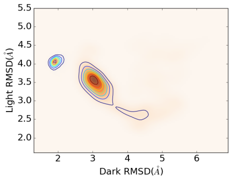
47	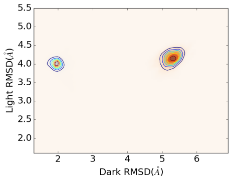	48	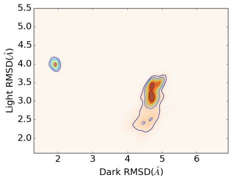
49	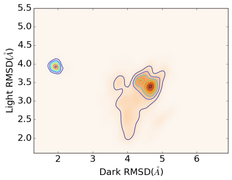	50	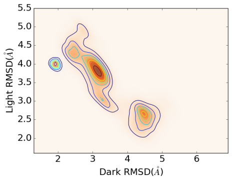
51	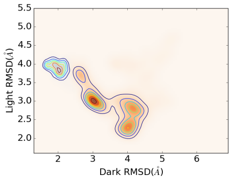	52	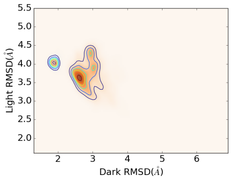
53	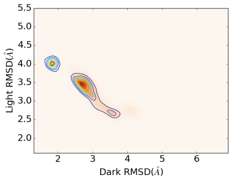	54	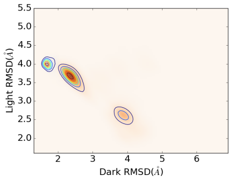

Table S2: Two dimensional (2D) RMSD contour plots of rigid residue scan (RRS) VVD Dark and Light MD simulations using RMSD values with references to both optimized Dark and Light states. Each residue number represents a simulation in which that particular residue is subjected to rigid body constraints. In each plot, Dark state attraction basin is illustrated mainly in green, and Light state attraction basin is illustrated mainly in brown.

Residue	Total Density	Residue	Total Density
55	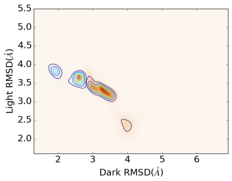	56	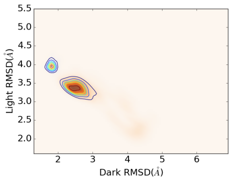
57	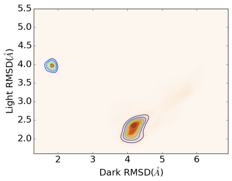	58	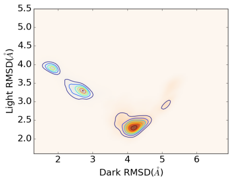
59	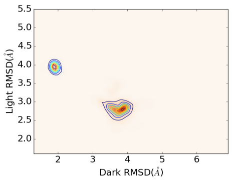	60	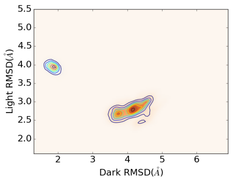
61	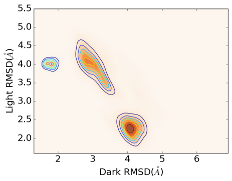	62	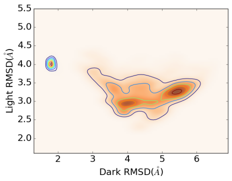
63	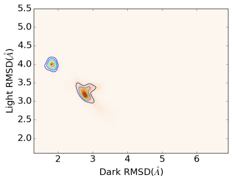	64	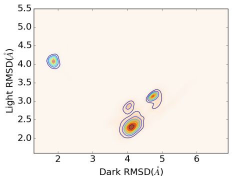
65	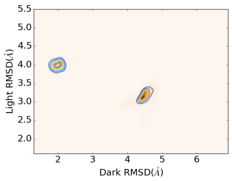	66	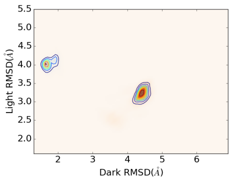
67	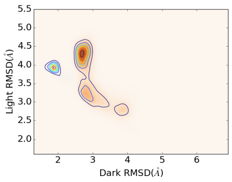	68	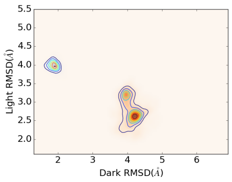

Table S2: Two dimensional (2D) RMSD contour plots of rigid residue scan (RRS) VVD Dark and Light MD simulations using RMSD values with references to both optimized Dark and Light states. Each residue number represents a simulation in which that particular residue is subjected to rigid body constraints. In each plot, Dark state attraction basin is illustrated mainly in green, and Light state attraction basin is illustrated mainly in brown.

Residue	Total Density	Residue	Total Density
69	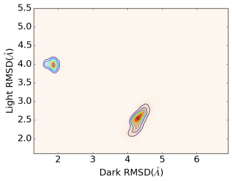	70	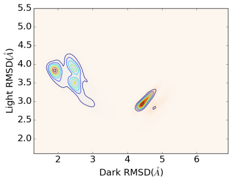
71	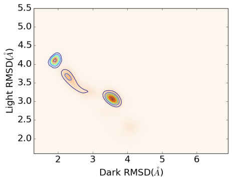	72	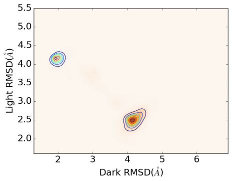
73	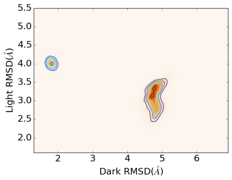	74	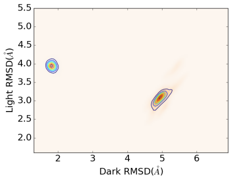
75	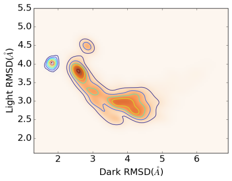	76	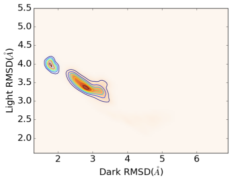
77	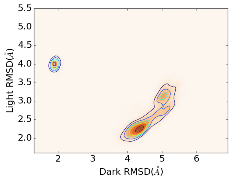	78	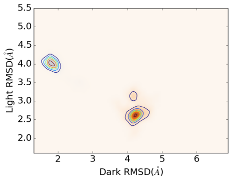
79	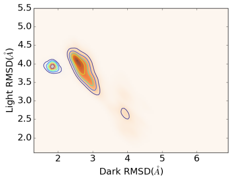	80	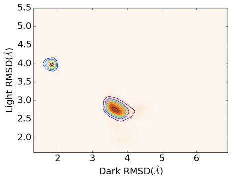
81	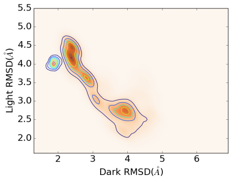	82	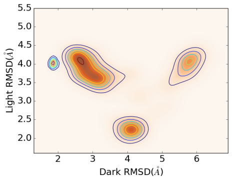

Table S2: Two dimensional (2D) RMSD contour plots of rigid residue scan (RRS) VVD Dark and Light MD simulations using RMSD values with references to both optimized Dark and Light states. Each residue number represents a simulation in which that particular residue is subjected to rigid body constraints. In each plot, Dark state attraction basin is illustrated mainly in green, and Light state attraction basin is illustrated mainly in brown.

Residue	Total Density	Residue	Total Density
83	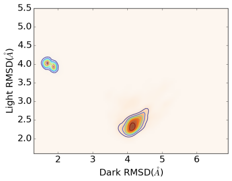	84	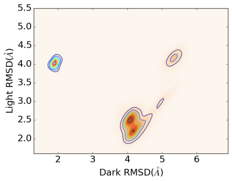
85	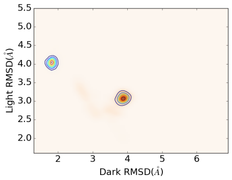	86	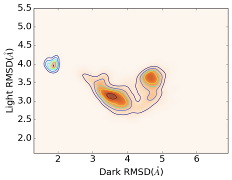
87	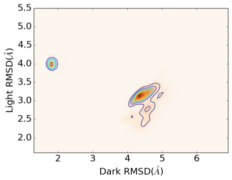	88	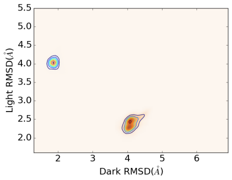
89	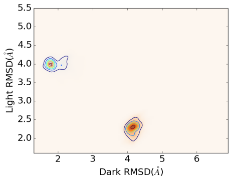	90	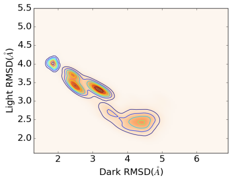
91	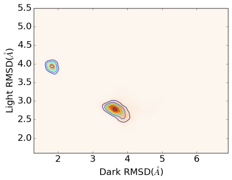	92	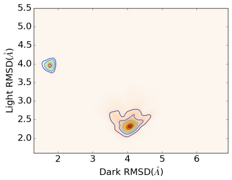
93	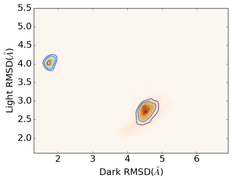	94	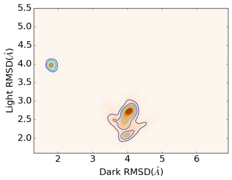
95	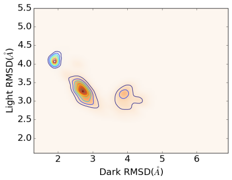	96	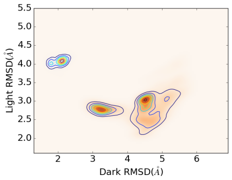

Table S2: Two dimensional (2D) RMSD contour plots of rigid residue scan (RRS) VVD Dark and Light MD simulations using RMSD values with references to both optimized Dark and Light states. Each residue number represents a simulation in which that particular residue is subjected to rigid body constraints. In each plot, Dark state attraction basin is illustrated mainly in green, and Light state attraction basin is illustrated mainly in brown.

Residue	Total Density	Residue	Total Density
97	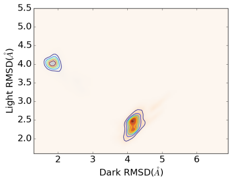	98	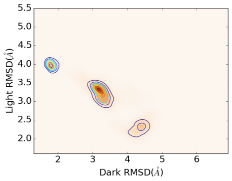
99	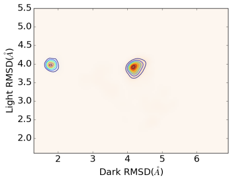	100	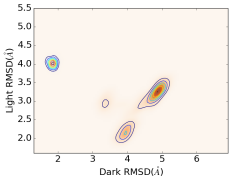
101	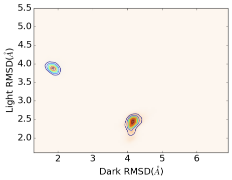	102	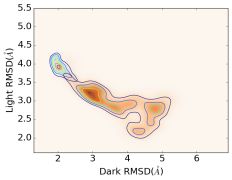
103	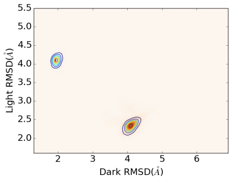	104	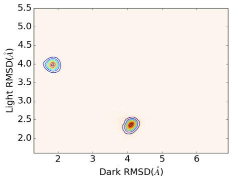
105	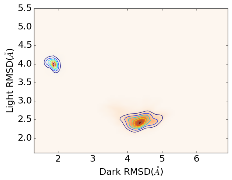	106	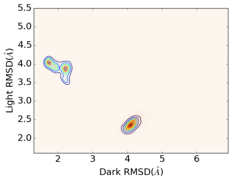
107	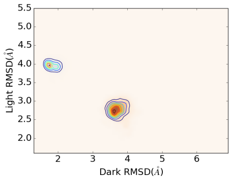	108	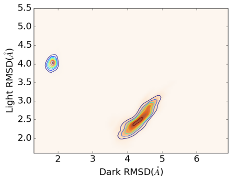
109	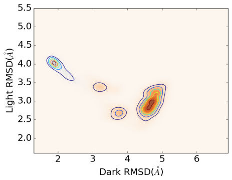	110	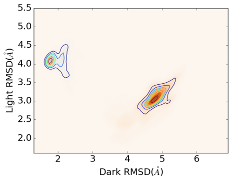

Table S2: Two dimensional (2D) RMSD contour plots of rigid residue scan (RRS) VVD Dark and Light MD simulations using RMSD values with references to both optimized Dark and Light states. Each residue number represents a simulation in which that particular residue is subjected to rigid body constraints. In each plot, Dark state attraction basin is illustrated mainly in green, and Light state attraction basin is illustrated mainly in brown.

<table>
<tr><th>Residue</th><th>Total Density</th><th>Residue</th><th>Total Density</th></tr>
<tr><td>111</td><td>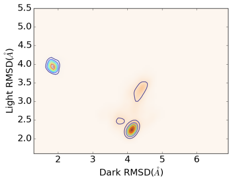
</td><td>112</td><td>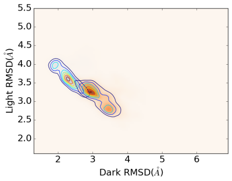
</td></tr>
<tr><td>113</td><td>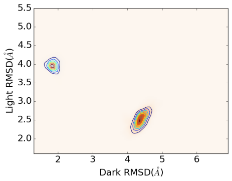
</td><td>114</td><td>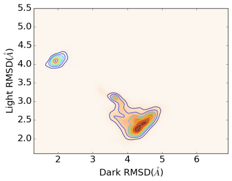
</td></tr>
<tr><td>115</td><td>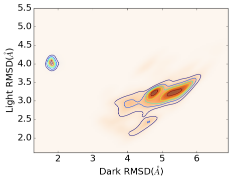
</td><td>116</td><td>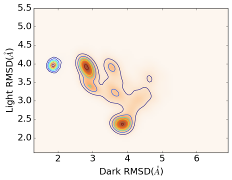
</td></tr>
<tr><td>117</td><td>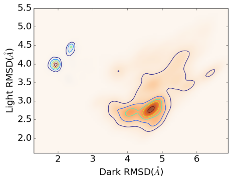
</td><td>118</td><td>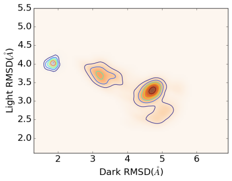
</td></tr>
<tr><td>119</td><td>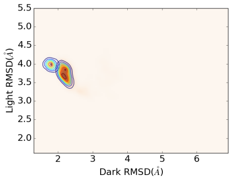
</td><td>120</td><td>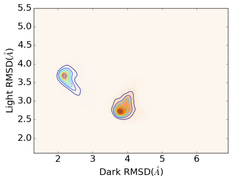
</td></tr>
<tr><td>121</td><td>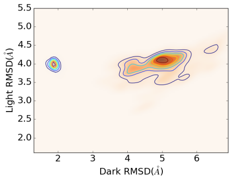
</td><td>122</td><td>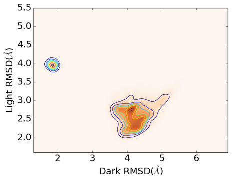
</td></tr>
<tr><td>123</td><td>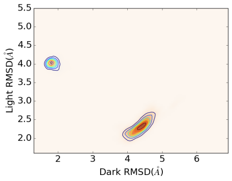
</td><td>124</td><td>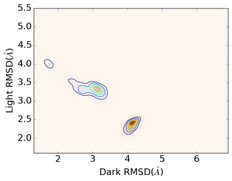
</td></tr>
</table>

Table S2: Two dimensional (2D) RMSD contour plots of rigid residue scan (RRS) VVD Dark and Light MD simulations using RMSD values with references to both optimized Dark and Light states. Each residue number represents a simulation in which that particular residue is subjected to rigid body constraints. In each plot, Dark state attraction basin is illustrated mainly in green, and Light state attraction basin is illustrated mainly in brown.

Residue	Total Density	Residue	Total Density
125	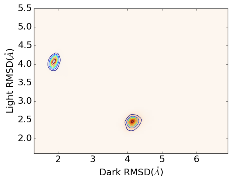	126	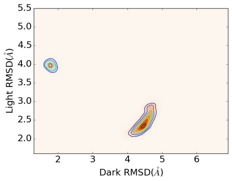
127	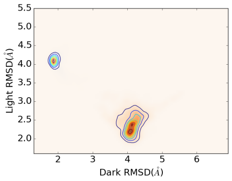	128	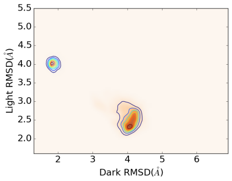
129	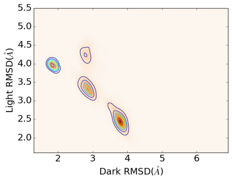	130	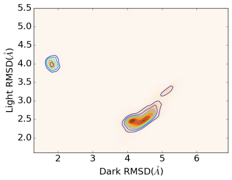
131	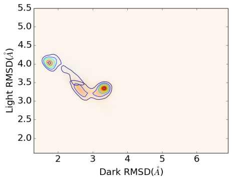	132	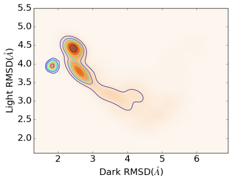
133	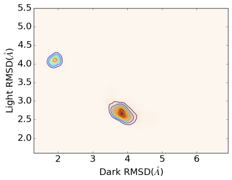	134	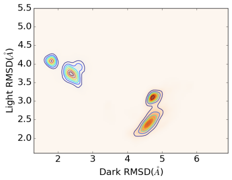
135	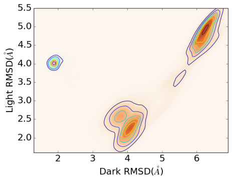	136	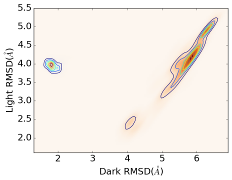
137	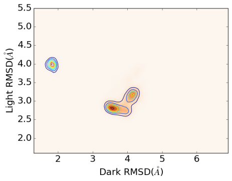	138	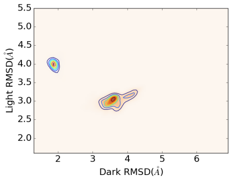

Table S2: Two dimensional (2D) RMSD contour plots of rigid residue scan (RRS) VVD Dark and Light MD simulations using RMSD values with references to both optimized Dark and Light states. Each residue number represents a simulation in which that particular residue is subjected to rigid body constraints. In each plot, Dark state attraction basin is illustrated mainly in green, and Light state attraction basin is illustrated mainly in brown.

Residue	Total Density	Residue	Total Density
139	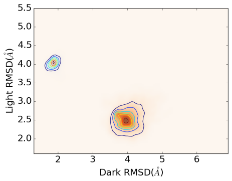	140	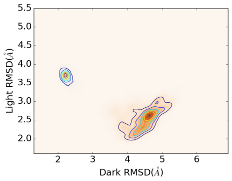
141	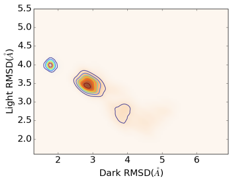	142	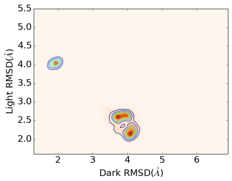
143	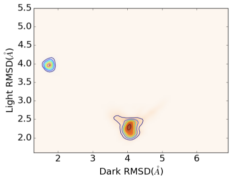	144	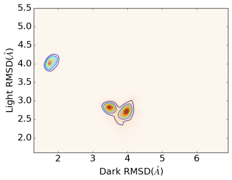
145	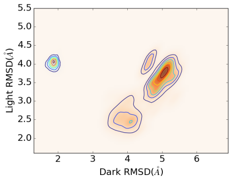	146	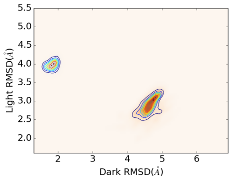
147	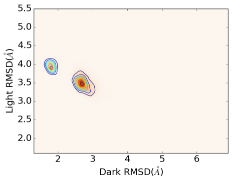	148	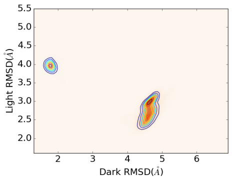

Table S3: Distributions (%) of the unperturbed and RRS simulations of VVD Dark and Light states among 15 clusters.

		Cluster														
Type	Res	1	2	3	4	5	6	7	8	9	10	11	12	13	14	15
Dark	0	18.9	10.3	18.3	18.2	15.7	0.2	3.9	3.2	9.1	1.4	0.1	0.6	0.0	0.0	0.0
Dark	1	97.7	0.0	0.0	0.0	0.0	2.3	0.0	0.0	0.0	0.0	0.0	0.0	0.0	0.0	0.0
Dark	2	99.9	0.1	0.0	0.0	0.0	0.0	0.0	0.0	0.0	0.0	0.0	0.0	0.0	0.0	0.0
Dark	3	100.0	0.0	0.0	0.0	0.0	0.0	0.0	0.0	0.0	0.0	0.0	0.0	0.0	0.0	0.0
Dark	4	100.0	0.0	0.0	0.0	0.0	0.0	0.0	0.0	0.0	0.0	0.0	0.0	0.0	0.0	0.0
Dark	5	98.4	1.1	0.0	0.0	0.0	0.5	0.0	0.0	0.0	0.0	0.0	0.0	0.0	0.0	0.0
Dark	6	99.9	0.1	0.0	0.0	0.0	0.0	0.0	0.0	0.0	0.0	0.0	0.0	0.0	0.0	0.0
Dark	7	98.4	1.6	0.0	0.0	0.0	0.0	0.0	0.0	0.0	0.0	0.0	0.0	0.0	0.0	0.0
Dark	8	99.7	0.3	0.0	0.0	0.0	0.0	0.0	0.0	0.0	0.0	0.0	0.0	0.0	0.0	0.0
Dark	9	100.0	0.0	0.0	0.0	0.0	0.0	0.0	0.0	0.0	0.0	0.0	0.0	0.0	0.0	0.0
Dark	10	100.0	0.0	0.0	0.0	0.0	0.0	0.0	0.0	0.0	0.0	0.0	0.0	0.0	0.0	0.0
Dark	11	99.6	0.2	0.0	0.0	0.0	0.2	0.0	0.0	0.0	0.0	0.0	0.0	0.0	0.0	0.0
Dark	12	82.3	16.6	0.5	0.0	0.0	0.6	0.0	0.0	0.0	0.0	0.0	0.0	0.0	0.0	0.0
Dark	13	96.1	1.3	0.0	0.0	0.0	2.7	0.0	0.0	0.0	0.0	0.0	0.0	0.0	0.0	0.0
Dark	14	99.6	0.1	0.0	0.0	0.0	0.3	0.0	0.0	0.0	0.0	0.0	0.0	0.0	0.0	0.0
Dark	15	79.1	20.8	0.0	0.0	0.0	0.0	0.0	0.0	0.0	0.0	0.0	0.0	0.0	0.0	0.0
Dark	16	98.3	1.7	0.0	0.0	0.0	0.0	0.0	0.0	0.0	0.0	0.0	0.0	0.0	0.0	0.0
Dark	17	99.3	0.7	0.0	0.0	0.0	0.0	0.0	0.0	0.0	0.0	0.0	0.0	0.0	0.0	0.0
Dark	18	92.1	5.7	0.0	0.0	0.0	2.2	0.0	0.0	0.0	0.0	0.0	0.0	0.0	0.0	0.0
Dark	19	99.6	0.4	0.0	0.0	0.0	0.0	0.0	0.0	0.0	0.0	0.0	0.0	0.0	0.0	0.0
Dark	20	73.1	26.7	0.0	0.0	0.0	0.2	0.0	0.0	0.0	0.0	0.0	0.0	0.0	0.0	0.0
Dark	21	100.0	0.0	0.0	0.0	0.0	0.0	0.0	0.0	0.0	0.0	0.0	0.0	0.0	0.0	0.0
Dark	22	98.8	1.2	0.0	0.0	0.0	0.1	0.0	0.0	0.0	0.0	0.0	0.0	0.0	0.0	0.0
Dark	23	99.7	0.3	0.0	0.0	0.0	0.1	0.0	0.0	0.0	0.0	0.0	0.0	0.0	0.0	0.0
Dark	24	99.8	0.2	0.0	0.0	0.0	0.0	0.0	0.0	0.0	0.0	0.0	0.0	0.0	0.0	0.0
Dark	25	100.0	0.0	0.0	0.0	0.0	0.0	0.0	0.0	0.0	0.0	0.0	0.0	0.0	0.0	0.0
Dark	26	37.5	50.7	0.0	0.0	0.0	11.8	0.0	0.0	0.0	0.0	0.0	0.0	0.0	0.0	0.0
Dark	27	30.5	34.8	8.2	0.0	0.0	26.5	0.0	0.0	0.0	0.0	0.0	0.0	0.0	0.0	0.0
Dark	28	100.0	0.0	0.0	0.0	0.0	0.0	0.0	0.0	0.0	0.0	0.0	0.0	0.0	0.0	0.0
Dark	29	98.1	0.1	0.0	0.0	0.0	1.8	0.0	0.0	0.0	0.0	0.0	0.0	0.0	0.0	0.0
Dark	30	99.7	0.3	0.0	0.0	0.0	0.0	0.0	0.0	0.0	0.0	0.0	0.0	0.0	0.0	0.0
Dark	31	25.4	58.9	14.5	0.0	0.0	1.2	0.0	0.0	0.0	0.0	0.0	0.0	0.0	0.0	0.0
Dark	32	99.8	0.1	0.0	0.0	0.0	0.0	0.0	0.0	0.0	0.0	0.0	0.0	0.0	0.0	0.0
Dark	33	99.7	0.3	0.0	0.0	0.0	0.0	0.0	0.0	0.0	0.0	0.0	0.0	0.0	0.0	0.0
Dark	34	99.8	0.2	0.0	0.0	0.0	0.1	0.0	0.0	0.0	0.0	0.0	0.0	0.0	0.0	0.0
Dark	35	98.8	1.2	0.0	0.0	0.0	0.0	0.0	0.0	0.0	0.0	0.0	0.0	0.0	0.0	0.0
Dark	36	93.9	0.2	0.0	0.0	0.0	5.9	0.0	0.0	0.0	0.0	0.0	0.0	0.0	0.0	0.0
Dark	37	99.3	0.7	0.0	0.0	0.0	0.0	0.0	0.0	0.0	0.0	0.0	0.0	0.0	0.0	0.0
Dark	38	98.3	0.1	0.0	0.0	0.0	1.6	0.0	0.0	0.0	0.0	0.0	0.0	0.0	0.0	0.0
Dark	39	35.4	5.9	57.6	1.0	0.0	0.0	0.0	0.0	0.0	0.0	0.0	0.0	0.0	0.0	0.0
Dark	40	99.9	0.1	0.0	0.0	0.0	0.0	0.0	0.0	0.0	0.0	0.0	0.0	0.0	0.0	0.0
Dark	41	99.8	0.2	0.0	0.0	0.0	0.0	0.0	0.0	0.0	0.0	0.0	0.0	0.0	0.0	0.0
Dark	42	99.7	0.3	0.0	0.0	0.0	0.0	0.0	0.0	0.0	0.0	0.0	0.0	0.0	0.0	0.0
Dark	43	99.5	0.5	0.0	0.0	0.0	0.0	0.0	0.0	0.0	0.0	0.0	0.0	0.0	0.0	0.0
Dark	44	99.6	0.4	0.0	0.0	0.0	0.0	0.0	0.0	0.0	0.0	0.0	0.0	0.0	0.0	0.0
Dark	45	99.4	0.0	0.0	0.0	0.0	0.6	0.0	0.0	0.0	0.0	0.0	0.0	0.0	0.0	0.0
Dark	46	98.3	0.8	0.0	0.0	0.0	0.9	0.0	0.0	0.0	0.0	0.0	0.0	0.0	0.0	0.0
Dark	47	94.4	4.7	0.0	0.0	0.0	0.9	0.0	0.0	0.0	0.0	0.0	0.0	0.0	0.0	0.0

Table S3: Distributions (%) of the unperturbed and RRS simulations of VVD Dark and Light states among 15 clusters.

		Cluster														
Type	Res	1	2	3	4	5	6	7	8	9	10	11	12	13	14	15
Dark	48	99.8	0.2	0.0	0.0	0.0	0.0	0.0	0.0	0.0	0.0	0.0	0.0	0.0	0.0	0.0
Dark	49	93.5	6.4	0.0	0.0	0.0	0.1	0.0	0.0	0.0	0.0	0.0	0.0	0.0	0.0	0.0
Dark	50	98.7	1.3	0.0	0.0	0.0	0.0	0.0	0.0	0.0	0.0	0.0	0.0	0.0	0.0	0.0
Dark	51	78.1	20.9	0.3	0.0	0.0	0.8	0.0	0.0	0.0	0.0	0.0	0.0	0.0	0.0	0.0
Dark	52	99.8	0.1	0.0	0.0	0.0	0.0	0.0	0.0	0.0	0.0	0.0	0.0	0.0	0.0	0.0
Dark	53	99.9	0.1	0.0	0.0	0.0	0.0	0.0	0.0	0.0	0.0	0.0	0.0	0.0	0.0	0.0
Dark	54	99.1	0.9	0.0	0.0	0.0	0.0	0.0	0.0	0.0	0.0	0.0	0.0	0.0	0.0	0.0
Dark	55	27.8	65.0	6.6	0.0	0.0	0.6	0.0	0.0	0.0	0.0	0.0	0.0	0.0	0.0	0.0
Dark	56	99.9	0.1	0.0	0.0	0.0	0.0	0.0	0.0	0.0	0.0	0.0	0.0	0.0	0.0	0.0
Dark	57	100.0	0.0	0.0	0.0	0.0	0.0	0.0	0.0	0.0	0.0	0.0	0.0	0.0	0.0	0.0
Dark	58	28.5	33.8	37.2	0.5	0.0	0.0	0.0	0.0	0.0	0.0	0.0	0.0	0.0	0.0	0.0
Dark	59	98.8	1.2	0.0	0.0	0.0	0.0	0.0	0.0	0.0	0.0	0.0	0.0	0.0	0.0	0.0
Dark	60	98.9	1.1	0.0	0.0	0.0	0.0	0.0	0.0	0.0	0.0	0.0	0.0	0.0	0.0	0.0
Dark	61	100.0	0.0	0.0	0.0	0.0	0.0	0.0	0.0	0.0	0.0	0.0	0.0	0.0	0.0	0.0
Dark	62	99.9	0.1	0.0	0.0	0.0	0.0	0.0	0.0	0.0	0.0	0.0	0.0	0.0	0.0	0.0
Dark	63	100.0	0.0	0.0	0.0	0.0	0.0	0.0	0.0	0.0	0.0	0.0	0.0	0.0	0.0	0.0
Dark	64	98.0	1.2	0.0	0.0	0.0	0.9	0.0	0.0	0.0	0.0	0.0	0.0	0.0	0.0	0.0
Dark	65	98.1	1.8	0.0	0.0	0.0	0.1	0.0	0.0	0.0	0.0	0.0	0.0	0.0	0.0	0.0
Dark	66	99.9	0.1	0.0	0.0	0.0	0.0	0.0	0.0	0.0	0.0	0.0	0.0	0.0	0.0	0.0
Dark	67	85.1	14.9	0.0	0.0	0.0	0.0	0.0	0.0	0.0	0.0	0.0	0.0	0.0	0.0	0.0
Dark	68	99.1	0.9	0.0	0.0	0.0	0.0	0.0	0.0	0.0	0.0	0.0	0.0	0.0	0.0	0.0
Dark	69	99.9	0.1	0.0	0.0	0.0	0.0	0.0	0.0	0.0	0.0	0.0	0.0	0.0	0.0	0.0
Dark	70	29.6	42.6	14.9	2.1	0.0	10.8	0.0	0.0	0.0	0.0	0.0	0.0	0.0	0.0	0.0
Dark	71	99.9	0.0	0.0	0.0	0.0	0.1	0.0	0.0	0.0	0.0	0.0	0.0	0.0	0.0	0.0
Dark	72	94.1	0.8	0.0	0.0	0.0	5.1	0.0	0.0	0.0	0.0	0.0	0.0	0.0	0.0	0.0
Dark	73	99.9	0.1	0.0	0.0	0.0	0.0	0.0	0.0	0.0	0.0	0.0	0.0	0.0	0.0	0.0
Dark	74	100.0	0.0	0.0	0.0	0.0	0.0	0.0	0.0	0.0	0.0	0.0	0.0	0.0	0.0	0.0
Dark	75	100.0	0.0	0.0	0.0	0.0	0.0	0.0	0.0	0.0	0.0	0.0	0.0	0.0	0.0	0.0
Dark	76	99.7	0.3	0.0	0.0	0.0	0.0	0.0	0.0	0.0	0.0	0.0	0.0	0.0	0.0	0.0
Dark	77	99.9	0.1	0.0	0.0	0.0	0.0	0.0	0.0	0.0	0.0	0.0	0.0	0.0	0.0	0.0
Dark	78	92.2	6.7	1.1	0.0	0.0	0.0	0.0	0.0	0.0	0.0	0.0	0.0	0.0	0.0	0.0
Dark	79	98.0	2.0	0.0	0.0	0.0	0.0	0.0	0.0	0.0	0.0	0.0	0.0	0.0	0.0	0.0
Dark	80	99.8	0.2	0.0	0.0	0.0	0.0	0.0	0.0	0.0	0.0	0.0	0.0	0.0	0.0	0.0
Dark	81	99.5	0.4	0.0	0.0	0.0	0.0	0.0	0.0	0.0	0.0	0.0	0.0	0.0	0.0	0.0
Dark	82	100.0	0.0	0.0	0.0	0.0	0.0	0.0	0.0	0.0	0.0	0.0	0.0	0.0	0.0	0.0
Dark	83	100.0	0.0	0.0	0.0	0.0	0.0	0.0	0.0	0.0	0.0	0.0	0.0	0.0	0.0	0.0
Dark	84	99.9	0.1	0.0	0.0	0.0	0.0	0.0	0.0	0.0	0.0	0.0	0.0	0.0	0.0	0.0
Dark	85	99.9	0.1	0.0	0.0	0.0	0.0	0.0	0.0	0.0	0.0	0.0	0.0	0.0	0.0	0.0
Dark	86	100.0	0.0	0.0	0.0	0.0	0.0	0.0	0.0	0.0	0.0	0.0	0.0	0.0	0.0	0.0
Dark	87	99.8	0.1	0.0	0.0	0.0	0.2	0.0	0.0	0.0	0.0	0.0	0.0	0.0	0.0	0.0
Dark	88	99.7	0.3	0.0	0.0	0.0	0.0	0.0	0.0	0.0	0.0	0.0	0.0	0.0	0.0	0.0
Dark	89	85.5	8.5	0.0	0.0	0.0	6.0	0.0	0.0	0.0	0.0	0.0	0.0	0.0	0.0	0.0
Dark	90	99.8	0.2	0.0	0.0	0.0	0.0	0.0	0.0	0.0	0.0	0.0	0.0	0.0	0.0	0.0
Dark	91	99.4	0.6	0.0	0.0	0.0	0.0	0.0	0.0	0.0	0.0	0.0	0.0	0.0	0.0	0.0
Dark	92	100.0	0.0	0.0	0.0	0.0	0.0	0.0	0.0	0.0	0.0	0.0	0.0	0.0	0.0	0.0
Dark	93	99.9	0.1	0.0	0.0	0.0	0.0	0.0	0.0	0.0	0.0	0.0	0.0	0.0	0.0	0.0
Dark	94	100.0	0.0	0.0	0.0	0.0	0.0	0.0	0.0	0.0	0.0	0.0	0.0	0.0	0.0	0.0
Dark	95	99.4	0.0	0.0	0.0	0.0	0.5	0.0	0.0	0.0	0.0	0.0	0.0	0.0	0.0	0.0

Table S3: Distributions (%) of the unperturbed and RRS simulations of VVD Dark and Light states among 15 clusters.

		Cluster														
Type	Res	1	2	3	4	5	6	7	8	9	10	11	12	13	14	15
Dark	96	91.2	1.4	0.0	0.0	0.0	7.4	0.0	0.0	0.0	0.0	0.0	0.0	0.0	0.0	0.0
Dark	97	86.1	11.4	2.4	0.0	0.0	0.0	0.0	0.0	0.0	0.0	0.0	0.0	0.0	0.0	0.0
Dark	98	99.9	0.1	0.0	0.0	0.0	0.0	0.0	0.0	0.0	0.0	0.0	0.0	0.0	0.0	0.0
Dark	99	100.0	0.0	0.0	0.0	0.0	0.0	0.0	0.0	0.0	0.0	0.0	0.0	0.0	0.0	0.0
Dark	100	100.0	0.0	0.0	0.0	0.0	0.0	0.0	0.0	0.0	0.0	0.0	0.0	0.0	0.0	0.0
Dark	101	99.0	1.0	0.0	0.0	0.0	0.0	0.0	0.0	0.0	0.0	0.0	0.0	0.0	0.0	0.0
Dark	102	67.8	31.2	0.6	0.0	0.0	0.4	0.0	0.0	0.0	0.0	0.0	0.0	0.0	0.0	0.0
Dark	103	99.7	0.2	0.0	0.0	0.0	0.1	0.0	0.0	0.0	0.0	0.0	0.0	0.0	0.0	0.0
Dark	104	89.0	3.9	6.3	0.5	0.0	0.0	0.0	0.0	0.0	0.0	0.3	0.0	0.0	0.0	0.0
Dark	105	99.7	0.3	0.0	0.0	0.0	0.0	0.0	0.0	0.0	0.0	0.0	0.0	0.0	0.0	0.0
Dark	106	59.8	38.2	0.0	0.0	0.0	1.9	0.0	0.0	0.0	0.0	0.0	0.0	0.0	0.0	0.0
Dark	107	98.8	1.1	0.0	0.0	0.0	0.1	0.0	0.0	0.0	0.0	0.0	0.0	0.0	0.0	0.0
Dark	108	100.0	0.0	0.0	0.0	0.0	0.0	0.0	0.0	0.0	0.0	0.0	0.0	0.0	0.0	0.0
Dark	109	66.1	29.4	4.4	0.0	0.0	0.1	0.0	0.0	0.0	0.0	0.0	0.0	0.0	0.0	0.0
Dark	110	79.1	12.7	0.0	0.0	0.0	8.1	0.0	0.0	0.0	0.0	0.0	0.0	0.0	0.0	0.0
Dark	111	99.8	0.2	0.0	0.0	0.0	0.0	0.0	0.0	0.0	0.0	0.0	0.0	0.0	0.0	0.0
Dark	112	22.3	58.0	12.4	0.1	0.0	7.3	0.0	0.0	0.0	0.0	0.0	0.0	0.0	0.0	0.0
Dark	113	99.7	0.3	0.0	0.0	0.0	0.0	0.0	0.0	0.0	0.0	0.0	0.0	0.0	0.0	0.0
Dark	114	93.8	0.8	0.0	0.0	0.0	5.4	0.0	0.0	0.0	0.0	0.0	0.0	0.0	0.0	0.0
Dark	115	100.0	0.0	0.0	0.0	0.0	0.0	0.0	0.0	0.0	0.0	0.0	0.0	0.0	0.0	0.0
Dark	116	99.7	0.2	0.0	0.0	0.0	0.1	0.0	0.0	0.0	0.0	0.0	0.0	0.0	0.0	0.0
Dark	117	58.2	8.4	0.2	0.0	0.0	33.2	0.0	0.0	0.0	0.0	0.0	0.0	0.0	0.0	0.0
Dark	118	99.9	0.1	0.0	0.0	0.0	0.0	0.0	0.0	0.0	0.0	0.0	0.0	0.0	0.0	0.0
Dark	119	99.8	0.2	0.0	0.0	0.0	0.0	0.0	0.0	0.0	0.0	0.0	0.0	0.0	0.0	0.0
Dark	120	3.8	78.2	10.3	1.7	0.3	4.1	0.7	0.3	0.3	0.0	0.3	0.0	0.0	0.0	0.0
Dark	121	99.2	0.8	0.0	0.0	0.0	0.0	0.0	0.0	0.0	0.0	0.0	0.0	0.0	0.0	0.0
Dark	122	99.5	0.5	0.0	0.0	0.0	0.0	0.0	0.0	0.0	0.0	0.0	0.0	0.0	0.0	0.0
Dark	123	99.3	0.6	0.0	0.0	0.0	0.1	0.0	0.0	0.0	0.0	0.0	0.0	0.0	0.0	0.0
Dark	124	11.4	18.6	68.1	1.9	0.0	0.0	0.0	0.0	0.0	0.0	0.0	0.0	0.0	0.0	0.0
Dark	125	99.0	1.0	0.0	0.0	0.0	0.0	0.0	0.0	0.0	0.0	0.0	0.0	0.0	0.0	0.0
Dark	126	99.7	0.3	0.0	0.0	0.0	0.0	0.0	0.0	0.0	0.0	0.0	0.0	0.0	0.0	0.0
Dark	127	92.6	6.7	0.2	0.0	0.0	0.6	0.0	0.0	0.0	0.0	0.0	0.0	0.0	0.0	0.0
Dark	128	99.9	0.1	0.0	0.0	0.0	0.0	0.0	0.0	0.0	0.0	0.0	0.0	0.0	0.0	0.0
Dark	129	99.3	0.7	0.0	0.0	0.0	0.0	0.0	0.0	0.0	0.0	0.0	0.0	0.0	0.0	0.0
Dark	130	99.8	0.2	0.0	0.0	0.0	0.0	0.0	0.0	0.0	0.0	0.0	0.0	0.0	0.0	0.0
Dark	131	60.2	28.7	7.1	0.0	0.0	4.0	0.0	0.0	0.0	0.0	0.0	0.0	0.0	0.0	0.0
Dark	132	99.9	0.1	0.0	0.0	0.0	0.0	0.0	0.0	0.0	0.0	0.0	0.0	0.0	0.0	0.0
Dark	133	99.7	0.1	0.0	0.0	0.0	0.2	0.0	0.0	0.0	0.0	0.0	0.0	0.0	0.0	0.0
Dark	134	28.8	59.7	0.8	0.0	0.0	10.7	0.0	0.0	0.0	0.0	0.0	0.0	0.0	0.0	0.0
Dark	135	95.4	3.4	0.0	0.0	0.0	1.2	0.0	0.0	0.0	0.0	0.0	0.0	0.0	0.0	0.0
Dark	136	99.1	0.8	0.0	0.0	0.0	0.2	0.0	0.0	0.0	0.0	0.0	0.0	0.0	0.0	0.0
Dark	137	100.0	0.0	0.0	0.0	0.0	0.0	0.0	0.0	0.0	0.0	0.0	0.0	0.0	0.0	0.0
Dark	138	99.5	0.5	0.0	0.0	0.0	0.0	0.0	0.0	0.0	0.0	0.0	0.0	0.0	0.0	0.0
Dark	139	99.0	1.0	0.0	0.0	0.0	0.0	0.0	0.0	0.0	0.0	0.0	0.0	0.0	0.0	0.0
Dark	140	8.2	90.9	0.8	0.0	0.0	0.1	0.0	0.0	0.0	0.0	0.0	0.0	0.0	0.0	0.0
Dark	141	100.0	0.0	0.0	0.0	0.0	0.0	0.0	0.0	0.0	0.0	0.0	0.0	0.0	0.0	0.0
Dark	142	99.9	0.1	0.0	0.0	0.0	0.1	0.0	0.0	0.0	0.0	0.0	0.0	0.0	0.0	0.0
Dark	143	100.0	0.0	0.0	0.0	0.0	0.0	0.0	0.0	0.0	0.0	0.0	0.0	0.0	0.0	0.0

Table S3: Distributions (%) of the unperturbed and RRS simulations of VVD Dark and Light states among 15 clusters.

		Cluster														
Type	Res	1	2	3	4	5	6	7	8	9	10	11	12	13	14	15
Dark	144	100.0	0.0	0.0	0.0	0.0	0.0	0.0	0.0	0.0	0.0	0.0	0.0	0.0	0.0	0.0
Dark	145	100.0	0.0	0.0	0.0	0.0	0.0	0.0	0.0	0.0	0.0	0.0	0.0	0.0	0.0	0.0
Dark	146	100.0	0.0	0.0	0.0	0.0	0.0	0.0	0.0	0.0	0.0	0.0	0.0	0.0	0.0	0.0
Dark	147	99.8	0.2	0.0	0.0	0.0	0.0	0.0	0.0	0.0	0.0	0.0	0.0	0.0	0.0	0.0
Dark	148	99.2	0.8	0.0	0.0	0.0	0.0	0.0	0.0	0.0	0.0	0.0	0.0	0.0	0.0	0.0
Light	0	0.0	0.0	0.0	0.0	0.2	0.0	5.0	6.1	20.8	49.6	0.0	0.7	14.5	1.8	1.2
Light	1	0.0	0.0	0.0	0.0	1.2	0.0	45.2	52.3	1.1	0.3	0.0	0.0	0.0	0.0	0.0
Light	2	0.0	0.0	0.0	0.1	0.0	0.0	88.9	10.9	0.1	0.0	0.0	0.0	0.0	0.0	0.0
Light	3	0.0	0.0	0.0	0.1	0.0	0.0	95.9	4.0	0.1	0.0	0.0	0.0	0.0	0.0	0.0
Light	4	0.0	0.0	0.0	99.1	0.5	0.0	0.4	0.0	0.0	0.0	0.0	0.0	0.0	0.0	0.0
Light	5	0.0	0.0	0.0	0.0	0.2	0.0	0.0	2.7	19.4	44.9	5.1	23.9	3.3	0.4	0.0
Light	6	0.0	3.5	52.7	14.7	16.6	5.2	4.1	1.2	0.2	0.0	1.8	0.0	0.0	0.0	0.0
Light	7	0.0	5.6	79.8	10.9	0.0	2.5	0.0	0.0	0.0	0.0	1.2	0.0	0.0	0.0	0.0
Light	8	0.0	0.0	0.0	0.0	0.1	0.0	41.5	56.5	2.0	0.0	0.0	0.0	0.0	0.0	0.0
Light	9	0.0	0.0	0.0	0.1	0.0	0.0	90.3	4.2	4.7	0.7	0.0	0.0	0.0	0.0	0.0
Light	10	0.0	0.0	0.0	0.0	12.2	0.0	3.3	45.9	38.6	0.0	0.0	0.0	0.0	0.0	0.0
Light	11	0.0	7.3	55.8	0.0	0.0	35.0	0.0	0.0	0.0	0.0	1.9	0.0	0.0	0.0	0.0
Light	12	0.0	2.7	29.8	55.2	1.7	0.0	6.3	3.7	0.7	0.0	0.0	0.0	0.0	0.0	0.0
Light	13	0.0	0.0	2.9	6.2	17.3	0.1	29.9	4.0	14.0	4.0	5.7	1.6	1.1	3.8	9.5
Light	14	0.0	0.0	0.5	94.8	0.1	0.0	4.4	0.2	0.0	0.0	0.0	0.0	0.0	0.0	0.0
Light	15	0.0	0.0	4.4	65.7	9.1	0.0	15.6	3.4	0.0	0.0	1.8	0.0	0.0	0.0	0.0
Light	16	0.0	0.3	24.9	4.8	4.3	3.9	22.2	31.5	8.0	0.0	0.0	0.0	0.0	0.0	0.0
Light	17	0.0	19.1	46.1	11.9	2.2	2.1	9.4	5.1	1.6	0.3	2.2	0.0	0.0	0.0	0.0
Light	18	0.0	0.0	0.0	0.1	2.2	0.0	9.3	9.0	38.5	28.2	0.0	12.7	0.0	0.0	0.0
Light	19	0.0	18.3	45.2	6.8	2.2	5.3	19.6	0.5	0.1	0.0	1.7	0.3	0.0	0.0	0.0
Light	20	0.0	0.0	0.1	6.8	11.9	0.0	53.0	17.3	9.5	1.5	0.0	0.0	0.0	0.0	0.0
Light	21	0.0	0.0	0.0	3.8	3.4	0.0	14.0	36.9	21.9	18.0	0.0	0.0	2.0	0.0	0.0
Light	22	0.0	6.5	46.1	35.1	2.7	8.1	0.7	0.2	0.0	0.0	0.5	0.0	0.0	0.0	0.0
Light	23	0.0	0.0	0.0	0.0	0.2	0.0	59.3	9.7	12.8	12.5	0.0	0.3	4.9	0.3	0.0
Light	24	0.0	0.0	0.0	0.0	0.0	0.0	4.0	18.7	16.4	29.0	0.0	3.3	11.3	11.3	6.0
Light	25	0.0	6.1	23.9	26.3	11.0	22.1	1.4	3.3	5.2	0.0	0.7	0.0	0.0	0.0	0.0
Light	26	0.0	0.0	0.0	0.0	0.0	0.0	81.5	17.4	1.0	0.1	0.0	0.0	0.0	0.0	0.0
Light	27	0.0	3.6	15.4	4.8	1.6	72.7	0.1	0.0	0.1	0.0	1.6	0.0	0.0	0.0	0.0
Light	28	0.0	60.3	22.2	3.7	0.0	0.1	11.2	2.6	0.0	0.0	0.0	0.0	0.0	0.0	0.0
Light	29	0.0	0.0	6.0	67.3	12.2	0.0	11.2	3.3	0.0	0.0	0.0	0.0	0.0	0.0	0.0
Light	30	0.0	2.8	36.6	1.1	0.2	1.0	46.1	10.8	1.2	0.0	0.0	0.0	0.0	0.0	0.0
Light	31	0.0	0.0	0.0	4.0	18.4	0.0	6.4	33.9	36.4	0.7	0.1	0.0	0.0	0.0	0.0
Light	32	0.0	0.0	0.0	0.0	41.4	0.0	3.7	7.8	40.3	5.5	0.0	0.6	0.8	0.0	0.0
Light	33	0.0	0.0	0.0	0.0	1.9	0.0	15.1	21.9	40.0	18.8	0.0	0.0	2.2	0.0	0.0
Light	34	0.0	0.0	0.0	0.0	0.2	0.0	42.5	56.5	0.8	0.0	0.0	0.0	0.0	0.0	0.0
Light	35	0.0	0.0	4.7	11.8	0.6	0.0	4.8	7.3	22.3	43.9	0.0	0.0	4.6	0.0	0.0
Light	36	0.0	0.0	3.7	3.5	0.6	0.1	85.0	6.5	0.0	0.0	0.5	0.0	0.0	0.0	0.0
Light	37	0.0	0.0	0.0	74.3	8.7	0.0	15.9	1.0	0.0	0.0	0.0	0.0	0.0	0.0	0.0
Light	38	0.0	0.0	0.8	15.1	5.8	0.0	36.4	40.0	1.9	0.0	0.0	0.0	0.0	0.0	0.0
Light	39	0.0	0.0	0.0	24.8	32.5	0.0	13.2	18.9	9.1	1.2	0.0	0.0	0.2	0.0	0.0
Light	40	0.0	0.0	0.0	2.8	36.4	0.0	45.3	3.4	5.8	0.0	6.1	0.1	0.0	0.0	0.0
Light	41	0.0	0.4	30.2	1.2	0.0	65.9	0.0	0.0	0.0	0.0	2.4	0.0	0.0	0.0	0.0
Light	42	0.0	0.0	0.7	37.1	5.5	0.0	51.9	4.9	0.0	0.0	0.0	0.0	0.0	0.0	0.0

Table S3: Distributions (%) of the unperturbed and RRS simulations of VVD Dark and Light states among 15 clusters.

		Cluster														
Type	Res	1	2	3	4	5	6	7	8	9	10	11	12	13	14	15
Light	43	0.0	0.0	0.0	46.1	9.1	0.0	34.5	10.0	0.3	0.0	0.0	0.0	0.0	0.0	0.0
Light	44	0.0	0.0	0.2	77.7	0.1	0.0	20.9	1.2	0.0	0.0	0.0	0.0	0.0	0.0	0.0
Light	45	0.0	6.8	19.1	2.8	0.2	0.0	26.3	44.2	0.7	0.0	0.0	0.0	0.0	0.0	0.0
Light	46	0.0	1.9	55.1	7.4	1.7	7.6	3.7	8.0	5.5	1.8	1.5	4.0	1.4	0.4	0.0
Light	47	0.0	0.0	0.0	0.0	0.0	0.0	0.0	0.0	0.1	0.9	0.0	72.3	26.4	0.3	0.0
Light	48	0.0	0.0	0.0	0.2	9.7	0.0	8.6	23.0	53.1	5.0	0.0	0.4	0.0	0.0	0.0
Light	49	0.0	0.0	1.6	10.1	18.1	0.0	12.5	6.7	23.8	2.0	20.7	4.6	0.0	0.0	0.0
Light	50	0.3	1.1	27.1	6.3	2.5	35.2	6.8	18.5	1.9	0.0	0.3	0.0	0.0	0.0	0.0
Light	51	0.0	6.7	24.8	14.8	13.9	1.2	23.0	5.3	2.7	0.1	5.0	2.6	0.0	0.1	0.0
Light	52	0.0	31.4	33.9	2.9	0.0	30.6	0.0	0.0	0.0	0.0	1.2	0.0	0.0	0.0	0.0
Light	53	0.0	26.6	49.6	19.9	2.2	0.2	1.3	0.3	0.0	0.0	0.0	0.0	0.0	0.0	0.0
Light	54	0.7	49.4	7.8	17.4	1.6	0.4	16.8	1.2	0.0	0.0	3.8	0.8	0.0	0.0	0.0
Light	55	0.0	0.3	58.7	20.7	0.2	1.4	17.9	0.7	0.0	0.0	0.0	0.0	0.0	0.0	0.0
Light	56	0.0	52.9	25.3	5.9	1.4	0.1	11.6	2.7	0.0	0.0	0.1	0.0	0.0	0.0	0.0
Light	57	0.0	0.0	0.0	2.6	1.4	0.0	67.0	14.3	5.1	8.3	0.0	0.0	1.3	0.0	0.0
Light	58	0.0	0.0	0.0	10.8	1.9	0.0	58.6	15.3	7.6	5.8	0.0	0.0	0.0	0.0	0.0
Light	59	0.0	0.0	3.4	80.4	9.4	0.0	5.0	0.3	0.2	0.0	1.4	0.0	0.0	0.0	0.0
Light	60	0.0	0.0	0.0	33.8	40.5	0.0	9.0	12.0	4.6	0.0	0.0	0.0	0.0	0.0	0.0
Light	61	0.0	0.9	14.3	3.0	2.1	33.2	39.0	4.2	0.2	0.0	3.1	0.0	0.0	0.0	0.0
Light	62	0.0	0.1	7.5	15.0	16.9	3.1	1.0	5.8	16.5	20.7	9.0	1.2	3.1	0.1	0.0
Light	63	0.0	12.5	79.7	7.7	0.0	0.0	0.1	0.0	0.0	0.0	0.0	0.0	0.0	0.0	0.0
Light	64	0.0	0.0	0.0	6.3	7.8	0.0	54.0	9.2	22.0	0.8	0.0	0.0	0.1	0.0	0.0
Light	65	0.0	0.0	0.0	2.0	72.7	0.0	0.0	2.4	22.7	0.1	0.0	0.1	0.0	0.0	0.0
Light	66	0.0	0.0	0.0	9.8	75.4	0.0	2.2	0.6	8.9	0.0	3.2	0.0	0.0	0.0	0.0
Light	67	0.0	3.2	28.2	15.8	1.7	50.6	0.5	0.0	0.0	0.0	0.0	0.0	0.0	0.0	0.0
Light	68	0.0	0.0	0.0	14.8	29.8	0.0	35.0	17.4	0.7	0.0	2.4	0.0	0.0	0.0	0.0
Light	69	0.0	0.0	0.0	0.0	5.7	0.0	47.4	45.8	1.2	0.0	0.0	0.0	0.0	0.0	0.0
Light	70	0.0	0.0	0.0	0.0	55.6	0.0	0.5	12.5	30.4	1.0	0.0	0.0	0.0	0.0	0.0
Light	71	0.7	26.9	13.3	48.0	0.1	0.1	10.9	0.1	0.0	0.0	0.0	0.0	0.0	0.0	0.0
Light	72	0.0	0.7	5.4	3.5	2.0	1.3	64.5	21.9	0.7	0.0	0.0	0.0	0.0	0.0	0.0
Light	73	0.0	0.0	0.0	0.0	6.4	0.0	0.0	20.6	67.3	5.6	0.0	0.1	0.0	0.0	0.0
Light	74	0.0	0.0	0.0	0.0	0.4	0.0	0.0	9.9	73.3	12.6	0.0	1.0	2.9	0.0	0.0
Light	75	0.0	13.2	16.6	22.4	18.2	11.2	3.3	8.4	4.3	1.2	0.7	0.0	0.6	0.0	0.0
Light	76	0.0	31.9	55.6	5.1	0.0	0.1	2.8	4.4	0.2	0.0	0.0	0.0	0.0	0.0	0.0
Light	77	0.0	0.0	0.0	0.1	0.3	0.0	40.4	25.1	27.1	7.0	0.0	0.0	0.0	0.0	0.0
Light	78	0.0	0.0	0.0	6.7	28.0	0.0	34.1	29.8	1.2	0.0	0.2	0.0	0.0	0.0	0.0
Light	79	0.0	13.8	22.5	8.3	2.3	43.2	8.6	0.7	0.0	0.0	0.7	0.0	0.0	0.0	0.0
Light	80	0.0	0.0	0.3	79.7	4.5	0.0	12.2	3.2	0.1	0.0	0.0	0.0	0.0	0.0	0.0
Light	81	0.8	8.6	16.6	21.2	6.8	27.6	11.6	5.4	0.6	0.0	0.8	0.0	0.0	0.0	0.0
Light	82	0.0	6.3	19.3	0.8	1.9	26.7	18.4	1.5	1.7	4.0	3.9	0.0	13.8	1.8	0.0
Light	83	0.0	0.0	0.1	6.1	5.4	0.0	63.3	21.0	3.4	0.7	0.1	0.0	0.0	0.0	0.0
Light	84	0.0	0.0	0.0	3.3	2.2	0.0	61.8	11.8	4.9	1.9	0.0	9.2	4.9	0.0	0.0
Light	85	0.0	3.6	11.5	64.3	19.3	0.0	1.2	0.0	0.0	0.0	0.1	0.0	0.0	0.0	0.0
Light	86	0.0	1.2	18.7	30.4	11.8	0.3	0.8	2.1	16.2	1.7	10.2	6.6	0.0	0.0	0.0
Light	87	0.0	0.0	0.0	0.4	43.6	0.0	8.9	19.2	26.0	1.4	0.0	0.5	0.0	0.0	0.0
Light	88	0.0	0.0	0.0	0.1	0.2	0.0	88.7	11.1	0.0	0.0	0.0	0.0	0.0	0.0	0.0
Light	89	0.0	0.0	0.0	0.0	0.0	0.0	93.9	6.1	0.0	0.0	0.0	0.0	0.0	0.0	0.0
Light	90	0.0	24.3	27.7	10.3	1.6	0.0	17.7	17.5	1.0	0.0	0.0	0.0	0.0	0.0	0.0

Table S3: Distributions (%) of the unperturbed and RRS simulations of VVD Dark and Light states among 15 clusters.

		Cluster														
Type	Res	1	2	3	4	5	6	7	8	9	10	11	12	13	14	15
Light	91	0.0	0.0	0.4	81.3	4.9	0.0	10.8	2.0	0.4	0.0	0.1	0.0	0.0	0.0	0.0
Light	92	0.0	0.0	0.1	19.0	8.0	0.0	59.6	12.8	0.4	0.0	0.1	0.0	0.0	0.0	0.0
Light	93	0.0	0.0	0.0	0.1	13.9	0.0	12.0	61.7	12.2	0.1	0.0	0.0	0.0	0.0	0.0
Light	94	0.0	0.0	0.0	27.9	18.0	0.0	47.4	6.2	0.4	0.0	0.0	0.0	0.0	0.0	0.0
Light	95	0.0	21.5	41.3	16.1	15.2	1.7	0.9	0.2	0.2	0.0	3.0	0.0	0.0	0.0	0.0
Light	96	0.0	0.0	6.6	19.5	15.8	0.0	6.1	26.5	16.2	7.6	0.1	0.8	0.9	0.0	0.0
Light	97	0.0	0.0	0.0	0.2	2.9	0.0	76.9	17.4	2.6	0.1	0.0	0.0	0.0	0.0	0.0
Light	98	0.0	3.0	51.7	14.2	2.7	0.1	16.7	10.8	0.2	0.0	0.5	0.0	0.0	0.0	0.0
Light	99	0.0	0.4	5.0	2.2	1.9	0.4	0.4	3.2	3.8	0.3	81.3	1.1	0.0	0.0	0.0
Light	100	0.0	0.0	2.0	9.2	8.6	0.0	27.4	1.9	44.3	6.5	0.0	0.1	0.0	0.0	0.0
Light	101	0.0	0.0	0.0	0.1	0.1	0.0	89.2	10.6	0.0	0.0	0.0	0.0	0.0	0.0	0.0
Light	102	0.1	5.8	29.9	16.9	9.1	0.6	9.6	18.1	9.5	0.5	0.0	0.0	0.0	0.0	0.0
Light	103	0.0	0.0	0.0	4.7	2.6	0.0	84.5	8.0	0.1	0.0	0.0	0.0	0.0	0.0	0.0
Light	104	0.0	0.0	0.0	3.3	0.5	0.0	92.5	3.4	0.3	0.0	0.0	0.0	0.0	0.0	0.0
Light	105	0.0	0.0	0.0	9.5	1.9	0.0	41.3	46.4	0.9	0.0	0.0	0.0	0.0	0.0	0.0
Light	106	0.0	0.0	0.0	0.2	0.2	0.0	91.3	7.9	0.3	0.0	0.0	0.0	0.0	0.0	0.0
Light	107	0.0	0.0	0.0	88.6	4.6	0.0	6.4	0.3	0.0	0.0	0.0	0.0	0.0	0.0	0.0
Light	108	0.0	0.0	0.0	0.9	4.1	0.0	46.9	38.3	9.7	0.2	0.0	0.0	0.0	0.0	0.0
Light	109	0.0	0.1	8.9	14.0	9.5	0.0	2.2	27.6	36.5	1.0	0.2	0.0	0.0	0.0	0.0
Light	110	0.0	0.0	0.0	3.1	9.9	0.0	8.7	12.1	56.9	9.0	0.0	0.0	0.3	0.0	0.0
Light	111	0.0	0.0	0.0	11.2	25.6	0.0	48.3	5.8	3.0	0.0	5.8	0.1	0.0	0.0	0.0
Light	112	0.0	5.4	55.4	35.1	1.5	0.0	0.9	0.2	0.0	0.0	1.5	0.0	0.0	0.0	0.0
Light	113	0.0	0.0	0.0	0.2	1.4	0.0	36.7	60.9	0.9	0.0	0.0	0.0	0.0	0.0	0.0
Light	114	0.0	0.0	1.4	20.3	3.5	0.0	41.8	31.7	1.3	0.0	0.0	0.0	0.0	0.0	0.0
Light	115	0.0	0.0	0.2	9.5	11.2	0.0	7.7	7.7	29.0	26.2	1.3	2.1	4.6	0.5	0.0
Light	116	0.0	2.9	18.1	12.6	11.2	18.2	19.2	0.7	3.7	0.3	11.9	1.0	0.0	0.0	0.0
Light	117	0.0	0.0	0.8	9.1	8.4	0.1	6.8	21.6	15.0	9.1	8.3	8.0	9.1	3.0	0.7
Light	118	0.0	0.1	17.7	1.1	14.7	6.2	1.0	10.6	34.7	4.5	9.3	0.1	0.0	0.0	0.0
Light	119	25.1	67.0	5.7	0.2	0.0	1.0	0.0	0.0	0.0	0.0	1.1	0.0	0.0	0.0	0.0
Light	120	0.0	0.0	0.0	63.0	26.3	0.0	7.1	1.9	0.1	0.0	1.7	0.0	0.0	0.0	0.0
Light	121	0.0	0.0	0.7	0.4	1.4	0.0	0.0	0.6	2.4	7.3	26.5	38.8	14.5	7.0	0.5
Light	122	0.0	0.0	0.0	16.8	15.7	0.0	38.3	21.6	6.7	0.8	0.0	0.0	0.0	0.0	0.0
Light	123	0.0	0.0	0.0	0.0	0.0	0.0	57.0	42.0	0.8	0.2	0.0	0.0	0.0	0.0	0.0
Light	124	0.0	0.0	0.0	0.0	0.0	0.0	93.5	6.5	0.0	0.0	0.0	0.0	0.0	0.0	0.0
Light	125	0.0	0.0	0.0	0.3	0.6	0.0	87.8	11.2	0.0	0.0	0.0	0.0	0.0	0.0	0.0
Light	126	0.0	0.0	0.0	0.0	0.9	0.0	34.6	61.3	3.2	0.0	0.0	0.0	0.0	0.0	0.0
Light	127	0.0	0.0	0.4	10.1	7.0	0.0	67.2	14.2	0.8	0.3	0.0	0.0	0.0	0.0	0.0
Light	128	0.0	0.0	1.9	20.0	11.0	0.0	58.7	8.5	0.0	0.0	0.0	0.0	0.0	0.0	0.0
Light	129	0.0	4.1	29.7	22.1	0.0	17.7	26.4	0.0	0.0	0.0	0.0	0.0	0.0	0.0	0.0
Light	130	0.0	0.0	0.0	0.7	0.5	0.0	41.7	44.0	9.8	3.3	0.0	0.0	0.0	0.0	0.0
Light	131	0.0	19.8	75.3	4.7	0.0	0.2	0.0	0.0	0.0	0.0	0.0	0.0	0.0	0.0	0.0
Light	132	0.0	14.2	10.6	9.7	8.5	35.7	2.2	7.0	5.4	2.0	2.5	0.8	0.8	0.6	0.0
Light	133	0.0	0.0	0.2	66.8	3.9	0.0	25.4	3.6	0.0	0.0	0.0	0.0	0.0	0.0	0.0
Light	134	0.0	0.0	0.0	0.7	2.4	0.0	12.5	49.2	33.5	1.8	0.0	0.0	0.0	0.0	0.0
Light	135	0.0	0.0	0.0	14.0	1.7	0.0	34.0	2.4	1.6	5.0	0.0	0.0	1.6	21.4	18.2
Light	136	0.0	0.0	0.0	0.0	0.0	0.0	10.9	2.0	5.9	12.4	0.0	3.1	37.1	16.9	11.6
Light	137	0.0	0.0	0.1	56.6	35.7	0.0	2.3	0.0	0.1	0.0	5.2	0.0	0.0	0.0	0.0
Light	138	0.0	0.0	6.2	76.8	17.0	0.0	0.0	0.0	0.0	0.0	0.0	0.0	0.0	0.0	0.0

Table S3: Distributions (%) of the unperturbed and RRS simulations of VVD Dark and Light states among 15 clusters.

		Cluster														
Type	Res	1	2	3	4	5	6	7	8	9	10	11	12	13	14	15
Light	139	0.0	0.0	0.1	26.7	11.0	0.0	51.7	8.6	1.9	0.0	0.0	0.0	0.0	0.0	0.0
Light	140	0.0	0.0	0.0	6.6	5.0	0.0	29.4	45.4	13.2	0.4	0.0	0.0	0.0	0.0	0.0
Light	141	0.0	10.9	49.5	14.8	3.1	1.4	7.5	9.9	2.3	0.0	0.6	0.0	0.0	0.0	0.0
Light	142	0.0	0.0	0.0	39.0	1.1	0.0	59.1	0.7	0.0	0.0	0.0	0.0	0.0	0.0	0.0
Light	143	0.0	0.0	0.0	9.3	0.4	0.0	80.7	8.4	1.1	0.0	0.0	0.0	0.0	0.0	0.0
Light	144	0.0	0.0	0.1	68.3	11.9	0.0	18.6	1.1	0.0	0.0	0.0	0.0	0.0	0.0	0.0
Light	145	0.0	0.0	0.0	11.6	5.8	0.0	14.6	2.8	19.4	15.2	3.0	26.1	1.6	0.0	0.0
Light	146	0.0	0.0	0.0	0.4	12.7	0.0	5.0	43.5	38.3	0.2	0.0	0.0	0.0	0.0	0.0
Light	147	0.0	52.4	45.9	0.3	0.0	1.3	0.0	0.0	0.0	0.0	0.0	0.0	0.0	0.0	0.0
Light	148	0.0	0.0	0.0	0.0	9.9	0.0	4.7	59.6	25.6	0.2	0.0	0.0	0.0	0.0	0.0

Table S4: Heat maps, histograms of Cα cross-correlation matrices for all residues in VVD from RRS for both Dark and Light states. Each residue number represents a simulation in which that particular residue is subjected to rigid body constraints.

	Heat Map[a]		Histogram[b]	
Residue	Dark	Light	Dark	Light
0	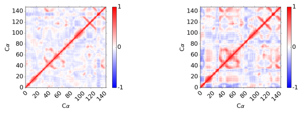		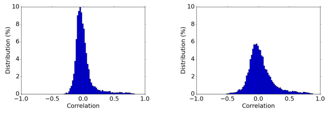	
1	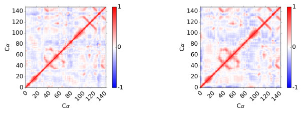		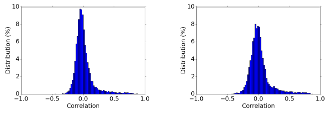	
2	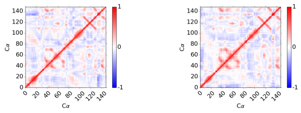		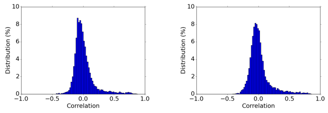	
3	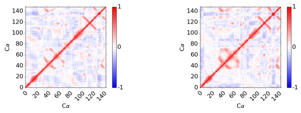		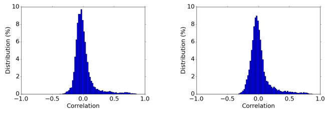	
4	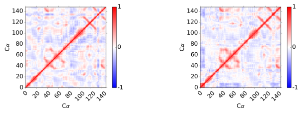		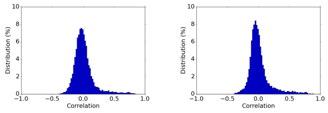	
5	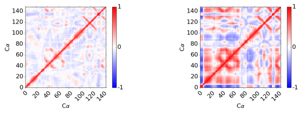		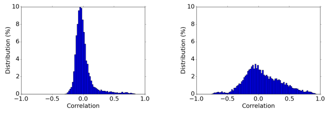	
6	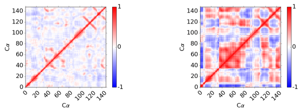		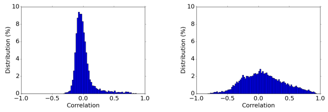	
7	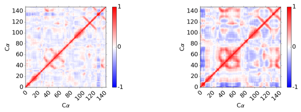		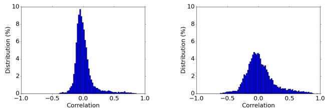	
8	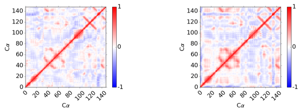		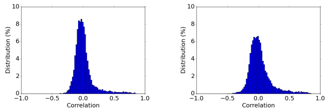	
9	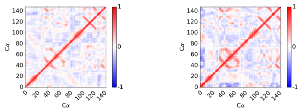		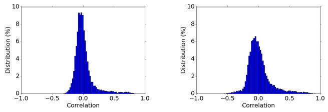	
10	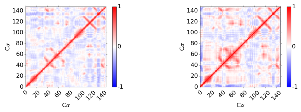		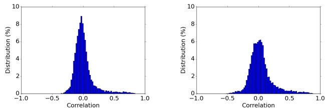	

[a] In these heat maps, red means positive correlation, blue means negative correlation, white means no correlation.
[b] In the histogram plots, normalized distribution is plotted using 0.2 as bin width.

Table S4: Heat maps, histograms of Cα cross-correlation matrices for all residues in VVD from RRS for both Dark and Light states. Each residue number represents a simulation in which that particular residue is subjected to rigid body constraints.

	Heat Map[a]		Histogram[b]	
Residue	Dark	Light	Dark	Light
11	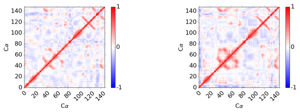		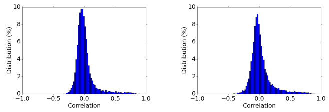	
12	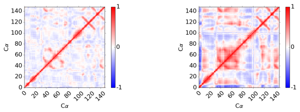		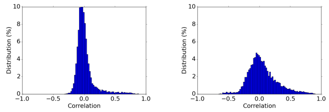	
13	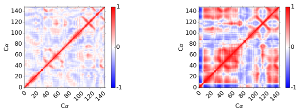		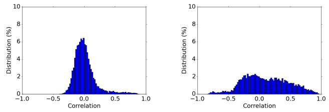	
14	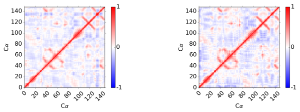		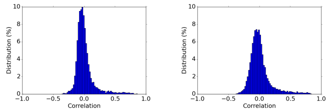	
15	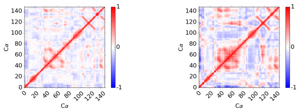		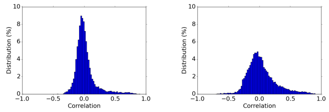	
16	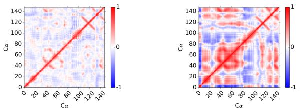		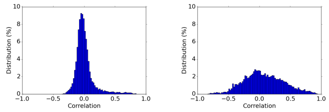	
17	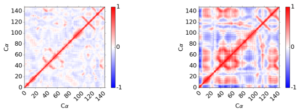		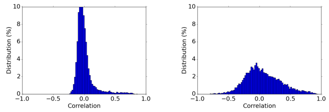	
18	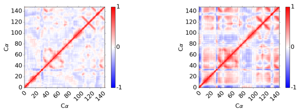		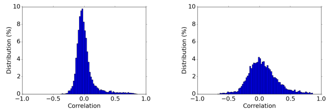	
19	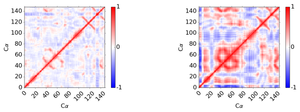		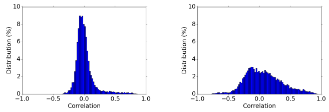	
20	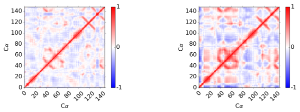		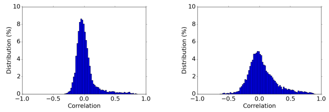	
21	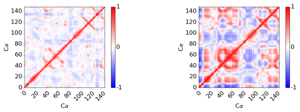		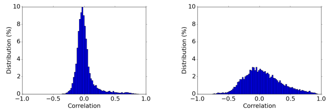	

[a] In these heat maps, red means positive correlation, blue means negative correlation, white means no correlation.
[b] In the histogram plots, normalized distribution is plotted using 0.2 as bin width.

Table S4: Heat maps, histograms of Cα cross-correlation matrices for all residues in VVD from RRS for both Dark and Light states. Each residue number represents a simulation in which that particular residue is subjected to rigid body constraints.

	Heat Map[a]		Histogram[b]	
Residue	Dark	Light	Dark	Light
22	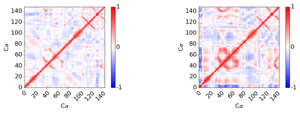		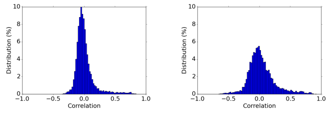	
23	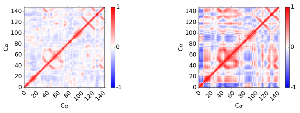		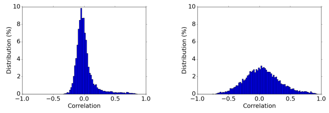	
24	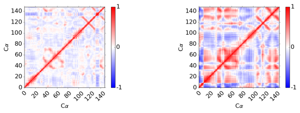		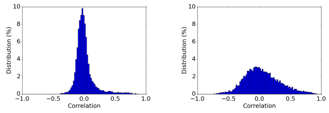	
25	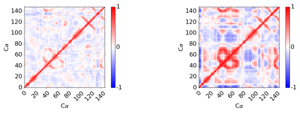		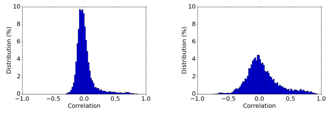	
26	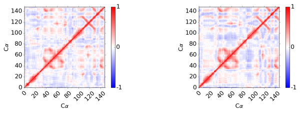		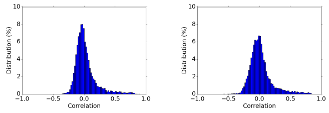	
27	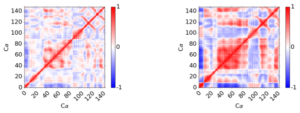		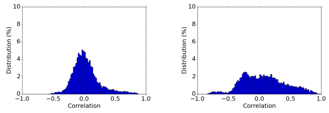	
28	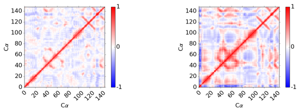		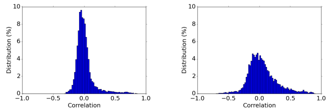	
29	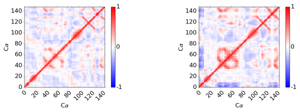		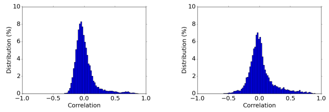	
30	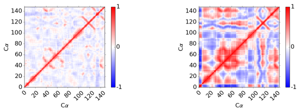		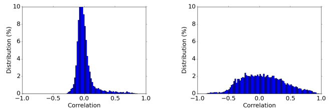	
31	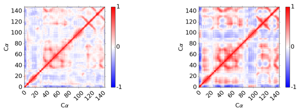		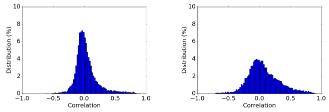	
32	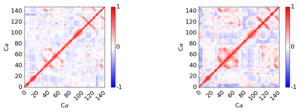		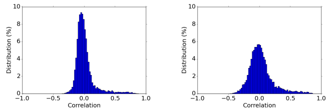	

[a] In these heat maps, red means positive correlation, blue means negative correlation, white means no correlation.
[b] In the histogram plots, normalized distribution is plotted using 0.2 as bin width.

Table S4: Heat maps, histograms of Cα cross-correlation matrices for all residues in VVD from RRS for both Dark and Light states. Each residue number represents a simulation in which that particular residue is subjected to rigid body constraints.

	Heat Map[a]		Histogram[b]	
Residue	Dark	Light	Dark	Light
33	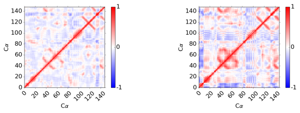		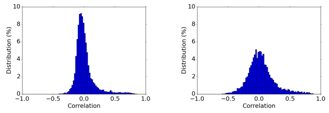	
34	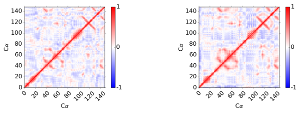		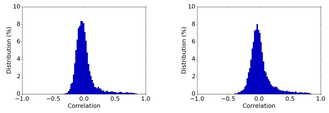	
35	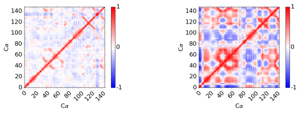		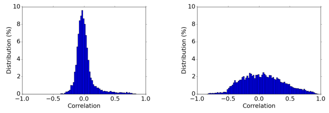	
36	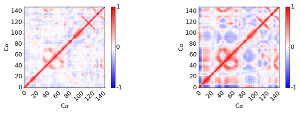		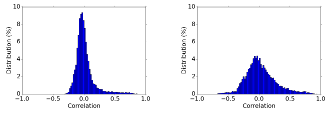	
37	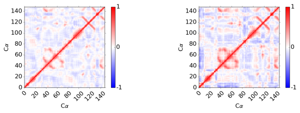		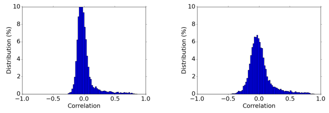	
38	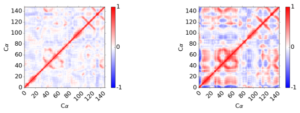		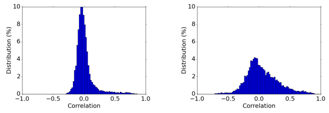	
39	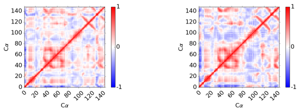		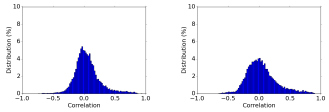	
40	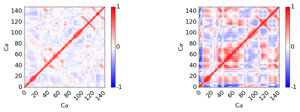		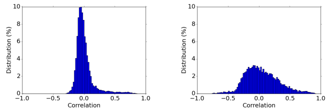	
41	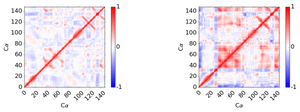		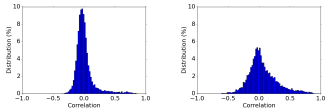	
42	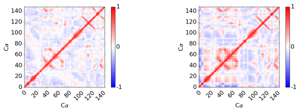		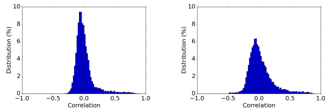	
43	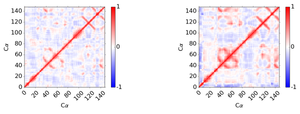		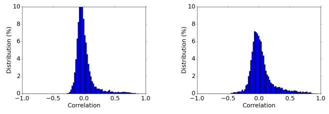	

[a] In these heat maps, red means positive correlation, blue means negative correlation, white means no correlation.
[b] In the histogram plots, normalized distribution is plotted using 0.2 as bin width.

Table S4: Heat maps, histograms of Cα cross-correlation matrices for all residues in VVD from RRS for both Dark and Light states. Each residue number represents a simulation in which that particular residue is subjected to rigid body constraints.

	Heat Map[a]		Histogram[b]	
Residue	Dark	Light	Dark	Light
44	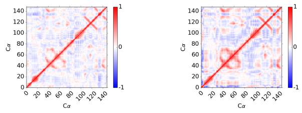		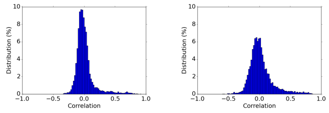	
45	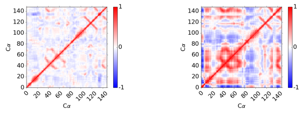		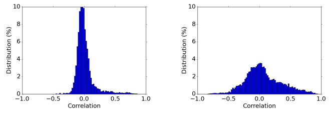	
46	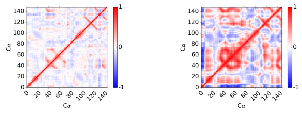		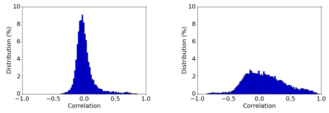	
47	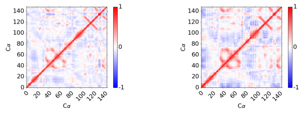		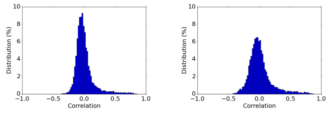	
48	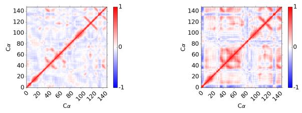		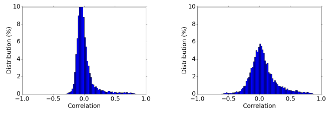	
49	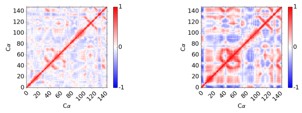		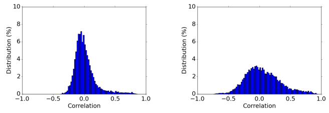	
50	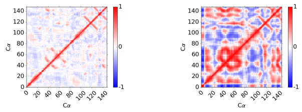		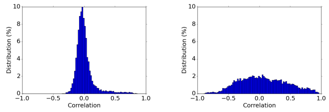	
51	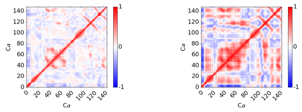		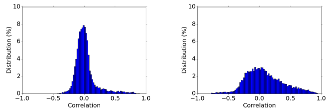	
52	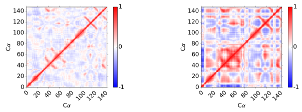		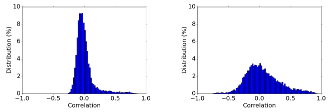	
53	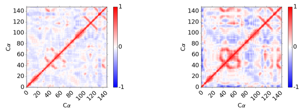		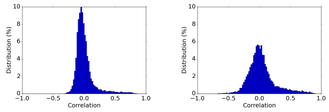	
54	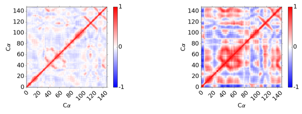		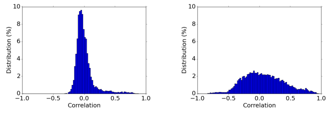	

[a] In these heat maps, red means positive correlation, blue means negative correlation, white means no correlation.
[b] In the histogram plots, normalized distribution is plotted using 0.2 as bin width.

Table S4: Heat maps, histograms of Cα cross-correlation matrices for all residues in VVD from RRS for both Dark and Light states. Each residue number represents a simulation in which that particular residue is subjected to rigid body constraints.

Residue	Heat Map[a] (Dark, Light)	Histogram[b] (Dark, Light)
55	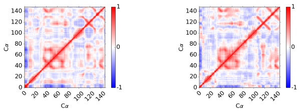	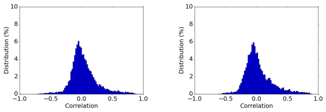
56	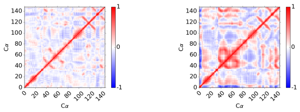	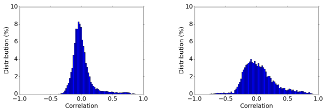
57	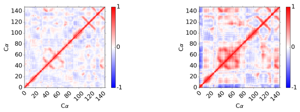	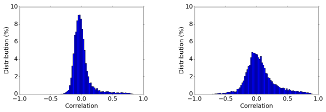
58	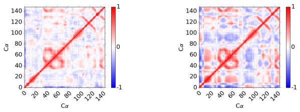	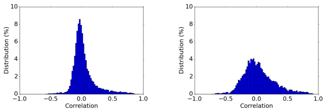
59	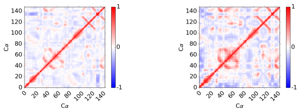	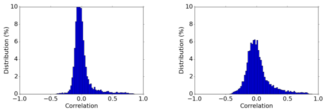
60	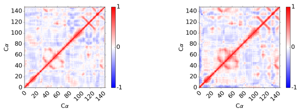	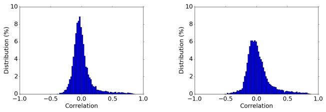
61	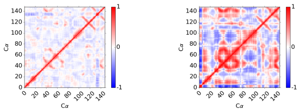	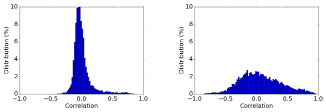
62	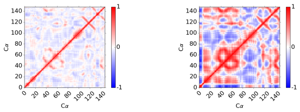	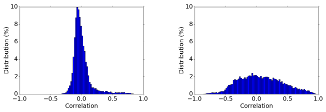
63	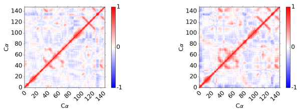	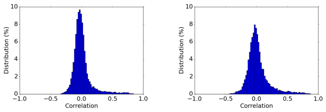
64	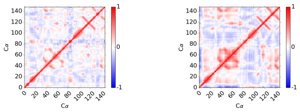	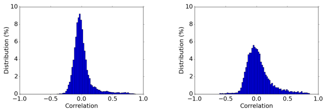
65	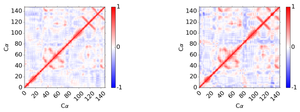	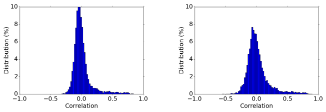

[a] In these heat maps, red means positive correlation, blue means negative correlation, white means no correlation.
[b] In the histogram plots, normalized distribution is plotted using 0.2 as bin width.

Table S4: Heat maps, histograms of Cα cross-correlation matrices for all residues in VVD from RRS for both Dark and Light states. Each residue number represents a simulation in which that particular residue is subjected to rigid body constraints.

Residue	Heat Map[a] Dark	Heat Map[a] Light	Histogram[b] Dark	Histogram[b] Light
66	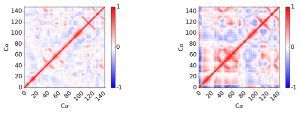		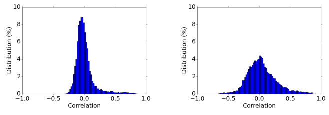	
67	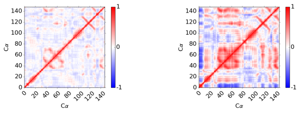		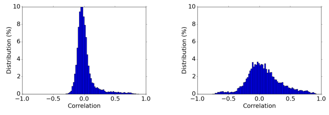	
68	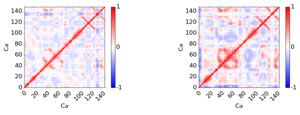		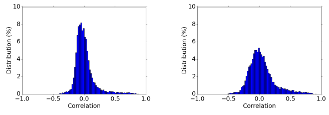	
69	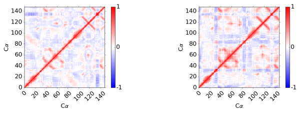		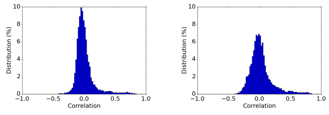	
70	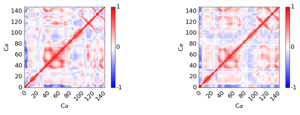		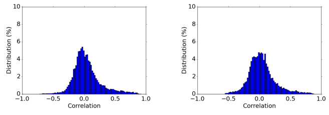	
71	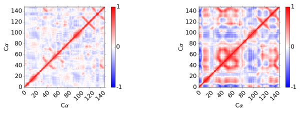		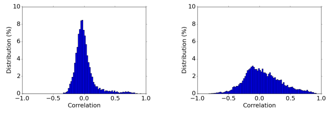	
72	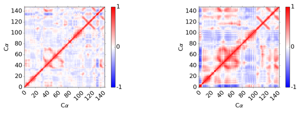		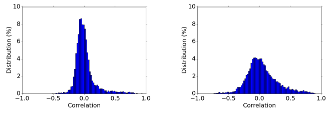	
73	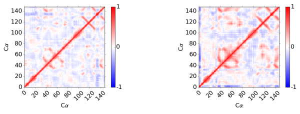		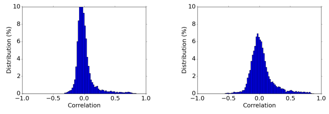	
74	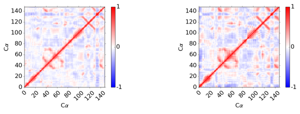		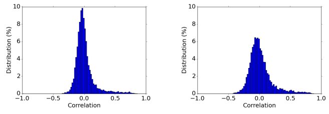	
75	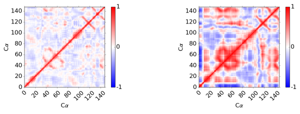		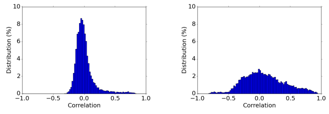	
76	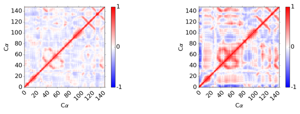		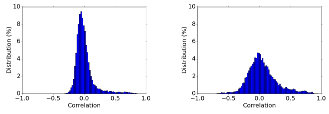	

[a] In these heat maps, red means positive correlation, blue means negative correlation, white means no correlation.
[b] In the histogram plots, normalized distribution is plotted using 0.2 as bin width.

Table S4: Heat maps, histograms of Cα cross-correlation matrices for all residues in VVD from RRS for both Dark and Light states. Each residue number represents a simulation in which that particular residue is subjected to rigid body constraints.

	Heat Map[a]		Histogram[b]	
Residue	Dark	Light	Dark	Light
77	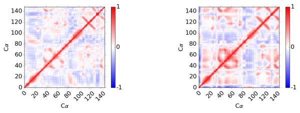		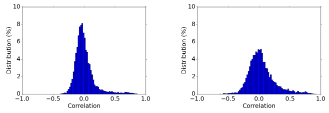	
78	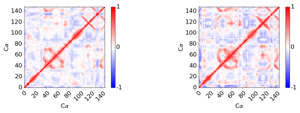		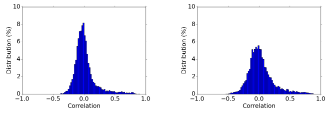	
79	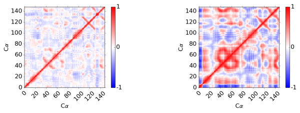		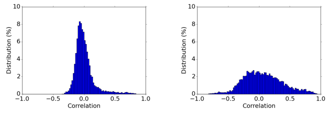	
80	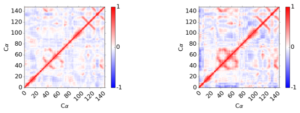		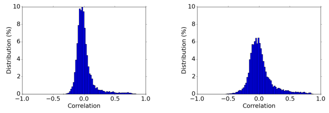	
81	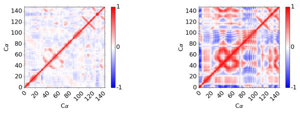		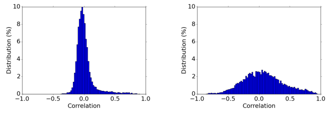	
82	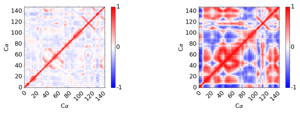		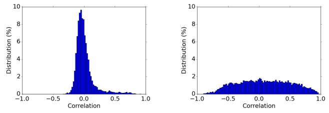	
83	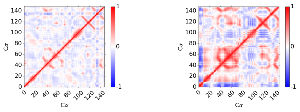		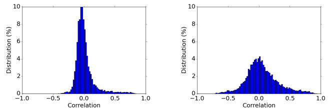	
84	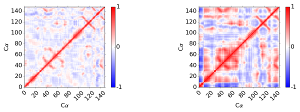		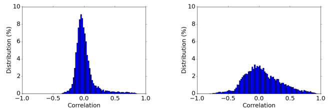	
85	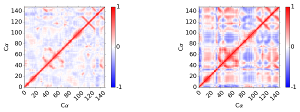		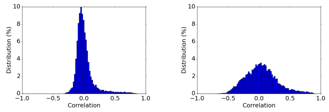	
86	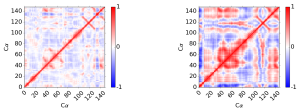		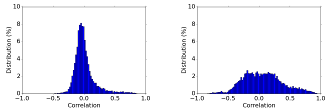	
87	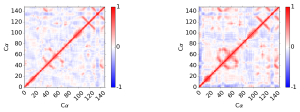		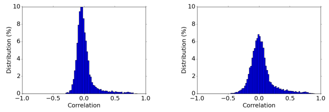	

[a] In these heat maps, red means positive correlation, blue means negative correlation, white means no correlation.
[b] In the histogram plots, normalized distribution is plotted using 0.2 as bin width.

Table S4: Heat maps, histograms of Cα cross-correlation matrices for all residues in VVD from RRS for both Dark and Light states. Each residue number represents a simulation in which that particular residue is subjected to rigid body constraints.

	Heat Map[a]		Histogram[b]	
Residue	Dark	Light	Dark	Light
88	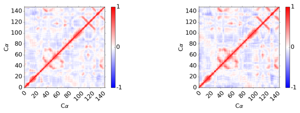		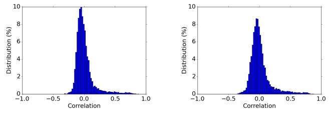	
89	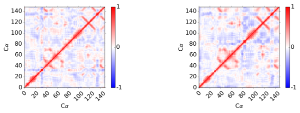		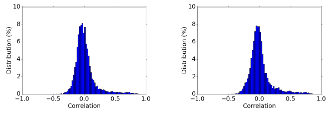	
90	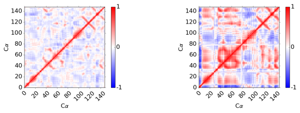		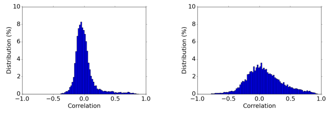	
91	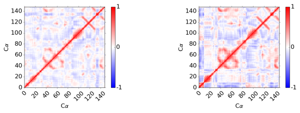		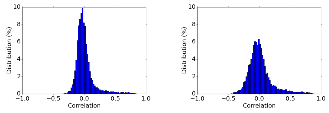	
92	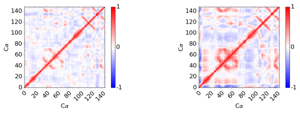		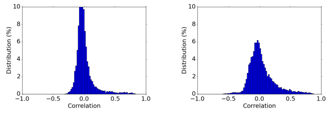	
93	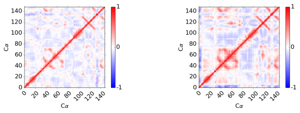		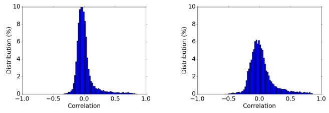	
94	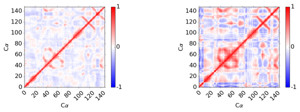		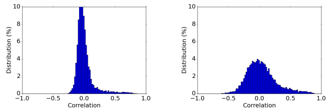	
95	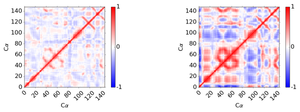		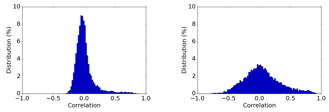	
96	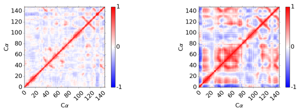		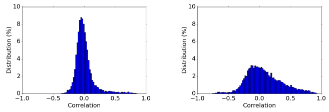	
97	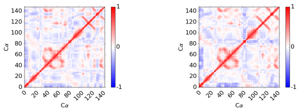		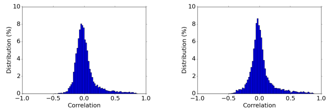	
98	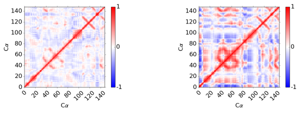		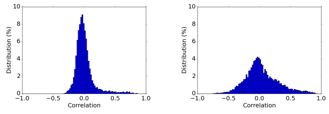	

[a] In these heat maps, red means positive correlation, blue means negative correlation, white means no correlation.
[b] In the histogram plots, normalized distribution is plotted using 0.2 as bin width.

Table S4: Heat maps, histograms of Cα cross-correlation matrices for all residues in VVD from RRS for both Dark and Light states. Each residue number represents a simulation in which that particular residue is subjected to rigid body constraints.

	Heat Map[a]		Histogram[b]	
Residue	Dark	Light	Dark	Light
99	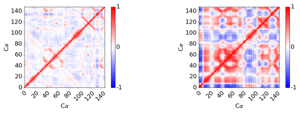		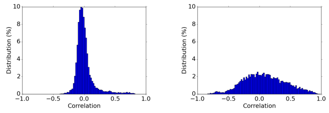	
100	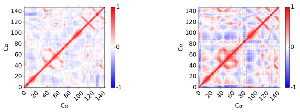		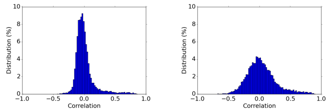	
101	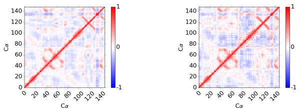		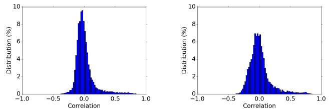	
102	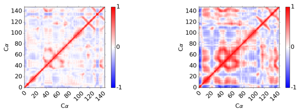		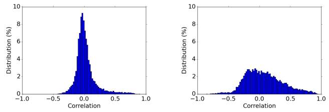	
103	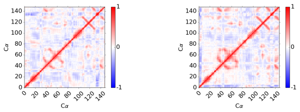		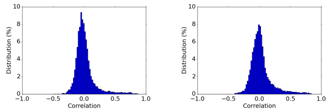	
104	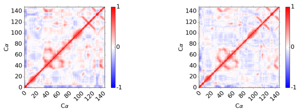		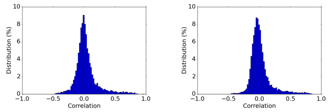	
105	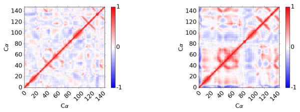		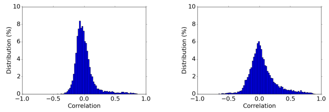	
106	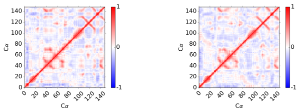		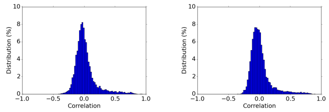	
107	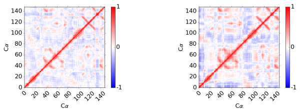		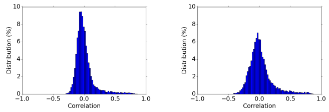	
108	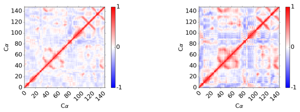		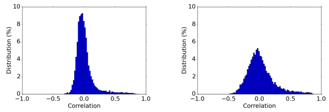	
109	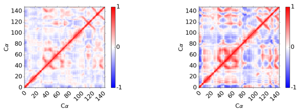		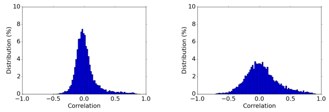	

[a] In these heat maps, red means positive correlation, blue means negative correlation, white means no correlation.
[b] In the histogram plots, normalized distribution is plotted using 0.2 as bin width.

Table S4: Heat maps, histograms of Cα cross-correlation matrices for all residues in VVD from RRS for both Dark and Light states. Each residue number represents a simulation in which that particular residue is subjected to rigid body constraints.

	Heat Map[a]		Histogram[b]	
Residue	Dark	Light	Dark	Light
110	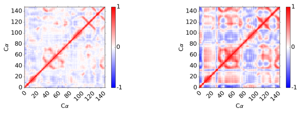		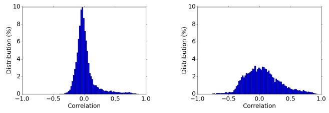	
111	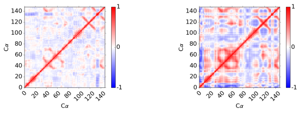		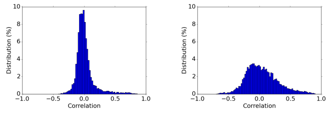	
112	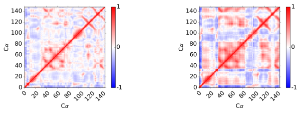		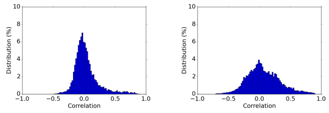	
113	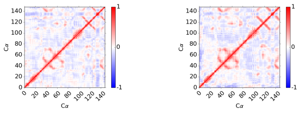		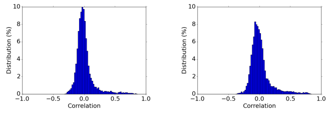	
114	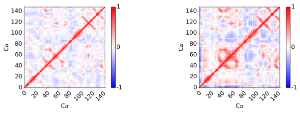		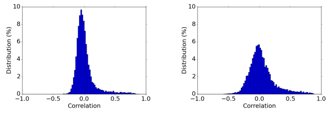	
115	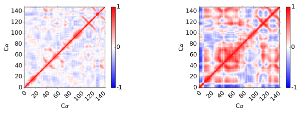		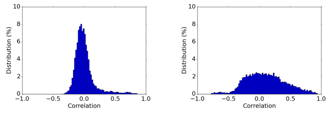	
116	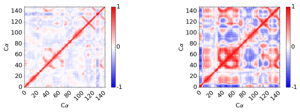		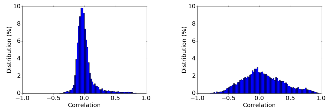	
117	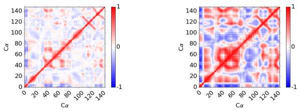		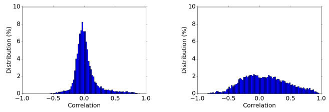	
118	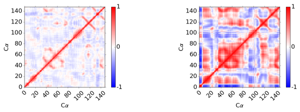		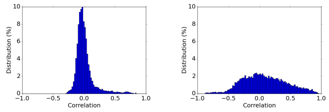	
119	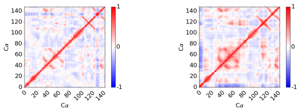		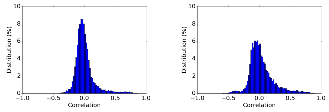	
120	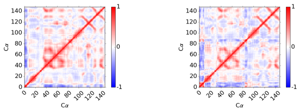		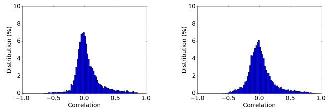	

[a] In these heat maps, red means positive correlation, blue means negative correlation, white means no correlation.
[b] In the histogram plots, normalized distribution is plotted using 0.2 as bin width.

Table S4: Heat maps, histograms of Cα cross-correlation matrices for all residues in VVD from RRS for both Dark and Light states. Each residue number represents a simulation in which that particular residue is subjected to rigid body constraints.

Residue	Heat Map[a] (Dark, Light)	Histogram[b] (Dark, Light)
121	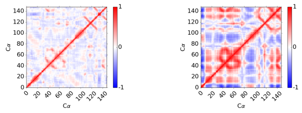	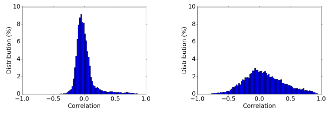
122	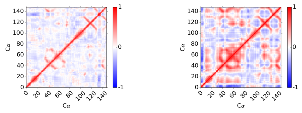	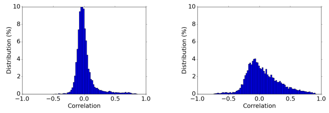
123	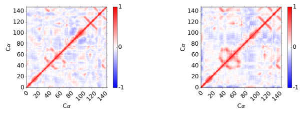	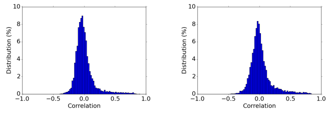
124	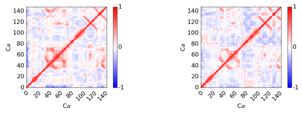	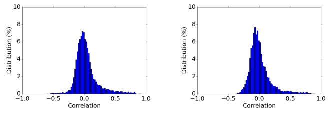
125	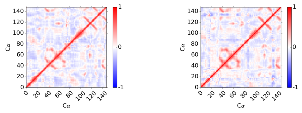	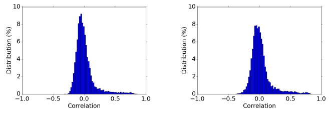
126	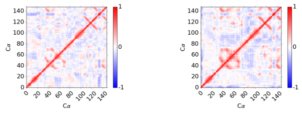	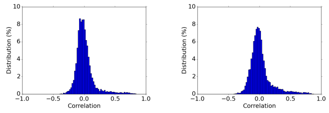
127	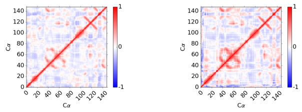	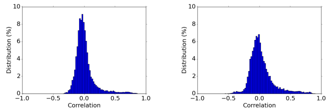
128	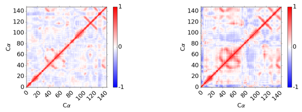	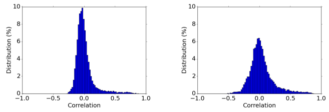
129	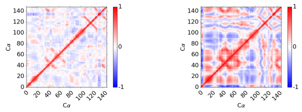	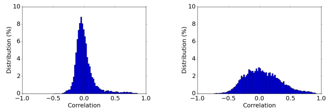
130	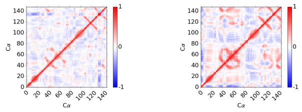	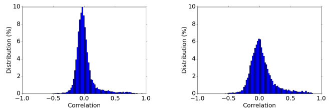
131	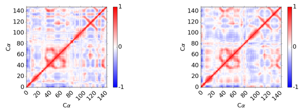	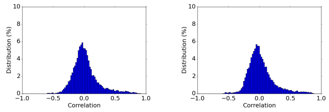

[a] In these heat maps, red means positive correlation, blue means negative correlation, white means no correlation.
[b] In the histogram plots, normalized distribution is plotted using 0.2 as bin width.

Table S4: Heat maps, histograms of Cα cross-correlation matrices for all residues in VVD from RRS for both Dark and Light states. Each residue number represents a simulation in which that particular residue is subjected to rigid body constraints.

	Heat Map[a]		Histogram[b]	
Residue	Dark	Light	Dark	Light
132	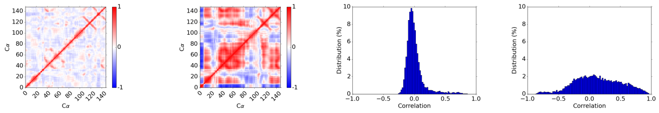			
133	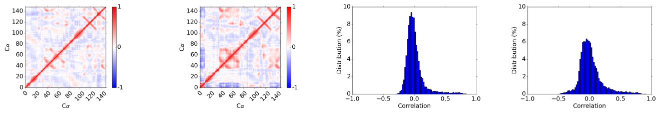			
134	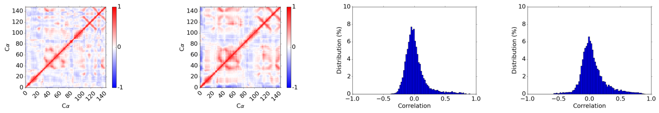			
135	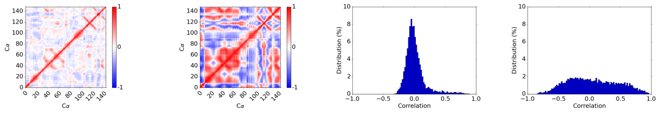			
136	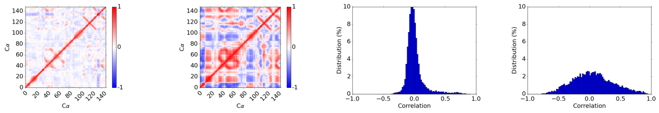			
137	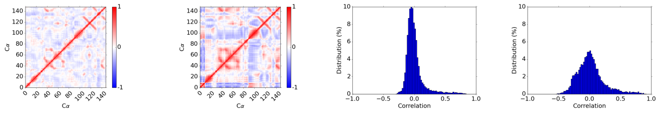			
138	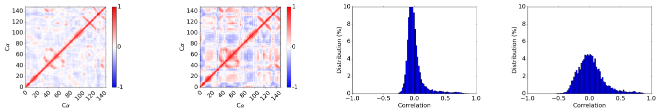			
139	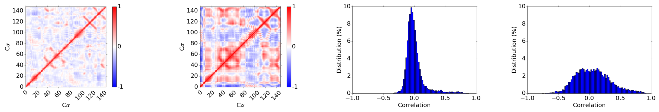			
140	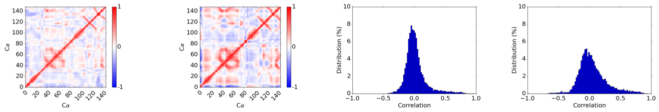			
141	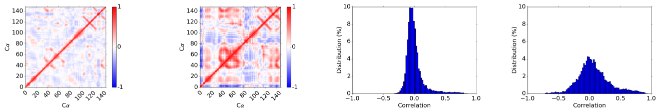			
142	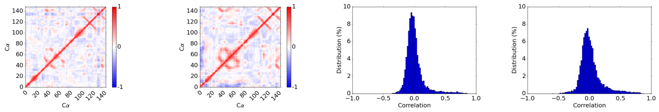			

[a] In these heat maps, red means positive correlation, blue means negative correlation, white means no correlation.
[b] In the histogram plots, normalized distribution is plotted using 0.2 as bin width.

Table S4: Heat maps, histograms of Cα cross-correlation matrices for all residues in VVD from RRS for both Dark and Light states. Each residue number represents a simulation in which that particular residue is subjected to rigid body constraints.

	Heat Map[a]		Histogram[b]	
Residue	Dark	Light	Dark	Light
143	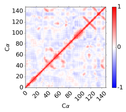	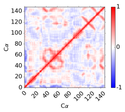	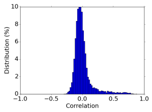	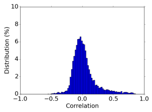
144	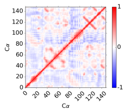	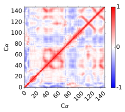	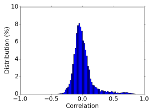	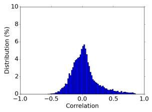
145	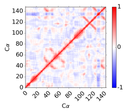	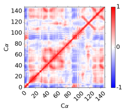	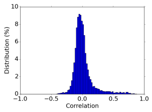	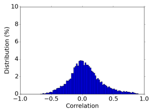
146	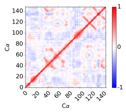	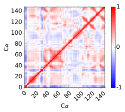	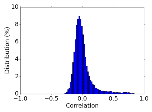	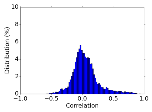
147	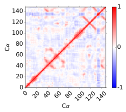	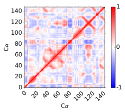	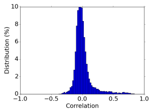	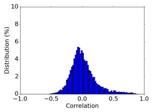
148	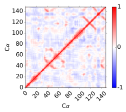	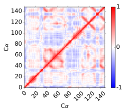	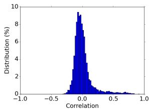	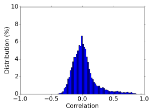

[a] In these heat maps, red means positive correlation, blue means negative correlation, white means no correlation.

[b] In the histogram plots, normalized distribution is plotted using 0.2 as bin width.

Table S5: Relative entropies (ΔS) and differences ($\Delta\Delta S$) of VVD between Dark and Light states. Entropy of VVD from unperturbed Dark state simulation is used as the reference value, and listed as zero. The Residue column represents simulations in which that particular residue is subjected to rigid body constraints. In Delta (Absolute) column, absolute difference between relative entropies of Dark and Light states are listed.

Residue	Dark ΔS	Light ΔS	Delta (Absolute) $\Delta\Delta S$
None	0.000	0.215	0.215
1	0.116	0.044	0.072
2	0.109	0.009	0.100
3	-0.052	-0.016	0.036
4	0.020	0.114	0.094
5	0.054	0.140	0.086
6	0.075	0.256	0.181
7	0.069	0.201	0.132
8	0.066	-0.016	0.082
9	0.025	-0.047	0.072
10	0.068	0.160	0.092
11	0.008	0.115	0.107
12	0.125	0.058	0.067
13	0.229	0.337	0.108
14	0.071	0.023	0.048
15	0.135	0.108	0.027
16	0.035	0.242	0.207
17	-0.011	0.162	0.173
18	0.082	0.122	0.040
19	0.006	0.156	0.150
20	0.161	0.175	0.014
21	-0.023	0.198	0.221
22	0.063	0.162	0.099
23	0.092	0.083	0.009
24	0.033	0.289	0.256
25	0.127	0.214	0.087
26	0.151	-0.019	0.170
27	0.205	0.217	0.012
28	0.004	0.027	0.023
29	0.163	0.192	0.029
30	-0.033	0.087	0.120
31	0.173	0.185	0.012
32	0.084	0.105	0.021
33	-0.022	0.128	0.150
34	0.138	0.086	0.052
35	0.045	0.187	0.142
36	0.119	0.058	0.061
37	-0.008	0.141	0.149
38	0.068	0.125	0.057
39	0.139	0.225	0.086
40	0.084	0.099	0.015
41	0.072	0.310	0.238
42	0.115	0.125	0.010
43	-0.042	0.145	0.187
44	0.064	0.083	0.019
45	0.013	0.166	0.153

Table S5: Relative entropies (ΔS) and differences ($\Delta\Delta S$) of VVD between Dark and Light states. Entropy of VVD from unperturbed Dark state simulation is used as the reference value, and listed as zero. The Residue column represents simulations in which that particular residue is subjected to rigid body constraints. In Delta (Absolute) column, absolute difference between relative entropies of Dark and Light states are listed.

Residue	Dark ΔS	Light ΔS	Delta (Absolute) $\Delta\Delta S$
46	0.124	0.181	0.057
47	0.084	0.062	0.022
48	0.032	0.212	0.180
49	0.078	0.223	0.145
50	0.133	0.156	0.023
51	0.100	0.228	0.128
52	0.070	0.207	0.137
53	0.045	0.205	0.160
54	-0.078	0.139	0.217
55	0.146	0.175	0.029
56	0.044	0.157	0.113
57	-0.076	0.183	0.259
58	0.178	0.173	0.005
59	0.017	0.098	0.081
60	0.006	0.119	0.113
61	0.041	0.287	0.246
62	0.021	0.200	0.179
63	0.068	0.052	0.016
64	0.092	0.116	0.024
65	0.138	0.224	0.086
66	-0.024	0.050	0.074
67	0.058	0.318	0.260
68	0.024	0.242	0.218
69	0.117	0.178	0.061
70	0.163	0.083	0.080
71	0.109	0.062	0.047
72	0.188	0.019	0.169
73	-0.012	0.126	0.138
74	-0.006	0.045	0.051
75	-0.035	0.281	0.316
76	-0.009	0.163	0.172
77	0.052	0.115	0.063
78	0.087	0.244	0.157
79	0.031	0.168	0.137
80	0.019	0.150	0.131
81	0.012	0.117	0.105
82	-0.017	0.277	0.294
83	-0.101	0.075	0.176
84	0.117	0.141	0.024
85	0.015	0.109	0.094
86	-0.001	0.246	0.247
87	0.012	0.117	0.105
88	0.008	0.056	0.048
89	0.130	0.049	0.081
90	0.136	0.236	0.100
91	0.003	0.111	0.108

Table S5: Relative entropies (ΔS) and differences ($\Delta\Delta S$) of VVD between Dark and Light states. Entropy of VVD from unperturbed Dark state simulation is used as the reference value, and listed as zero. The Residue column represents simulations in which that particular residue is subjected to rigid body constraints. In Delta (Absolute) column, absolute difference between relative entropies of Dark and Light states are listed.

Residue	Dark ΔS	Light ΔS	Delta (Absolute) $\Delta\Delta S$
92	0.026	0.136	0.110
93	0.015	0.131	0.116
94	0.007	0.158	0.151
95	0.109	0.142	0.033
96	0.146	0.223	0.077
97	0.188	0.087	0.101
98	0.029	0.260	0.231
99	-0.099	0.131	0.230
100	0.045	0.226	0.181
101	-0.064	-0.048	0.016
102	0.077	0.219	0.142
103	0.221	0.045	0.176
104	0.108	0.087	0.021
105	0.036	0.160	0.124
106	0.149	0.067	0.082
107	0.163	0.146	0.017
108	0.053	0.115	0.062
109	0.238	0.195	0.043
110	0.142	0.190	0.048
111	0.068	0.112	0.044
112	0.150	0.157	0.007
113	0.066	0.089	0.023
114	0.169	0.221	0.052
115	0.108	0.208	0.100
116	0.039	0.296	0.257
117	0.110	0.261	0.151
118	0.028	0.261	0.233
119	0.034	0.030	0.004
120	0.190	0.078	0.112
121	0.158	0.307	0.149
122	-0.002	0.079	0.081
123	-0.068	0.031	0.099
124	0.166	-0.059	0.225
125	0.047	0.196	0.149
126	0.026	0.054	0.028
127	0.122	0.131	0.009
128	0.102	0.127	0.025
129	0.006	0.199	0.193
130	0.022	0.227	0.205
131	0.207	0.164	0.043
132	0.040	0.266	0.226
133	0.057	0.092	0.035
134	0.172	0.132	0.040
135	0.033	0.212	0.179
136	0.012	0.274	0.262
137	-0.005	0.126	0.131

Table S5: Relative entropies (ΔS) and differences ($\Delta\Delta S$) of VVD between Dark and Light states. Entropy of VVD from unperturbed Dark state simulation is used as the reference value, and listed as zero. The Residue column represents simulations in which that particular residue is subjected to rigid body constraints. In Delta (Absolute) column, absolute difference between relative entropies of Dark and Light states are listed.

Residue	Dark ΔS	Light ΔS	Delta (Absolute) $\Delta\Delta S$
138	0.058	0.129	0.071
139	0.085	0.099	0.014
140	0.136	0.135	0.001
141	-0.122	0.202	0.324
142	0.158	0.008	0.150
143	-0.077	0.058	0.135
144	0.091	0.103	0.012
145	0.053	0.195	0.142
146	0.000	0.081	0.081
147	0.061	0.129	0.068
148	-0.012	0.035	0.047

Table S6: Relative entropies (ΔS) and differences ($\Delta\Delta S$) of VVD between Dark and Light states sorted with ascending order. Each column from Table S5 is sorted separately, therefore with separate residue list.

	Rigid Dark		Rigid Light		Delta Entropy(Absolute)	
#	Residue	ΔS	Residue	ΔS	Residue	$\Delta\Delta S$
1	141	-0.122	124	-0.059	140	0.001
2	83	-0.101	101	-0.048	119	0.004
3	99	-0.099	9	-0.047	58	0.005
4	54	-0.078	26	-0.019	112	0.007
5	143	-0.077	3	-0.016	127	0.009
6	57	-0.076	8	-0.016	23	0.009
7	123	-0.068	142	0.008	42	0.010
8	101	-0.064	2	0.009	144	0.012
9	3	-0.052	72	0.019	31	0.012
10	43	-0.042	14	0.023	27	0.012
11	75	-0.035	28	0.027	20	0.014
12	30	-0.033	119	0.030	139	0.014
13	66	-0.024	123	0.031	40	0.015
14	21	-0.023	148	0.035	63	0.016
15	33	-0.022	1	0.044	101	0.016
16	82	-0.017	103	0.045	107	0.017
17	73	-0.012	74	0.045	44	0.019
18	148	-0.012	89	0.049	104	0.021
19	17	-0.011	66	0.050	32	0.021
20	76	-0.009	63	0.052	47	0.022
21	37	-0.008	126	0.054	50	0.023
22	74	-0.006	88	0.056	28	0.023
23	137	-0.005	12	0.058	113	0.023
24	122	-0.002	36	0.058	64	0.024
25	86	-0.001	143	0.058	84	0.024
26	0	0.000	47	0.062	128	0.025
27	146	0.000	71	0.062	15	0.027
28	91	0.003	106	0.067	126	0.028
29	28	0.004	83	0.075	55	0.029
30	60	0.006	120	0.078	29	0.029
31	19	0.006	122	0.079	95	0.033
32	129	0.006	146	0.081	133	0.035
33	94	0.007	23	0.083	3	0.036
34	88	0.008	70	0.083	134	0.040
35	11	0.008	44	0.083	18	0.040
36	87	0.012	34	0.086	131	0.043
37	136	0.012	30	0.087	109	0.043
38	81	0.012	104	0.087	111	0.044
39	45	0.013	97	0.087	71	0.047
40	93	0.015	113	0.089	148	0.047
41	85	0.015	133	0.092	88	0.048
42	59	0.017	59	0.098	14	0.048
43	80	0.019	139	0.099	110	0.048
44	4	0.020	40	0.099	74	0.051
45	62	0.021	144	0.103	114	0.052
46	130	0.022	32	0.105	34	0.052
47	68	0.024	15	0.108	46	0.057
48	9	0.025	85	0.109	38	0.057

Table S6: Relative entropies (ΔS) and differences ($\Delta\Delta S$) of VVD between Dark and Light states sorted with ascending order. Each column from Table S5 is sorted separately, therefore with separate residue list.

	Rigid Dark		Rigid Light		Delta Entropy(Absolute)	
#	Residue	ΔS	Residue	ΔS	Residue	$\Delta\Delta S$
49	126	0.026	91	0.111	69	0.061
50	92	0.026	111	0.112	36	0.061
51	118	0.028	4	0.114	108	0.062
52	98	0.029	11	0.115	77	0.063
53	79	0.031	77	0.115	12	0.067
54	48	0.032	108	0.115	147	0.068
55	24	0.033	64	0.116	138	0.071
56	135	0.033	81	0.117	1	0.072
57	119	0.034	87	0.117	9	0.072
58	16	0.035	60	0.119	66	0.074
59	105	0.036	18	0.122	96	0.077
60	116	0.039	42	0.125	70	0.080
61	132	0.040	38	0.125	59	0.081
62	61	0.041	73	0.126	122	0.081
63	56	0.044	137	0.126	89	0.081
64	35	0.045	128	0.127	146	0.081
65	53	0.045	33	0.128	106	0.082
66	100	0.045	138	0.129	8	0.082
67	125	0.047	147	0.129	5	0.086
68	77	0.052	99	0.131	39	0.086
69	108	0.053	127	0.131	65	0.086
70	145	0.053	93	0.131	25	0.087
71	5	0.054	134	0.132	10	0.092
72	133	0.057	140	0.135	4	0.094
73	67	0.058	92	0.136	85	0.094
74	138	0.058	54	0.139	123	0.099
75	147	0.061	5	0.140	22	0.099
76	22	0.063	84	0.141	2	0.100
77	44	0.064	37	0.141	90	0.100
78	113	0.066	95	0.142	115	0.100
79	8	0.066	43	0.145	97	0.101
80	111	0.068	107	0.146	81	0.105
81	38	0.068	80	0.150	87	0.105
82	10	0.068	19	0.156	11	0.107
83	63	0.068	50	0.156	91	0.108
84	7	0.069	56	0.157	13	0.108
85	52	0.070	112	0.157	92	0.110
86	14	0.071	94	0.158	120	0.112
87	41	0.072	105	0.160	60	0.113
88	6	0.075	10	0.160	56	0.113
89	102	0.077	17	0.162	93	0.116
90	49	0.078	22	0.162	30	0.120
91	18	0.082	76	0.163	105	0.124
92	32	0.084	131	0.164	51	0.128
93	40	0.084	45	0.166	80	0.131
94	47	0.084	79	0.168	137	0.131
95	139	0.085	58	0.173	7	0.132
96	78	0.087	20	0.175	143	0.135

Table S6: Relative entropies (ΔS) and differences ($\Delta\Delta S$) of VVD between Dark and Light states sorted with ascending order. Each column from Table S5 is sorted separately, therefore with separate residue list.

	Rigid Dark		Rigid Light		Delta Entropy(Absolute)	
#	Residue	ΔS	Residue	ΔS	Residue	$\Delta\Delta S$
97	144	0.091	55	0.175	52	0.137
98	64	0.092	69	0.178	79	0.137
99	23	0.092	46	0.181	73	0.138
100	51	0.100	57	0.183	102	0.142
101	128	0.102	31	0.185	35	0.142
102	115	0.108	35	0.187	145	0.142
103	104	0.108	110	0.190	49	0.145
104	71	0.109	29	0.192	121	0.149
105	2	0.109	145	0.195	37	0.149
106	95	0.109	109	0.195	125	0.149
107	117	0.110	125	0.196	19	0.150
108	42	0.115	21	0.198	142	0.150
109	1	0.116	129	0.199	33	0.150
110	69	0.117	62	0.200	94	0.151
111	84	0.117	7	0.201	117	0.151
112	36	0.119	141	0.202	45	0.153
113	127	0.122	53	0.205	78	0.157
114	46	0.124	52	0.207	53	0.160
115	12	0.125	115	0.208	72	0.169
116	25	0.127	48	0.212	26	0.170
117	89	0.130	135	0.212	76	0.172
118	50	0.133	25	0.214	17	0.173
119	15	0.135	0	0.215	83	0.176
120	140	0.136	27	0.217	103	0.176
121	90	0.136	102	0.219	62	0.179
122	34	0.138	114	0.221	135	0.179
123	65	0.138	96	0.223	48	0.180
124	39	0.139	49	0.223	100	0.181
125	110	0.142	65	0.224	6	0.181
126	96	0.146	39	0.225	43	0.187
127	55	0.146	100	0.226	129	0.193
128	106	0.149	130	0.227	130	0.205
129	112	0.150	51	0.228	16	0.207
130	26	0.151	90	0.236	0	0.215
131	142	0.158	16	0.242	54	0.217
132	121	0.158	68	0.242	68	0.218
133	20	0.161	78	0.244	21	0.221
134	107	0.163	86	0.246	124	0.225
135	70	0.163	6	0.256	132	0.226
136	29	0.163	98	0.260	99	0.230
137	124	0.166	117	0.261	98	0.231
138	114	0.169	118	0.261	118	0.233
139	134	0.172	132	0.266	41	0.238
140	31	0.173	136	0.274	61	0.246
141	58	0.178	82	0.277	86	0.247
142	72	0.188	75	0.281	24	0.256
143	97	0.188	61	0.287	116	0.257
144	120	0.190	24	0.289	57	0.259

Table S6: Relative entropies (ΔS) and differences ($\Delta\Delta S$) of VVD between Dark and Light states sorted with ascending order. Each column from Table S5 is sorted separately, therefore with separate residue list.

	Rigid Dark		Rigid Light		Delta Entropy(Absolute)	
#	Residue	ΔS	Residue	ΔS	Residue	$\Delta\Delta S$
145	27	0.205	116	0.296	67	0.260
146	131	0.207	121	0.307	136	0.262
147	103	0.221	41	0.310	82	0.294
148	13	0.229	67	0.318	75	0.316
149	109	0.238	13	0.337	141	0.324

Table S7: Projections of all simulations onto the PC1 mode generated from principal component analysis (PCA) of the unperturbed Light state simulation with optimized Dark state structure as reference.)

<table>
<tr><th>Residue</th><th>Distribution</th><th>Residue</th><th>Distribution</th></tr>
<tr><td>None</td><td>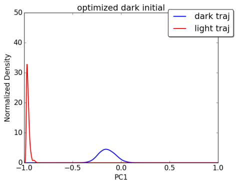
</td><td></td><td></td></tr>
<tr><td>1</td><td>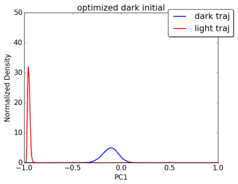
</td><td>2</td><td>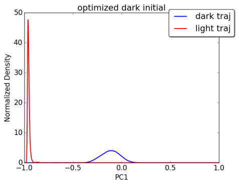
</td></tr>
<tr><td>3</td><td>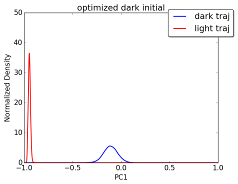
</td><td>4</td><td>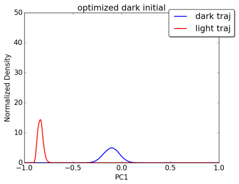
</td></tr>
<tr><td>5</td><td>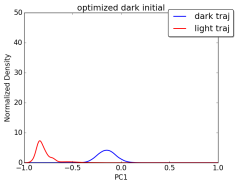
</td><td>6</td><td>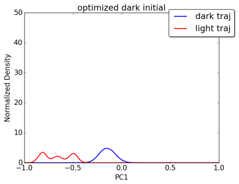
</td></tr>
<tr><td>7</td><td>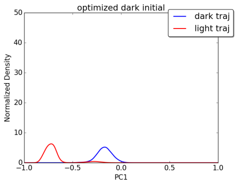
</td><td>8</td><td>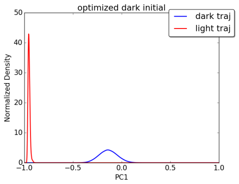
</td></tr>
<tr><td>9</td><td>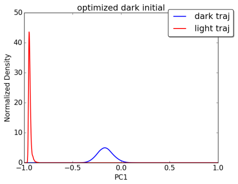
</td><td>10</td><td>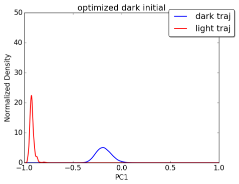
</td></tr>
<tr><td>11</td><td>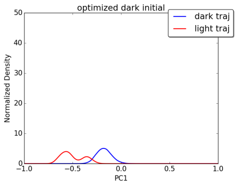
</td><td>12</td><td>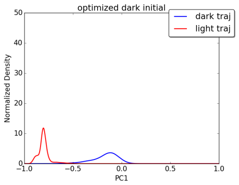
</td></tr>
<tr><td>13</td><td>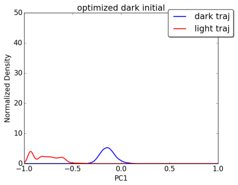
</td><td>14</td><td>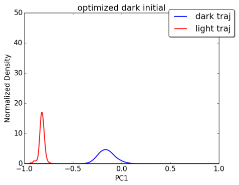
</td></tr>
</table>

Table S7: Projections of all simulations onto the PC1 mode generated from principal component analysis (PCA) of the unperturbed Light state simulation with optimized Dark state structure as reference.

Residue	Distribution	Residue	Distribution
15	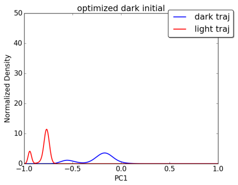	16	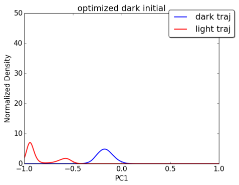
17	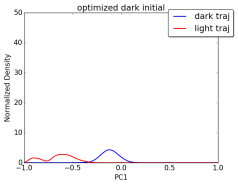	18	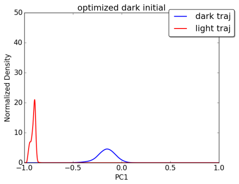
19	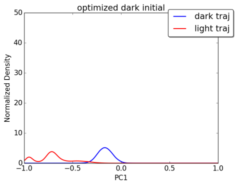	20	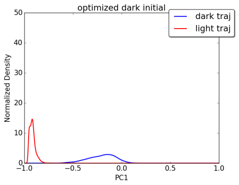
21	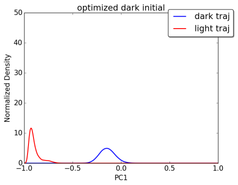	22	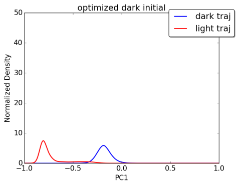
23	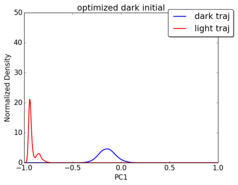	24	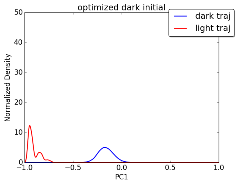
25	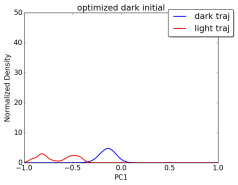	26	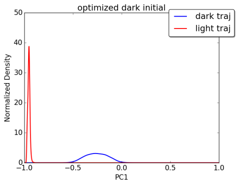
27	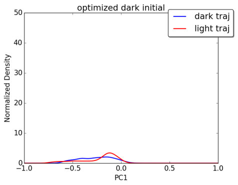	28	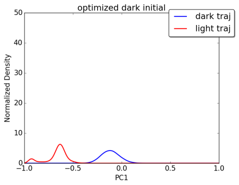
29	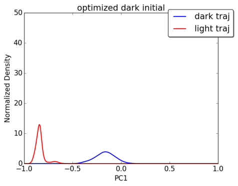	30	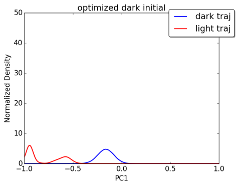

Table S7: Projections of all simulations onto the PC1 mode generated from principal component analysis (PCA) of the unperturbed Light state simulation with optimized Dark state structure as reference.

Residue	Distribution	Residue	Distribution
31	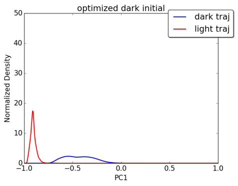	32	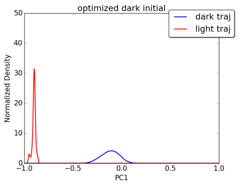
33	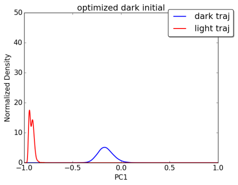	34	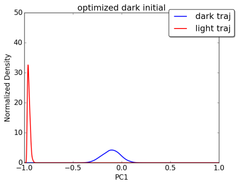
35	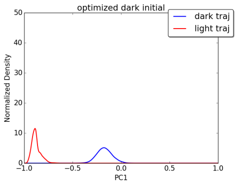	36	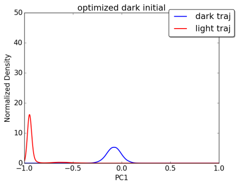
37	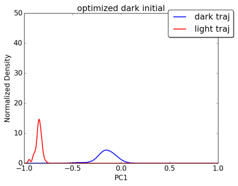	38	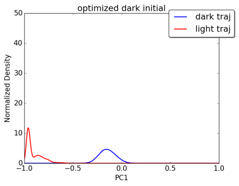
39	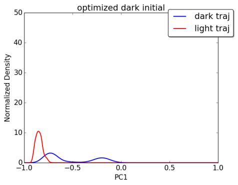	40	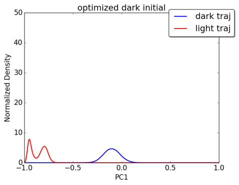
41	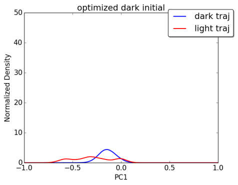	42	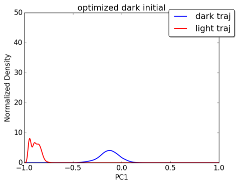
43	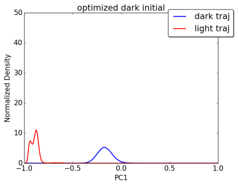	44	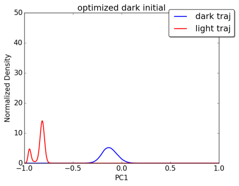
45	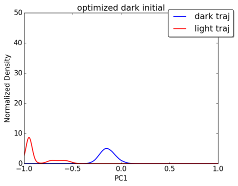	46	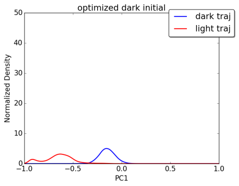

Table S7: Projections of all simulations onto the PC1 mode generated from principal component analysis (PCA) of the unperturbed Light state simulation with optimized Dark state structure as reference.

Residue	Distribution	Residue	Distribution
47	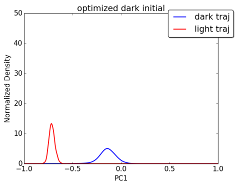	48	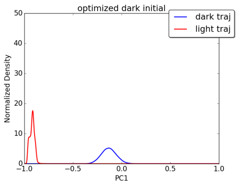
49	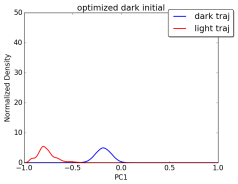	50	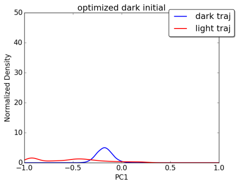
51	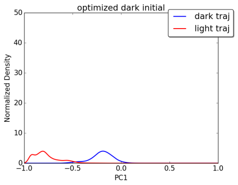	52	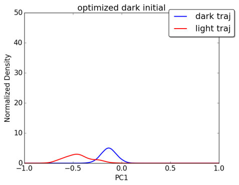
53	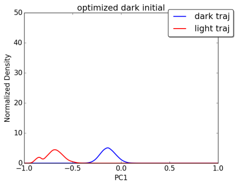	54	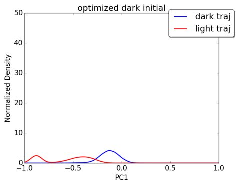
55	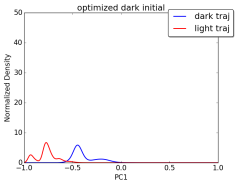	56	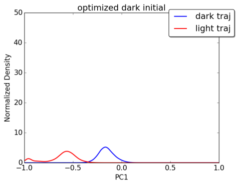
57	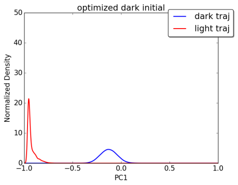	58	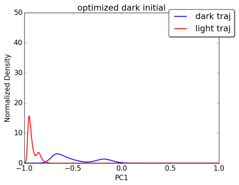
59	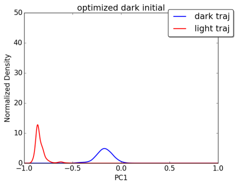	60	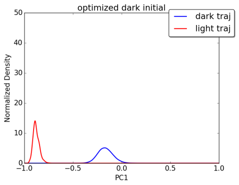
61	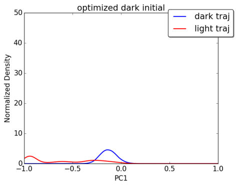	62	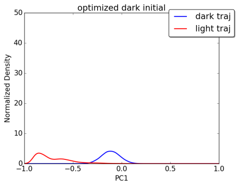

Table S7: Projections of all simulations onto the PC1 mode generated from principal component analysis (PCA) of the unperturbed Light state simulation with optimized Dark state structure as reference.

Residue	Distribution	Residue	Distribution
63	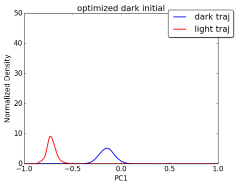	64	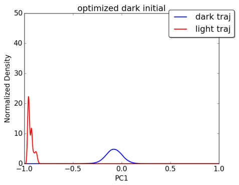
65	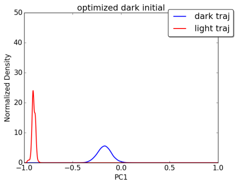	66	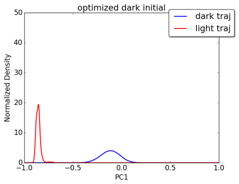
67	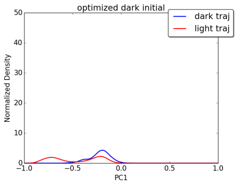	68	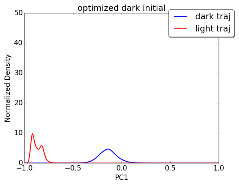
69	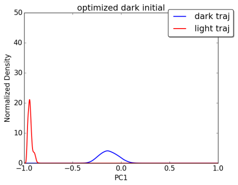	70	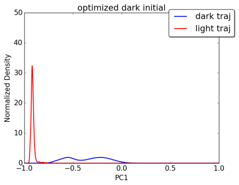
71	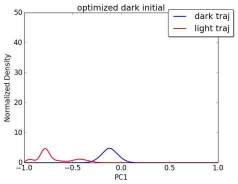	72	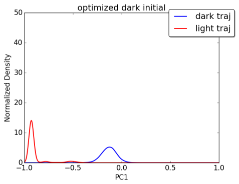
73	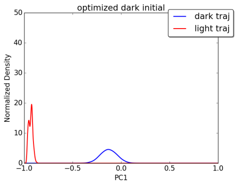	74	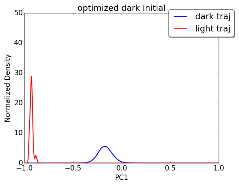
75	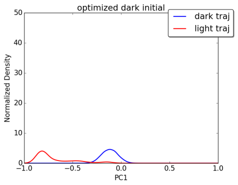	76	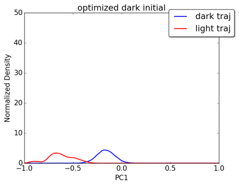
77	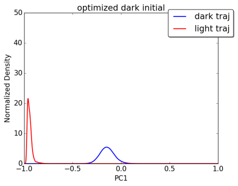	78	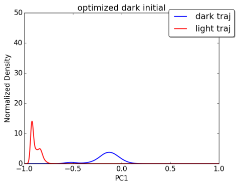

Table S7: Projections of all simulations onto the PC1 mode generated from principal component analysis (PCA) of the unperturbed Light state simulation with optimized Dark state structure as reference.

Residue	Distribution	Residue	Distribution
79	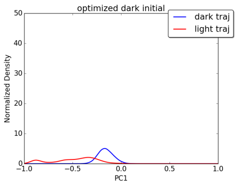	80	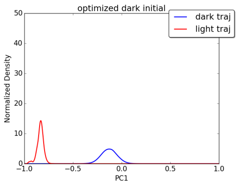
81	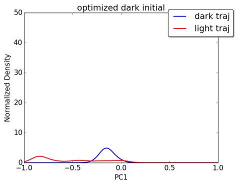	82	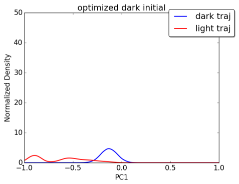
83	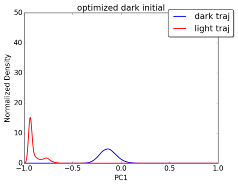	84	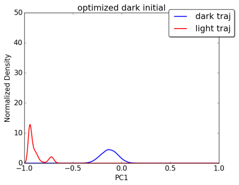
85	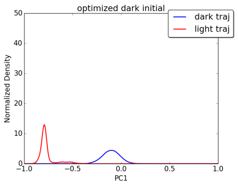	86	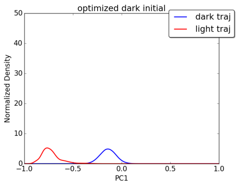
87	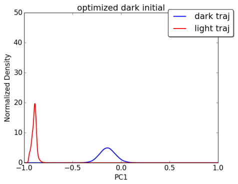	88	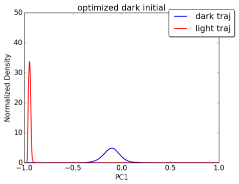
89	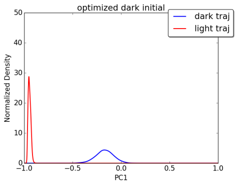	90	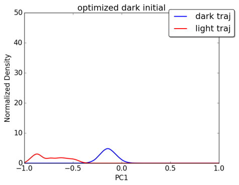
91	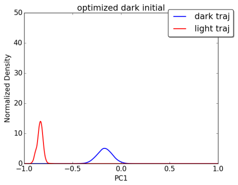	92	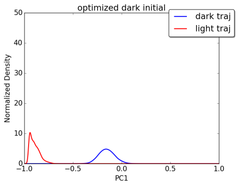
93	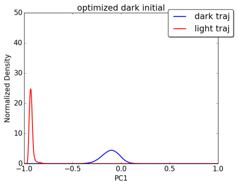	94	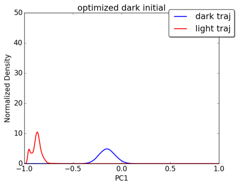

Table S7: Projections of all simulations onto the PC1 mode generated from principal component analysis (PCA) of the unperturbed Light state simulation with optimized Dark state structure as reference.

Residue	Distribution	Residue	Distribution
95	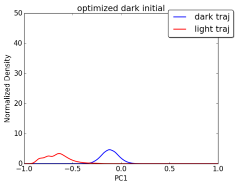	96	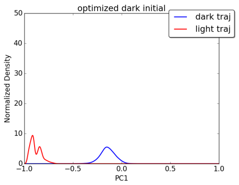
97	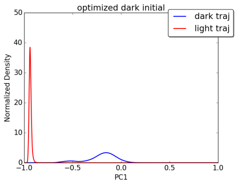	98	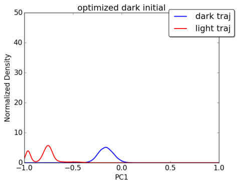
99	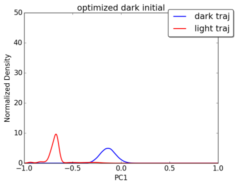	100	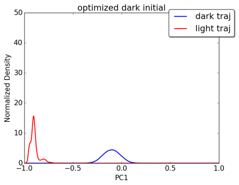
101	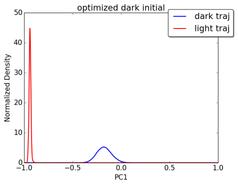	102	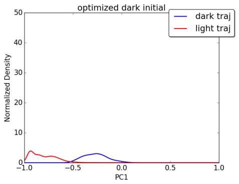
103	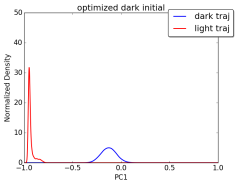	104	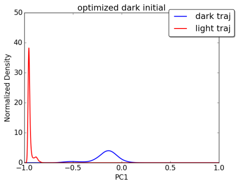
105	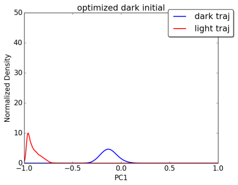	106	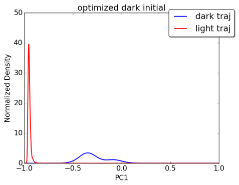
107	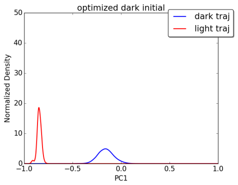	108	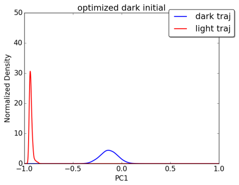
109	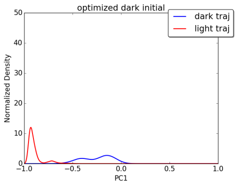	110	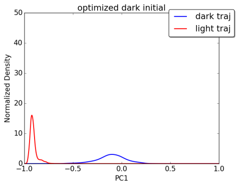

Table S7: Projections of all simulations onto the PC1 mode generated from principal component analysis (PCA) of the unperturbed Light state simulation with optimized Dark state structure as reference.

Residue	Distribution	Residue	Distribution
111	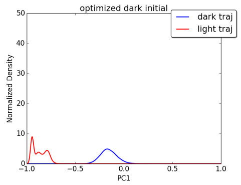	112	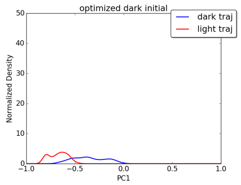
113	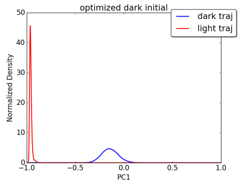	114	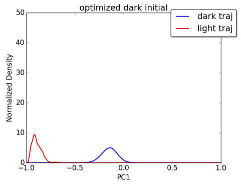
115	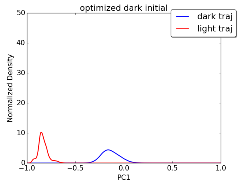	116	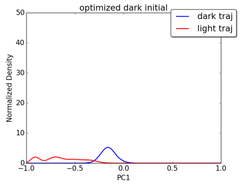
117	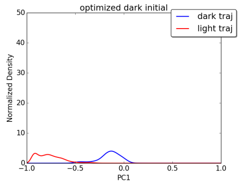	118	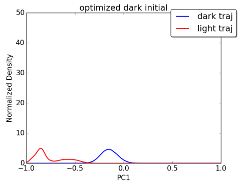
119	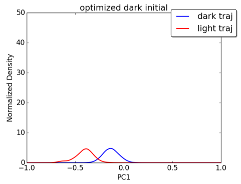	120	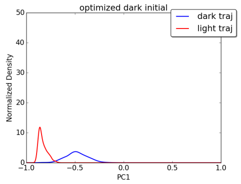
121	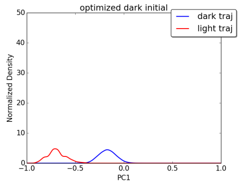	122	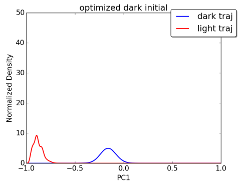
123	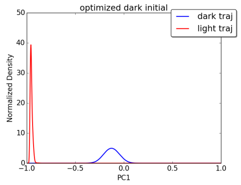	124	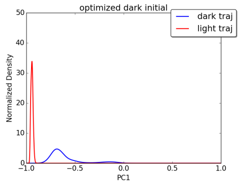
125	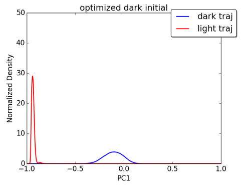	126	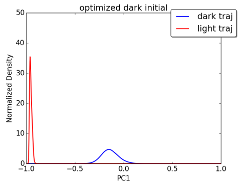

Table S7: Projections of all simulations onto the PC1 mode generated from principal component analysis (PCA) of the unperturbed Light state simulation with optimized Dark state structure as reference.

Residue	Distribution	Residue	Distribution
127	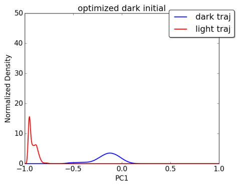	128	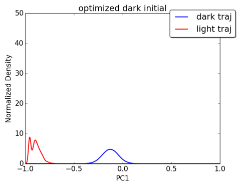
129	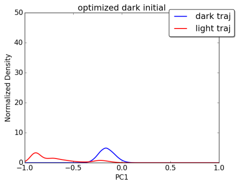	130	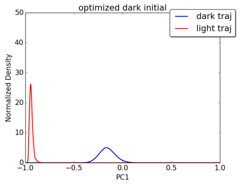
131	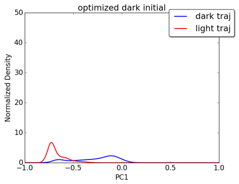	132	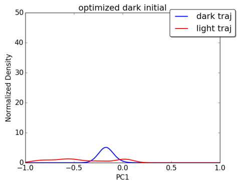
133	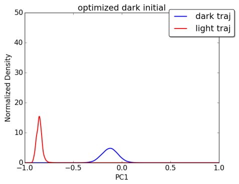	134	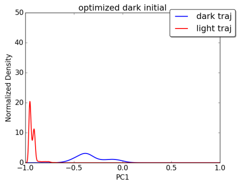
135	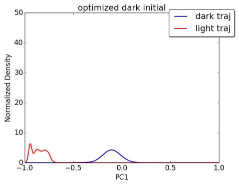	136	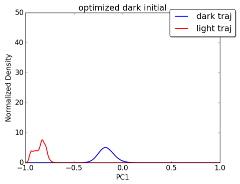
137	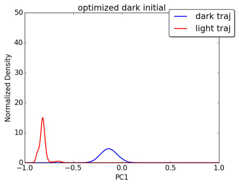	138	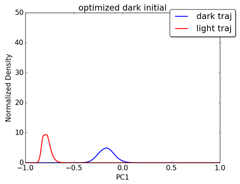
139	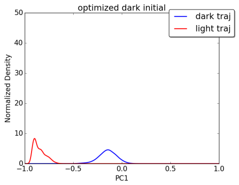	140	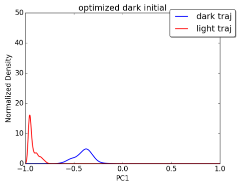
141	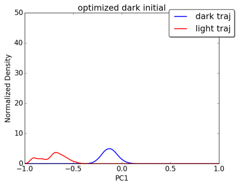	142	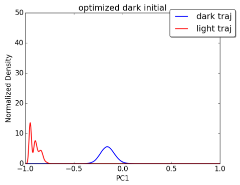

Table S7: Projections of all simulations onto the PC1 mode generated from principal component analysis (PCA) of the unperturbed Light state simulation with optimized Dark state structure as reference.

Residue	Distribution	Residue	Distribution
143	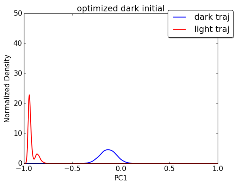	144	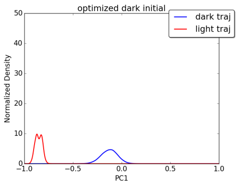
145	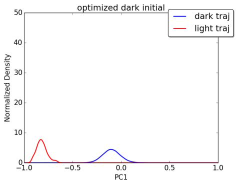	146	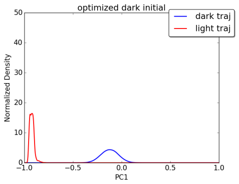
147	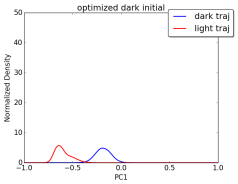	148	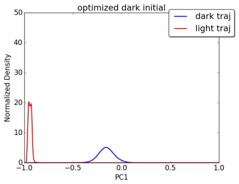
